# Supplementary material for: REST/NRSF drives homeostatic plasticity of inhibitory synapses in a target-dependent fashion
Source: eLife. 2021 Dec 2;10:e69058. doi: 10.7554/eLife.69058 (PMC8639147; doi:10.7554/eLife.69058)
Supplement: Figure 1—source data 1. [file elife-69058-fig1-data1.pdf]

Figure 1

	Figure 1B		Figure 1E (upper panel)		Figure 1E (lower panel)	
	Cy3-ODN internalization (%)		Inhibitory Neurons		Excitatory Neurons	
			Cy3-area (nucleus) / Cy3-area (cytosol)		Cy3-area (nucleus) / Cy3-area (cytosol)	
	12h	24h	ODN/veh	ODN/4AP	ODN/veh	ODN/4AP
	100	73	0.4232	0.6813	0.2186	0.5185
	75	68	0.2887	0.1973	0.4938	0.6179
	80	98	0.0000	0.0597	0.0300	0.3832
	50	100	0.0000	0.6287	0.0420	0.5473
	60	97	0.0000	1.4871	0.5763	0.5207
	98	99	0.0000	1.3467	0.0340	0.9376
	67	74	0.2080	1.3278	0.2926	0.5838
	71	75	0.1693	0.4810	0.0450	0.0566
	67	57	0.0382	0.5683	0.0227	0.5014
	50	80	0.4169	0.0550	0.1401	0.5253
	33	88	0.7149	0.1204	0.2021	0.2138
	50	97	0.6115	0.4328	0.3611	0.3311
	95	76	0.0917	1.4009	0.5771	0.6673
	71	94	0.2956	0.1399	0.2761	0.3207
	50	92	0.2497	0.5597	0.4761	0.5632
	48	84	0.2177	0.2090	0.5865	0.9700
	43	82	0.5061	0.7482	0.1213	0.5520
			0.0000	0.2036	0.0525	0.5463
			0.0653	0.5033	0.5205	0.6813
			0.1512	1.5633	0.2431	0.2592
			0.0314	0.7394	0.0543	0.6894
			0.0950	0.6128	0.5508	1.1568
			0.8051	0.5884	0.0343	0.5701
			0.7600	1.0270	0.4047	0.5023
			0.2733	0.5290	0.1230	1.5038
			0.0000	0.8977		1.4000
			0.0385	0.9030		0.2544
			0.0675	0.5471		0.4563
			0.0225	0.4175		0.4028
			0.5376	0.5087		0.2155
			0.7670	0.3934		0.2609
			0.1130	0.9077		0.5399
			0.1899	0.6944		
				0.3384		
**N**	**17**	**17**	**33**	**34**	**25**	**32**
**Media**	**65.21**	**84.35**	**0.25**	**0.64**	**0.26**	**0.57**
SD	19.89	12.66	0.26	0.41	0.21	0.32
**SE**	**4.82**	**3.07**	**0.04**	**0.07**	**0.04**	**0.06**

Figure 1

Figure 1 B	
**Unpaired t test**	
P value	0.0021
P value summary	**
Significantly different (P < 0.05	Yes
One- or two-tailed P value?	Two-tailed
t, df	t=3,349, df=32

Figure 1 E *(upper panel)*				
**Mann-Whitney's U-test**				
n=34	n=35	Significant	Summary	P Value
ODN:veh	ODN:4AP	Yes	***	<0.0001

Figure 1 E *(lower panel)*				
**Mann-Whitney's U-test**				
n=25	n=31	Significant	Summary	P Value
ODN:veh	ODN:4AP	Yes	***	<0.0001
