# Supplementary material for: REST/NRSF drives homeostatic plasticity of inhibitory synapses in a target-dependent fashion
Source: eLife. 2021 Dec 2;10:e69058. doi: 10.7554/eLife.69058 (PMC8639147; doi:10.7554/eLife.69058)
Supplement: Figure 1—figure supplement 2—source data 1. [file elife-69058-fig1-figsupp2-data1.pdf]

Figure 1- figure supplement 2

HCN1 RNA fold change			
NEG/vel	NEG/4AP	ODN/veh	ODN/4AP
0.7876	0.518522	0.9469154	1.201
1.2124	0.424612	1.225927	0.938
0.7587	0.535854	1.0477886	0.898
1.4759	0.655346	1.3264	1.147
0.8466	0.457	0.784665	1.214
1.0658	0.6434	1.075767	0.790
6	6	6	6
1.02	0.54	1.07	1.03
0.28	0.09	0.19	0.18
0.12	0.04	0.08	0.07

Syn1 RNA fold change			
NEG/veh	NEG/4AP	ODN/veh	ODN/4AP
0.841145	0.554092	1.21699	0.8249429
1.158855	0.6385	1.33644	1.094857
1	0.6473	1.3	1.55542
1	0.44305	0.82161	1.20145
0.9675	0.82346	1.19345	1.04757
0.9987	0.756373	1.4312	1.2342
6	6	6	6
0.99	0.64	1.22	1.16
0.10	0.14	0.21	0.24
0.04	0.06	0.09	0.10

Nav1.2 RNA fold change			
NEG/veh	NEG/4AP	ODN/veh	ODN/4AP
1.140399	0.288165	1.49	1.048666
1.187243	0.397543	1.006755	1.436058
0.672358	0.341796	0.803899	0.498752
0.80395	0.7759	1.35322	1.2863
1.19605	0.44144	0.7	0.96271
1	0.74175	1.48	1.14133
1	0.64165	1.32801	1.12166
7	7	7	7
1.00	0.52	1.17	1.07
0.20	0.20	0.33	0.30
0.08	0.07	0.12	0.11

Figure 1-suppl. fig 2

Figure 1-suppl. fig 2B			
HCN1 RNA fold change			
**two-way ANOVA/Tukey's tests**			
Tukey's multiple compari	Significant	Summary	P Value
NEG:4AP vs. NEG:veh	Yes	**	0.0022
ODN:veh vs. NEG:veh	No	ns	0.9811
ODN:4AP vs. NEG:veh	No	ns	>0,9999
ODN:veh vs. NEG:4AP	Yes	***	0.0009
ODN:4AP vs. NEG:4AP	Yes	**	0.0019
ODN:4AP vs. ODN:veh	No	ns	0.9883
Syn1 RNA fold change			
**two-way ANOVA/Tukey's tests**			
Tukey's multiple compari	Significant	Summary	P Value
NEG:veh vs. NEG:4AP	Yes	*	0.0159
NEG:veh vs. ODN:veh	No	ns	0.1818
NEG:veh vs. ODN:4AP	No	ns	0.4144
NEG:4AP vs. ODN:veh	Yes	***	0.0001
NEG:4AP vs. ODN:4AP	Yes	***	0.0005
ODN:veh vs. ODN:4AP	No	ns	0.9477
Nav1.2 RNA fold change			
**two-way ANOVA/Tukey's tests**			
Tukey's multiple compari	Significant	Summary	P Value
NEG:veh vs. NEG:4AP	Yes	*	0.0106
NEG:veh vs. ODN:veh	No	ns	0.6402
NEG:veh vs. ODN:4AP	No	ns	0.9567
NEG:4AP vs. ODN:veh	Yes	***	0.0006
NEG:4AP vs. ODN:4AP	Yes	**	0.0031
ODN:veh vs. ODN:4AP	No	ns	0.9032
