# Supplementary material for: REST/NRSF drives homeostatic plasticity of inhibitory synapses in a target-dependent fashion
Source: eLife. 2021 Dec 2;10:e69058. doi: 10.7554/eLife.69058 (PMC8639147; doi:10.7554/eLife.69058)
Supplement: Figure 2—source data 1. [file elife-69058-fig2-data1.pdf]

Figure 2B (left panel; MFR)

NEG/veh		
hr 1	hr 24	hr 48
105.2446	164.9447	167.98
125.6564	137.7235	189.03
157.1789	48.6559	202.4959
189.12	234.56	110.76
111	135.19	153.8385
112	111.0764	174.8027
109.09	101.7656	156
128.3674	74.7676	123.44
71.393	122.8159	115.3291
132.09	210.04	112.22
89.6686	67.4801	119.4368
88.8147	90.413	84.473
61.4303	127.4517	131.9252
73.449	89.9352	79.0287
36.119	99.6713	72.6763
114.01	60.9405	118.947
76.5597	100.1324	69.2544
70.3597	76.4999	73.9212
51.628	81.6932	40.4043
110.34	102.3734	60.9275
106.1888	158.9611	90.8789
47.1094	103.7348	79.9504
143.45	201.03	165.09
189	110.4279	115.542
100.34	113.471	79.9906
78.98	49.4696	75.5984
121.04	188.88	167.87
171.6677	189.07	12.7651
54.7213	83.0217	102.1941
41.2667	48.3305	101.9144
44.1932	53.444	73.4208
130.34	84.4233	98.6844
92.6253	128.1545	114.0681
75.441	114.4052	89.9112
84.4635	134.8654	131.069
140.8055	118.4955	165.5071
91.481	121.8006	126.235
116.0636	115.9691	152.7756
45.4233	139.9016	145.7292
120.9817	134.5031	123.994
124.9518	155.9372	146.87
99.2781	110.5992	142.3376
145.9878	126.7124	95.9231
127.6863	176.98	97.6905
149.215	211.2644	163.2399
137.921	102.7912	91.1992
99.04	88.9616	142.9835
47	47	47
**104.1102404**	**119.2284085**	**115.9643128**
38.03872991	45.26792836	40.15341775
**5.548519015**	**6.603005986**	**5.856977939**

NEG/4AP		
hr 1	hr 24	hr 48
245.9414	161.2685	286.8807
360.952	101.8332	98
133.124	340.826	99
136.7992	186.5923	229.6774
181	121	110
123	197.6085	89
357.0104	205.8582	109
145.1781	166.9212	208.6995
187	123	99
329.4858	119	107
148.591	118	66.8154
151.2994	139.3338	131.8015
135.6596	36.5841	157.2689
201.3644	140.3087	210.7834
175.5496	131.2219	161.9984
135.1532	82.7107	101.9291
213.6503	156.0745	167.2996
159.9783	112.3369	110.5994
182.7575	101.0332	98.6787
130.0889	191.5092	171.8313
188.5555	75.2613	103.3268
145.0024	87.9921	100.1062
141.6655	118.3038	120.0774
194.7727	129.1842	158.1862
202.213	116.6935	107.2432
127.6337	83.9086	59.0582
127.6061	76.4027	71.3037
368.3117	285.6152	166.91
193.7973	113.8296	75.8025
153.5341	192.1746	47.2777
147.6646	61.0915	91.7339
147.7024	106.0614	242.8418
164.9366	83.4195	97.1944
141.0503	112.4377	69.3716
247.5654	136.4564	30.161
211.9197	132.1992	34.8471
207.3111	110.1087	87.1038
161.6048	128.7518	25.1785
243.0011	129.4595	44.169
148.008	89.6922	83.2764
171.2383	90.1553	106.7939
147.0792	95.5771	55.1533
150.6299	89.8897	75.3815
246.0271	321.0451	278.1578
132.2508	47.4263	31.5712
142.9853	87.8354	70.7676
182.1299	134.8872	17.5815
233.2668	28.3474	43.0407
166.8037	100.0528	53.9618
168.0699	106.0616	77.1274
132.5855	125.6506	5.382
177.98	156.99	42.7533
145.98	44.3104	41.7194
211.87	90.8633	105.378
149.0373	54.6054	40.8862
144.5022	110.0275	108.2336
169.7364	109.5356	91.1781
124.9238	71.529	79.0271
172.3261	126.5771	138.1307
180	120	100
143.8152	128.0317	96.9659
330.7628	220.5001	76.0237
291.2146	151.2442	135.1949
63	63	63
**184.3278397**	**125.6064635**	**103.187973**
60.73661395	58.88038689	60.12314241
**7.652094094**	**7.418231467**	**7.574803946**

ODN/veh		
hr 1	hr 24	hr 48
85.5607	112.3827	155.2421
129.4201	113.6344	155
130.9341	156.78	167.45
147.6837	144.87	189
126.1156	132.56	114.89
120.01	139.4264	159.4386
117.6514	119.212	134
121.01	92.1503	118.1838
99.5403	151.1303	157.9278
86.6734	102.639	103.2726
112	123.5144	115.0277
47.4037	94.7572	111.3863
110.88	78.6745	102.2643
41.0615	61.3955	88.3652
82.417	132.8834	94.639
101.09	84.4264	76.3895
113.0468	86.7998	110.103
70.626	114.6633	76.2128
117.9459	142.1346	149.5771
123.5086	131.6473	144.4547
96.6368	157.414	146.89
185.9578	95.1448	70.3309
126.6002	148.8217	159.4785
110.82	89.9192	147.8337
155.1592	79.9296	127.2278
117.8715	179.5515	162.9504
112.861	129.2912	135.5074
133.45	146.8885	122.846
124.2582	134.0129	110.0629
120.3033	146.182	132.2404
120.7693	124.88	152.23
98.7493	138.0061	71.8678
121.3444	90.0718	91.4109
172.82	137.5819	124.6947
132.55	180.15	124.0524
84.6287	125.0438	94.5333
90.8556	96.4066	121.708
137.3592	137.4093	128.8282
134.3035	102.8418	61.3484
110.33	108.0757	164.0301
72.1528	107.8338	111.5512
145.76	160.985	195.8415
123.89	186.5726	189.9
72.1834	129	133.34
98.2351	108.6152	83.0988
76.5171	102.281	104.3441
103.09	126.0198	131.7851
48.21	117.6072	43.3232
32.8426	73.9184	11.3841
114.5969	131.3868	59.7596
127.43	122.0291	112.89
51	51	51
**109.551269**	**122.148094**	**120.5904686**
30.8036453	28.1312971	38.1141122
**4.31337311**	**3.9391695**	**5.337043223**

ODN/4AP		
hr 1	hr 24	hr 48
154.7117	281.6531	156.45
226.8399	229.09	123.98
145.8889	218.56	245.93
136.504	234.98	210.76
233.9275	118.729	121.2385
269.2727	248.7992	234.76
121.6472	190.7958	175.7461
194.2104	95.0379	120.5983
312.9065	257.482	208.1975
165.87	176.89	187.76
136.5095	91.5746	128.0169
121.9918	89.0912	81.7893
142.4436	74.52	56.3558
136.8988	79.9159	74.8681
215.6145	150.1199	143.3622
130.6968	68.2105	65.6495
149.6423	145.0469	138.7605
210.9539	134.0948	182.6131
206.869	155.4518	205.6966
183.385	146.6717	164.5736
253.6413	221.0764	272.3485
136.396	263.9148	232.6834
304.2289	233.8428	249.451
138.3778	125.0275	150.033
197.899	153.3947	176.55
165.0043	211.9709	231.1054
154.3366	236.1201	278.88
261.628	334.4838	199.8745
152.7281	87.5517	91.6599
224.5566	142.2937	114.8733
427.7429	280.709	294.2388
151.0613	121.3022	105.9915
131.1168	84.1645	41.8795
157.7512	115.4431	96.3667
144.5593	95.1282	95.1229
161.0707	102.1665	41.5483
187.0071	129.4933	69.3514
197.3034	72.399	10.6324
140.9494	88.0026	56.9189
165.8137	90.9271	48.7823
315.5265	322.0682	265.0884
331.0319	110.141	163.7317
395.7128	288.3475	54.87
229.7232	98.5137	145.67
188.56	488.2892	178.6029
347.338	317.2121	144.2847
135.4327	125.5416	184.6664
222.9722	178.9707	175.2102
204.296	100.7392	80.0182
147.4933	149.751	99.5638
376.7215	324.0011	115.6269
261.5923	156.1382	205.2399
221.303	113.7433	113.3505
141.2495	126.8684	91.8896
54	54	54
**203.128**	**171.7861**	**146.3558**
75.2812	88.08114	69.99701
**10.24447**	**11.98633**	**9.525387**

Figure 2B (center panel; BF)

NEG/veh		
hr 1	hr 24	hr 48
133.5871	24.9466	156.9086
113.1229	149.5317	164.9426
115.8466	111.2417	177.6475
154.3004	67.3881	222.9089
79.1278	129.9092	111.5669
134.1621	91.2802	164.6246
83.8512	78.6106	58.8456
48.8655	85.1534	103.7074
146.8531	152.0448	92.2382
112	58.4895	67.6296
52.1422	50.5527	42.7612
37.4429	66.4935	63.7212
44.7903	71.7274	35.8925
25.4113	56.2841	42.5475
105.0674	123.28	70.6176
46.9288	90.1874	51.5612
58.9269	118.543	110.4104
155	88.4294	55.7786
123	111.7354	64.0297
120	124	47.7302
52.9348	57.1628	66.0312
125.3093	121.9485	122.2126
77.741	90.3177	61.897
123	161.4551	158.5892
149.8143	143.8445	189.0058
157.0109	119.9728	261.6487
78.4436	135.8596	144.9088
81.4742	154.4144	154.5061
118.1443	150.9739	171.3222
112.1431	176.877	228.752
114.7095	119.7504	128.9068
115.7635	109.0956	182.3071
142.5155	141.3941	113.0031
112.2969	130.3724	145.3397
117.6541	78.4615	81.1355
91.3828	171.0364	125.5053
140.6418	110.8655	107.2449
101.89	73.743	126.091
163.9588	161.9906	68.5957
108.1348	126	17.2929
125.547	187	62.2819
146.0778	169.6982	83.5233
85.0133	102.2228	61.4909
134	182.0749	132.7171
166.4641	95.5174	28.7822
100	134	182.8794
43.9601	72.7612	48.1706
47	47	47
**105.882**	**113.3753**	**109.7917**
40.57393	62.33078	84.35085
**4.594093**	**7.057571**	**9.550853**

NEG/4AP		
hr 1	hr 24	hr 48
327.9474	248.0558	346.384
389.6396	147.2915	145
208.7804	256.0829	310.676
456.4487	172.3484	147
455.0284	237.3354	116
159.5758	169.5378	207.3214
455.2977	198	167
525.8203	178	156
172.5523	476.019	71.9531
155.651	167.8894	128.9379
190.6983	48.7187	144.4091
250.9842	144.5791	163.476
222.2354	137.7417	108.3066
270.3475	208.682	148.6269
380.3602	234.1983	156.949
301.5825	205.4172	122.3937
298.5709	131.7423	116.8857
167.6983	231.585	198.2648
361.7516	102.7743	128.5363
214.1948	129.7334	128.5411
217.0997	160.6087	138.053
286.8696	198.0245	216.2336
368.1158	156.034	136.5795
294.6323	129.4331	85.0422
184.8677	76.9697	71.0754
470.4768	269.5181	191.2318
332.0263	150.4549	88.643
518.0676	201	66.7114
278.0854	147.4902	140.9066
309.3482	204.7087	114.5725
560.0086	232.0317	38.439
144.4827	265.4426	150.2704
560.4641	354.5631	62.057
434.5395	206.64	121.6157
327.6423	273.3442	28.5048
638.6082	299.3043	73.8245
252.9595	139.5778	143.8936
243.876	103.5292	134.3839
374.7387	222.4516	102.1911
359.1551	176.2684	148.2955
189.4763	334.3612	364.7855
297.2351	97.6261	51.8013
350.1437	181.5988	130.8601
384.3786	276.9455	11.1914
576.1709	49.6029	67.1352
286.955	248.9843	105.6858
267.1785	114.0435	67.0706
252.7819	244.5364	2.0288
257.9336	120.9972	122.0103
238.6582	163.1446	118.9594
249.4051	95.7495	68.7856
193.3121	79.3788	47.581
414.6177	110.7196	65.9046
249.1415	154.2911	126.4255
368.5248	147.8971	158.0137
322.7387	145.4529	142.3213
195.8306	98.8529	203.625
386.1715	191.7321	217.7289
381.8849	255.5078	80.5103
366.3826	294.3525	107.0364
730.9594	440.0662	207.2876
213.4347	123.3178	75.8879
271.2574	173.088	177.2019
63	63	63
**326.4413**	**189.4504**	**129.921**
141.4115	86.62709	71.12177
**17.27616**	**10.58318**	**8.688907**

ODN/veh		
hr 1	hr 24	hr 48
87.7279	88.575	130.9563
127.3099	77.3224	118.7025
146.0889	69.8798	135.1013
82.7881	101.1837	104.86
99.4771	148.5332	146.23
109.2698	141.6574	121.8425
144.0356	150.0374	180.6299
87.8546	116.9039	108.9328
56.8119	94.8704	62.9403
59.7335	79.7239	55.9853
38.0084	56.7046	53.2042
69.037	84.3481	92.2325
93.745	69.3149	68.3941
84.7646	45.2511	67.5582
29.3846	50.922	53.2355
35.464	60.4891	53.5096
100.8148	84.8187	66.8551
59.8072	97.3099	75.7953
39.0819	71.5514	64.3075
117.6257	143.3433	69.7446
95.033	129.2772	138.229
139.45	122.2069	98.4613
196.34	140.5254	78.6166
93.745	66.475	63.8161
184.75	145	47.9244
119.0957	96.1621	78.4734
61.3327	79.9663	41.9445
119.1014	152.3073	176.4634
121.5835	142.8055	132.9
112.3714	130.4702	144.572
181.3341	99.1798	70.0289
64.7384	80.7957	163.3623
103.3411	96.1322	126.4743
168.1802	103.3365	162.5983
130.1493	176.2095	121.8506
76.0508	124.9784	119.3553
127.9745	163.2939	150.0898
106.9538	135.93	124.7072
123.9463	114.205	135.9623
129.744	193.2616	157.0906
193.645	198.6956	62.5091
124.5604	95.2704	89.8297
106.2013	161.4052	118.2492
91.5707	107.9052	75.3331
99.2311	120.2444	155.3218
137.5785	118.5365	99.0661
121.7118	89.2071	40.3534
122.1115	103.9043	185.3336
164.8	199	67
102.8	98.0273	114.6125
137.45	69.6309	135.7838
51	51	51
**108.3471765**	**111.5115**	**104.0653**
45.56737524	49.54679	59.81038
**5.15948925**	**5.574449**	**6.772192**

ODN/4AP		
hr 1	hr 24	hr 48
338.9	234.6	198.45
300.6593	309.069	415.1577
273.3963	224.95	198.5
300.6566	224.573	198.57
182.828	547.4363	198.9985
285.4301	153.6049	136.6398
164.3054	251.8873	493.2047
153.1045	342.662	233.3428
402.2044	168.7123	296.1011
824.4072	185.86	447.7147
158.1728	137.4225	110.0238
227.9188	155.6482	153.9993
236.9011	123.2721	63.7733
273.1792	130.2697	73.7286
274.4808	209.914	131.6922
350.8587	178.7764	176.5611
307.8805	271.9607	257.6997
403.3855	187.3861	241.7815
291.1212	234.3986	240.567
162.2617	149.6875	144.4184
562.8714	314.8179	363.3785
172.5273	220.0487	210.6973
303.97	257.385	272.6748
330.1135	196.3488	142.7235
294.8058	227.35	208.7743
662.3052	278.586	194.876
210.0533	108.5329	112.0157
462.1894	241.4968	181.9923
341.0192	274.2561	283.9057
181.5141	228.2138	192.7065
205.0143	161.0488	2.4057
324.0079	203.3291	189.3615
227.888	129.0783	134.9116
367.1357	216.0178	53.9645
257.0656	135.3754	203.7
270.1449	125.9959	8.0641
187.5692	112.4843	70.8362
242.0296	122.4072	62.8258
449.8201	331.7907	193.038
348.7132	172.7521	192.4959
223.8531	244.1257	135.2449
254.0097	234.3765	332.0858
580.87	260.2721	302.9128
185.1078	91.0298	93.2776
282.6644	156.2531	69.3018
371.1201	269.3862	188.3591
486.2746	280.0488	193.1372
144.5985	156.5062	75.9144
387.6	209.98	222.819
228.5912	104.776	50.7509
117.0421	55.7759	40.4175
315.9123	162.6262	122.964
470.0211	175.0681	59.6245
888.1779	440.1534	379.4426
54	54	54
**319.4195**	**209.6256**	**184.306**
206.4463	104.6468	122.9106
**25.80579**	**13.08085**	**15.36382**

Figure 2B (right panel; BD)

NEG/veh		
hr 1	hr 24	hr 48
111.1185	114.1024	107.065
78.5252	95.9395	85.7867
127.135	247.2708	224.0465
55.9099	150.6087	169.2309
143.6748	164.7704	378.2454
75.6866	77.6465	62.6013
136.1919	144.57	134.5124
59.3979	50.9717	64.1695
78.305	88.6932	104.211
94.8924	102.0509	100.7848
97.6416	110.938	116.2128
91.0349	65.8197	93.6995
102.948	105.7932	152.9857
170.3598	126.5874	135.8863
96.5976	88.5384	103.7769
134.947	112.98	126.73
65.527	93.2283	101.7195
77.5444	101.0481	131.872
89.0547	125.1054	141.2217
139.286	131.4698	133.3148
104.0399	111.7703	157.577
92.3317	102.6776	149.8961
83.5709	115.339	95.2637
86.4159	122.0923	140.0755
128.028	116.836	129.65
75.4725	67.9996	82.3603
102.0305	102.8211	103.6347
101.5406	137.2721	128.034
101.3411	118.854	105.8374
107.0946	130.5526	113.1247
131.9974	127.7771	125.0392
115.1659	108.2628	97.248
99.3918	117.0043	119.8679
83.8479	139.2363	126.0616
101.023	112.935	131.0085
94.3663	100.2991	135.9403
104.1809	92.3532	84.1946
84.2178	64.6475	126.3294
124.6406	85.3518	128.3945
111.1473	108.7822	105.1674
106.2463	148.933	102.65
123.973	111.93	129.997
77.369	49.1228	82.3059
90.0677	111.482	111.1209
97.5114	104.0208	128.2077
132.083	102.9849	97.484
93.6433	80.7567	89.1757
47	47	47
**101.6706**	**110.4304**	**123.2706**
206.4463	104.6468	122.9106
**25.80579**	**13.08085**	**15.36382**

NEG/4AP		
hr 1	hr 24	hr 48
82.7707	62.1009	91.3006
110.0626	66.6962	17.947
72.8875	100.6418	122.5906
85.6322	103.1397	91.5015
64.5273	59.5606	66.8653
86.0398	153.5939	105.0584
65.5446	97.7515	128.0973
53.5523	61.1005	96.2001
48.3574	34.6169	46.322
49.3544	66.9761	73.6618
86.6413	107.738	70.6296
94.9332	104.5388	107.9527
67.899	74.3634	63.9974
87.9922	103.0103	168.8257
67.6059	44.6729	57.9723
55.7393	59.8546	41.9304
50.7833	66.043	39.6635
58.8079	75.1702	131.452
81.6463	92.8701	111.5206
59.8026	56.0518	77.757
58.6221	68.7911	82.3001
85.1113	85.899	79.7751
73.999	137.8614	182.7751
102.3148	66.6769	77.7533
74.3476	159.1924	97.5619
74.679	80.3832	95.0321
53.1785	71.2428	59.9516
56.2858	63.1201	96.7648
56.1931	82.3677	114.6812
44.4412	90.799	165.5654
66.9358	94.4899	121.6107
116.7897	140.1918	108.2988
46.1886	60.4506	83.9193
52.6373	60.8277	91.2543
41.6778	71.5097	135.694
57.1396	69.9919	100.1345
65.7024	75.9725	90.2455
90.9945	126.1607	122.8022
47.9409	52.4321	88.9215
52.6938	67.7326	71.5143
66.853	74.9671	101.2054
71.1409	95.6544	96.8074
38.6302	56.5836	75.5019
53.8189	84.5259	95.2229
60.6601	96.3196	91.1714
93.888	115.5932	139.8212
75.0926	40.1821	42.3002
61.3707	85.2294	89.5527
41.0096	24.2185	31.5231
50.0459	94.2661	109.7822
51.9594	74.3834	89.3361
50.6471	45.2282	58.681
79.9107	72.377	84.7796
29.0348	54.3129	52.8489
88.081	131.2808	168.411
97.9034	122.536	157.2205
61.4228	54.7054	201.3235
78.6339	69.7266	113.0916
80.1112	82.1599	100.9986
38.5733	120.0165	36.3096
75.857	203.9553	283.9705
95.3467	130.5987	183.5768
55.3733	49.4961	59.2157
63	63	63
**67.36218**	**84.04605**	**99.05481**
18.94091	32.84018	45.41117
**2.38633**	**4.137473**	**5.721269**

ODN/veh		
hr 1	hr 24	hr 48
89.9434	136.7929	132.1395
73.2026	84.9099	83.3402
66.387	81.6301	89.3643
100.983	128.5119	122.7434
177.1917	186.244	182.3052
95.5174	115.7595	138.032
90.5131	108.6532	150.6328
152.0761	85.0264	134.5371
96.6742	143.6887	184.5436
79.8515	61.9165	137.9925
86.9488	137.089	142.1573
87.2551	123.8246	142.6556
84.7663	102.78	138.2662
75.7428	95.675	143.0314
99.0188	113.5968	154.5087
101.2848	136.2517	161.305
120.9374	102.9387	133.9837
93.282	154.3845	128.4709
83.3848	132.1489	141.5763
105.4351	118.7509	113.0988
98.4043	125.4514	87.5601
89.4654	149.1943	152.0592
71.3001	109.6077	74.3838
89.0424	146.8501	142.7359
148.1851	113.9708	103.7815
72.8818	87.4724	83.6617
71.8403	114.6426	97.8725
79.9556	122.6003	141.9267
156.9836	102.8374	134.937
136.897	104.6079	166.2793
124.787	108.1415	105.9797
127.3344	155.8561	102.7366
99.0186	149.6931	161.9832
176.972	102.82	130.983
108.3309	91.9289	98.0213
98.7283	100.9841	114.9314
122.2007	136.8127	158.647
69.6859	104.1997	118.005
95.0071	70.0971	74.1483
83.3514	102.1703	125.3062
71.3395	79.8031	74.22
85.3209	104.4399	150.5726
69.6087	77.5122	90.2027
178.8897	115.3721	158.7748
89.6095	106.3772	102.8111
85.3016	77.3805	83.1725
117.4946	97.3211	116.5581
111.4038	109.5653	109.4631
84.3674	112.7701	134.3434
88.9404	105.1263	129.5878
101.1258	109.8054	112.8228
51	51	51
**101.2582**	**112.6664**	**125.3559**
28.74507	24.61031	28.29259
**4.025115**	**3.446133**	**3.961754**

ODN/4AP		
hr 1	hr 24	hr 48
52.1323	91.5203	74.8994
45.58	327.1143	270.1199
64.3841	202.1314	139.7878
70.9719	126.726	166.1956
112.3343	119.8177	123.0978
99.4834	103.9925	126.0696
86.8143	99.8472	83.6621
39.6182	39.6283	32.7345
85.4955	147.3582	121.7317
68.6205	90.4839	126.3033
59.7205	91.3179	53.602
95.17	138.3155	149.8048
57.2999	74.9236	104.9254
94.2832	89.1005	96.1256
90.5665	98.9374	108.4275
81.5258	68.3476	107.5856
80.0905	128.7738	135.7282
58.1079	85.04	136.8599
90.5755	99.7501	112.1287
98.3845	95.9675	66.0334
111.8815	158.0974	145.58
71.9338	39.3312	62.2489
31.6448	79.2511	26.3716
103.8136	75.8364	58.1896
67.598	112.3336	134.9044
89.586	89.7893	89.3107
90.4466	128.315	119.509
56.0302	82.7659	103.4457
98.745	150.8203	161.1075
29.8566	84.3061	76.0863
58.8086	107.7073	203.4362
53.4973	106.9767	89.4986
58.8204	68.5485	82.8992
56.8719	58.4841	91.4146
54.3051	99.1665	156.384
46.3039	82.6096	104.927
81.9875	114.8278	103.365
131.496	99.0563	158.6365
60.3888	76.7494	55.3924
115.8986	110.0863	149.8794
112.9315	83.7013	102.0676
104.2448	120.8564	157.0652
66.4958	79.0225	89.6977
58.4263	92.6834	182.2378
138.2603	169.4981	160.1205
83.848	325.5768	396.5624
65.8183	103.0391	139.8296
47.842	68.8849	105.7828
88.7326	164.8835	223.2516
50.3295	115.605	240.9213
58.771	51.7818	92.6083
89.747	293.7516	339.5418
72.7157	54.3	59.8573
97.698	107.6057	111.7149
54	54	54
**76.60989**	**112.5063**	**127.9568**
24.71113	59.75411	68.71394
**3.362759**	**8.131504**	**9.350783**

Figure 2C (left panel)

MFR BIC % change

	NEG/veh	NEG/4AP	ODN/veh	ODN/4AP
	-16.7899	50.3695	-33.2128	-34.7999
	5.5644	23.3822	-10.1037	-20.8865
	37.6116	21.1923	-24.1392	27.5941
	115.7898	65.7683	-23.5944	-25.4637
	132.0879	30.051	-32.3681	-27.0927
	42.827	23.0803	-17.3272	-26.383
	-3.98375	41.0668	-15.5324	-22.9973
	9.6394	32.4965	11.0388	-33.1206
	13.0149	39.3212	-28.1305	-27.7198
	59.7567	16.7538	-27.4955	-35.1317
	7.6474	-7.60606	-35.8911	-19.205
	121	-17.2913	27.5613	-6.40388
	22.8966	0.568	46.7968	74.524
	-5.56967	29.173	62.1345	-11.7243
	-4.39326	17.0942	114.0033	-31.3944
	16.3142	20.5628	-15.7705	0.9182
	-11.9445	141.8568	114	56.553
	-9.79749	57.3756	23.3116	11.0581
	22.5105	101.0421	24.2463	78.7431
	41.2794	26.1268	125.7915	-23.1619
	6.4291	44.0456	95.7865	-2.90997
	7.5505	77.5484	57.9751	62.8988
	7.2356	160.6385	24.9341	23.2403
	86.4946	-1.45353	97.0112	194.7601
	117.7051	186.1648	6.0208	77.541
	129.0049	123.9079	112.0031	-1.91388
	49.243	183.9074	36.3778	157.2953
	37.6991	188	81.1592	122.1579
	26.0286	78.8238	-13.6494	6.9272
	99.2171	77.1146	124.4908	162.6606
	25.8953	188.4443	16.8516	146.3645
	105.388	61.8855	33.9916	101.322
	123.0658	53.9804	23	22.5733
	18.7791	30.0743	62.6106	31.0599
	-9.04345	25.9153	53.6848	-6.4245
	48.2198	145.2468	67.0766	82.3876
	3	142.1032	128.3806	51.096
	66.0571	26.0227	45.4089	21.3075
	49.7238	-24.7418	16.7285	39.5514
	9.2153	166.0522	102.2665	-9.86061
	131.3459	-3	35.8216	-8.43083
	3.6497	-10	-24.0802	-15.5521
	1	3.0145	126.2682	20.7766
	1	105.7121	-47.262	-17.6793
	-26.8315	130.1646	49.0689	44.8909
	7.4857	164.8004	-3.17855	15.5307
	18.1924	1.4613	31.7462	25.2941
	-26.9617	2	-19.2386	-15.4596
	-31.3669	45.2871	5.5389	-35.9426
	29.7751	138.963	-5.21209	27.6309
	2	7.2841	61.1316	116.0537
	-30.5981	-10.0838	134.4007	13.7769
	-2	-4	105.5118	41.6364
	2	87.1311	1.4195	109.5305
	-21.241	63.9368	4	30.5854
	2	39.9557	102.3492	24.2626
	94.712	83.1948	9.6772	64.9275
	-1.93015	38.1978	33	-2.15576
	95.984	-8	130.9553	-4.06507
	0.7106	189	-19.6782	21.1115
	100	-1.83536	93.2881	59.6538
	-0.09081	68.1264		14.9354
	49.8788	184.54		76.8733
	-16.1526			114.8856
	97.2661			171.7598
	**65**	**63**	**61**	**65**
**N**	**32.03380185**	**62.88754**	**37.09763**	**32.01185538**
**Media**	47.0096678	63.89186	53.07009	57.31321426
SD	**5.830831669**	**8.049618**	**6.794929**	**7.108829319**

Figure 2C (center panel)

BF BIC % change

NEG/veh	NEG/4AP	ODN/veh	ODN/4AP
-53.6801	52.33446	-36.9299	-7.04308
-30.84	83.70162	7.69051	39.88193
-9.51614	59.35976	-21.1801	23
-16.8889	120	-49.2194	36.19657
-59.8899	63.48531	-47.7875	123
-55.2071	60.40749	-73.1165	116.2297
-60.6236	30.45204	-49.3484	16.62109
-65.013	59.30336	-30.1226	-0.02011
-3.67014	39.09763	-23.4002	-11.753
-16.5944	20.33035	-57.0318	26.18194
-43.7883	3.592724	-70.6048	73.24885
-28.5734	5	1.312626	-8.9583
25.01513	6.032782	34.61387	45
-21.232	-16.8931	33.86164	48.73743
-50.5953	6.213491	116.8965	-16.8528
19.13991	4.670698	-24.5246	7.473772
72.22881	61.33411	148.9663	24.61405
14.98416	100.9127	-42.5623	-5.95043
42.7526	42.25254	5.359579	-17.7769
-37.0075	102.86	171.0738	-3.08458
-12.2802	53.06349	55.66888	58.84345
-9.88854	83.26998	24.51917	-4.00828
102.7037	223.9117	5.782906	24.92717
66.59683	256.6805	7.280206	33.55143
58.10661	13.474	149.3414	134.2868
38.29227	234	1.420918	255.5466
116.2561	123	199.246	24
183.1975	234	23.32383	123
255.0696	256	185.0978	8.989234
59.42043	87.59456	300.1809	95.72425
37.01371	213.2201	25.77355	-75.5434
0.767312	31.07472	12.53547	113.6369
269.3433	-6.61944	271.964	34.1943
130.6024	203.5716	75.62096	75.61374
106.0956	5.654455	63.56422	5.706137
-23.342	234	77.17106	1
-17.0488	95.63722	-31.0892	34
8.982057	-3.00297	126.06	265.1694
-29.4123	247.0916	-51.6842	-56.0977
-35.6	5	153.5544	99.81027
149.0782	-51.4588	-17.8901	0.985717
-10.1027	-24.964	67.04632	5
-9.9406	94.26227	-27.7914	-36.1917
107.4929	144.721	-30.4114	23.43772
6.334167	94.16416	-25.1476	44.55448
7.632404	312.314	123	7
-60.1592	-22.8589	9.478923	-12
-59.7796	95.34418	59.04025	12
21.05044	83.28172	123.9553	109.9304
133.0222	-22.228	-44.3021	-30.1615
-40.4633	-21.4213	89.87402	7.07166
-2.5976	-33.0065	93.43541	-41.298
70.45285	40.46735	1.052717	90.38432
-2.78123	1.264497	106.0102	66.20147
-3.00758	52.1132	71.71717	5.521476
57.87509	-27.5727	111	
-34.8934	345	19.99976	
-29.4832	360.714	134	
346.216	5	34	
22.90717	88.05068		
60.90091			
244.9168			
**62**	**60**	**59**	**55**
**30.653986**	**83.47094**	**43.51435**	**36.6096814**
88.031667	101.4548	84.56358	64.6682372
**11.180033**	**13.09776**	**11.00924**	**8.71986333**

Figure 2C (right panel)

BD BIC % change

NEG/veh	NEG/4AP	ODN/veh	ODN/4AP
18.78356	60.44214	17.32133	193.0036
11.98153	115.3267	43.51722	37.32906
102.9404	122.5611	5.694644	136.9098
12.76549	105.0036	117.6875	18.34529
10.40868	41.43717	75.33269	54.01092
120.6278	200.6584	189	63.93876
5.862581	108.9047	121.8747	94.95657
132.6481	104.414	143.3477	56.38091
51.76993	11.23619	12.49883	104.1711
84.141	3.8316	164.6235	102.3618
20.20146	30.4206	195.7343	39.4032
203.3671	155.6128	149.2573	43.63682
45.25701	42.30292	150.5977	16.48389
244.1619	28.53386	8.73718	51.53527
193.3532	288.3855	130.5016	96.777
35.80591	40.97981	103.9598	13.56087
2.552392	56.5934	9.959691	65.5963
147.5301	76.39295	88.00707	69.52012
84.6346	114.5781	49.40043	43.76967
16.17972	30.64546	82.39506	121.4117
22.17779	82.582	86.9872	49.34282
75.31413	91.75941	53.53713	1.318207
3.72433	201.4064	90.8732	55.16588
38.9911	25.31147	178.2691	100.7616
1.293772	50.25831	100.7081	19.14741
54.45684	33.97296	129.168	17.60893
75.32083	39.1023	140.7984	90.63238
109.377	82.25034	67.874	70.88282
94.99287	170.7237	7.043781	35.76147
12.79832	57.81426	9.98819	34.08949
-2.93548	119.8089	171.9072	10.41829
39.08543	8.102166	89.97823	202
52.52641	7.28543	115.3131	135.233
231.625	36.23075	46.29773	74.0846
120.787	6.343008	48.89113	73.972
34.6363	1.612186	88.624	72.987243
83.0475	87.8743	29.61417	71.87324
3.943086	220.6728	39.31034	14.36276
211.206	64.47135	91.03635	55.96174
82.51797	8.130923	50.84758	77.87274
101.8255	47.51446	26.40918	91.31266
77.94774	36.44291	87.7645	46.27923
76.50473	34.70466	16.17536	217.2609
156.2964	72.8734	193.6174	161.1302
104.9311	73.843	10.80146	150.9456
80.44341	73.57794	85.45984	
24.7117	71.487		
6.083195	101.2077		
38.59285	26.30891		
135.3927	2.32092		
246.3733	59.43363		
148.3845	34.81765		
115.0292			
**53**	**52**	**46**	**45**
**79.29009408**	**72.47127**	**85.14659**	**74.52239689**
68.1585988	60.97452	57.14855	51.90566797
**9.362303569**	**8.455645**	**8.426095**	**7.737640134**

Figure 2

Figure 2B			
MFR % change (left panel)			
**two-way ANOVA/Tukey's tests**			
Tukey's multiple comparisons test	Significant	Summary	P Value
**1hr**			
NEG/veh vs. NEG/4AP	Yes	****	<0.0001
NEG/veh vs. ODN/veh	No	ns	0.9646
NEG/veh vs. ODN/4AP	Yes	****	<0.0001
NEG/4AP vs. ODN/veh	Yes	****	<0.0001
NEG/4AP vs. ODN/4AP	No	ns	0.2798
ODN/veh vs. ODN/4AP	Yes	****	<0.0001
**24hr**			
NEG/veh vs. NEG/4AP	No	ns	0.937
NEG/veh vs. ODN/veh	No	ns	0.9942
NEG/veh vs. ODN/4AP	Yes	****	<0.0001
NEG/4AP vs. ODN/veh	No	ns	0.9882
NEG/4AP vs. ODN/4AP	Yes	****	<0.0001
ODN/veh vs. ODN/4AP	Yes	****	<0.0001
**48hr**			
NEG/veh vs. NEG/4AP	No	ns	0.6464
NEG/veh vs. ODN/veh	No	ns	0.9777
NEG/veh vs. ODN/4AP	Yes	*	0.037
NEG/4AP vs. ODN/veh	No	ns	0.3624
NEG/4AP vs. ODN/4AP	Yes	***	0.0003
ODN/veh vs. ODN/4AP	No	ns	0.0928

Figure 2B			
BF % change (center panel)			
**two-way ANOVA/Tukey's tests**			
Tukey's multiple comparisons tes	Significant	Summary	P Value
**1hr**			
NEG/veh vs. NEG/4AP	Yes	****	<0.0001
NEG/veh vs. ODN/veh	No	ns	0.9989
NEG/veh vs. ODN/4AP	Yes	****	<0.0001
NEG/4AP vs. ODN/veh	Yes	****	<0.0001
NEG/4AP vs. ODN/4AP	No	ns	0.9703
ODN/veh vs. ODN/4AP	Yes	****	<0.0001
**24hr**			
NEG/veh vs. NEG/4AP	Yes	****	<0.0001
NEG/veh vs. ODN/veh	No	ns	0.9995
NEG/veh vs. ODN/4AP	Yes	****	<0.0001
NEG/4AP vs. ODN/veh	Yes	****	<0.0001
NEG/4AP vs. ODN/4AP	No	ns	0.5748
ODN/veh vs. ODN/4AP	Yes	****	<0.0001
**48hr**			
NEG/veh vs. NEG/4AP	No	ns	0.6075
NEG/veh vs. ODN/veh	No	ns	0.9872
NEG/veh vs. ODN/4AP	Yes	****	<0.0001
NEG/4AP vs. ODN/veh	No	ns	0.3694
NEG/4AP vs. ODN/4AP	Yes	**	0.0033
ODN/veh vs. ODN/4AP	Yes	****	<0.0001

Figure 2B			
BD % change (right panel)			
**two-way ANOVA/Tukey's tests**			
Tukey's multiple comparisons test	Significant	Summary	P Value
**1hr**			
NEG/veh vs. NEG/4AP	Yes	****	<0.0001
NEG/veh vs. ODN/veh	No	ns	>0.9999
NEG/veh vs. ODN/4AP	Yes	**	0.0075
NEG/4AP vs. ODN/veh	Yes	****	<0.0001
NEG/4AP vs. ODN/4AP	No	ns	0.5792
ODN/veh vs. ODN/4AP	Yes	**	0.0072
**24hr**			
NEG/veh vs. NEG/4AP	Yes	**	0.0028
NEG/veh vs. ODN/veh	No	ns	0.9921
NEG/veh vs. ODN/4AP	No	ns	0.9934
NEG/4AP vs. ODN/veh	Yes	***	0.0007
NEG/4AP vs. ODN/4AP	Yes	***	0.0006
ODN/veh vs. ODN/4AP	No	ns	>0.9999
**48hr**			
NEG/veh vs. NEG/4AP	Yes	**	0.0075
NEG/veh vs. ODN/veh	No	ns	0.9936
NEG/veh vs. ODN/4AP	No	ns	0.9318
NEG/4AP vs. ODN/veh	Yes	**	0.0022
NEG/4AP vs. ODN/4AP	Yes	***	0.0004
ODN/veh vs. ODN/4AP	No	ns	0.9864

Figure 2C			
MFR BIC % change (left panel)			
**two-way ANOVA/Tukey's tests**			
Tukey's multiple comparisons tes	Significant	Summary	P Value
NEG:4AP vs. NEG:veh	Yes	*	0.0103
ODN:veh vs. NEG:veh	No	ns	0.9565
ODN:4AP vs. NEG:veh	No	ns	>0,9999
ODN:veh vs. NEG:4AP	No	ns	0.0507
ODN:4AP vs. NEG:4AP	Yes	*	0.0102
ODN:4AP vs. ODN:veh	No	ns	0.956
BF BIC % change (center panel)			
**two-way ANOVA/Tukey's tests**			
Tukey's multiple comparisons tes	Significant	Summary	P Value
NEG:4AP vs. NEG:veh	Yes	**	0.0045
ODN:veh vs. NEG:veh	No	ns	0.8189
ODN:4AP vs. NEG:veh	No	ns	0.9872
ODN:veh vs. NEG:4AP	No	ns	0.0632
ODN:4AP vs. NEG:4AP	Yes	*	0.0179
ODN:4AP vs. ODN:veh	No	ns	0.9553
BD BIC % change (right panel)			
**two-way ANOVA/Tukey's tests**			
Tukey's multiple comparisons tes	Significant	Summary	P Value
NEG:4AP vs. NEG:veh	No	ns	0.9055
ODN:veh vs. NEG:veh	No	ns	0.9421
ODN:4AP vs. NEG:veh	No	ns	0.9842
ODN:veh vs. NEG:4AP	No	ns	0.6365
ODN:4AP vs. NEG:4AP	No	ns	0.9925
ODN:4AP vs. ODN:veh	No	ns	0.8226
