# Supplementary material for: REST/NRSF drives homeostatic plasticity of inhibitory synapses in a target-dependent fashion
Source: eLife. 2021 Dec 2;10:e69058. doi: 10.7554/eLife.69058 (PMC8639147; doi:10.7554/eLife.69058)
Supplement: Figure 3—source data 1. [file elife-69058-fig3-data1.pdf]

Figure 3

Figure 3C				Figure 3D				Figure 3G				Figure 3H			
Frequency (Hz)				Amplitude (pA)				Frequency (Hz)				Amplitude (pA)			
NEG/veh	NEG/4AP	ODN/veh	ODN/4AP	NEG/veh	NEG/4AP	ODN/veh	ODN/4AP	NEG/veh	NEG/4AP	ODN/veh	ODN/4AP	NEG/veh	NEG/4AP	ODN/veh	ODN/4AP
3.135	6.978	3.451	4.165	34.965	30.899	20.960	36.513	2.606	1.583	3.137	2.184	21.935	22.060	22.409	42.291
5.350	6.756	5.088	5.925	43.381	16.222	26.478	46.228	1.993	2.232	2.443	5.000	15.473	19.030	25.769	23.527
5.272	9.294	4.624	5.557	30.113	29.874	29.042	46.478	2.595	2.673	2.666	3.301	28.875	20.814	28.230	47.000
3.105	9.159	4.217	5.000	23.742	29.077	47.000	45.291	2.018	2.567	1.609	3.976	37.537	24.000	27.550	33.880
6.346	9.000	5.024	6.968	38.530	39.297	23.220	33.099	5.070	2.233	3.214	3.254	33.790	24.000	30.631	17.073
4.841	9.471	4.365	5.522	30.838	35.612	28.000	44.532	1.227	2.351	2.034	3.700	22.975	32.000	19.650	23.623
6.123	8.924	4.304	5.058	37.142	30.739	44.000	44.440	3.795	3.029	2.717	2.584	22.800	48.000	23.480	18.775
6.319	8.126	4.176	3.959	39.972	25.639	23.629	33.594	4.084	3.541	3.299	3.000	41.216	28.279	37.088	22.962
5.807	7.076	5.223	5.282	32.678	39.583	18.760	39.090	3.296	2.518	3.698	2.000	30.004	9.311	32.880	27.000
5.144	5.738	3.513	5.166	29.080	36.847	33.000	26.379	3.504	2.119	1.656	4.500	17.373	34.000	26.020	37.000
5.467	9.809	6.938	6.956	21.340	39.231	33.000	48.265	3.207	4.021	2.098	3.300	17.975	26.843	21.558	17.000
6.043	6.652	4.331	3.804	38.110	30.306	15.939	23.961	1.847	3.309	1.800	4.200	10.444	10.744	16.398	17.320
5.197	6.000	4.668	6.245	23.031	44.884	35.000	18.853	2.985	4.286	2.560	2.450	22.152	19.808	26.040	21.050
3.863	8.378	7.244	4.265	19.596	14.971	45.000	28.096								
6.964	4.758	4.359	1.729	22.930	38.802	23.759	13.187								
5.215	8.400	4.757	5.979	27.839	32.246	35.000	36.953								
4.377	9.406	5.436	4.000	25.942	27.058	20.459	45.213								
5.554	7.752	4.872	6.803	18.258	22.645	28.573	41.076								
5.000	6.865	5.123	7.739	21.431	17.065	34.000	29.778								
6.723	7.009	4.623	5.099	24.982	22.123	33.000	21.560								
5.390	7.545	5.103	5.124	29.564	26.840	28.560	23.390								
**N**	**21**	**21**	**21**	**21**	**21**	**21**	**21**	**21**	**13**	**13**	**13**	**13**	**13**	**13**	**13**	**13**
**Media**	**5.30**	**7.77**	**4.83**	**5.25**	**29.21**	**30.00**	**29.83**	**34.57**	**2.94**	**2.80**	**2.53**	**3.34**	**24.81**	**24.53**	**25.98**	**26.81**
SD	1.03	1.39	0.91	1.35	7.30	8.32	8.50	10.51	1.05	0.79	0.67	0.91	9.01	10.04	5.56	10.07
**SE**	**0.23**	**0.30**	**0.20**	**0.30**	**1.59**	**1.82**	**1.86**	**2.29**	**0.29**	**0.22**	**0.19**	**0.25**	**2.50**	**2.79**	**1.54**	**2.79**

Figure 3

Figure 3C

two-way ANOVA/Tukey's tests

Tukey's multiple comparisons te	Significant	Summary	P Value
NEG:veh vs. NEG:4AP	Yes	****	<0,0001
NEG:veh vs. ODN:veh	No	ns	0.5847
NEG:veh vs. ODN:4AP	No	ns	0.9994
NEG:4AP vs. ODN:veh	Yes	****	<0,0001
NEG:4AP vs. ODN:4AP	Yes	****	<0,0001
ODN:veh vs. ODN:4AP	No	ns	0.6571

Figure 3D

two-way ANOVA/Tukey's tests

Tukey's multiple comparisons te	Significant	Summary	P Value
NEG:veh vs. NEG:4AP	No	ns	0.9913
NEG:veh vs. ODN:veh	No	ns	0.9958
NEG:veh vs. ODN:4AP	No	ns	0.2015
NEG:4AP vs. ODN:veh	No	ns	>0,9999
NEG:4AP vs. ODN:4AP	No	ns	0.3327
ODN:veh vs. ODN:4AP	No	ns	0.3007

Figure 3G

two-way ANOVA/Tukey's tests

Tukey's multiple comparisons te	Significant	Summary	P Value
NEG:ctrl vs. NEG:4AP	No	ns	0.9782
NEG:ctrl vs. ODN:ctrl	No	ns	0.6316
NEG:ctrl vs. ODN:4AP	No	ns	0.642
NEG:4AP vs. ODN:ctrl	No	ns	0.8549
NEG:4AP vs. ODN:4AP	No	ns	0.3998
ODN:ctrl vs. ODN:4AP	No	ns	0.0952

Figure 3H

two-way ANOVA/Tukey's tests

Tukey's multiple comparisons te	Significant	Summary	P Value
NEG:veh vs. NEG:4AP	No	ns	0.9998
NEG:veh vs. ODN:veh	No	ns	0.9868
NEG:veh vs. ODN:4AP	No	ns	0.9393
NEG:4AP vs. ODN:veh	No	ns	0.9754
NEG:4AP vs. ODN:4AP	No	ns	0.9132
ODN:veh vs. ODN:4AP	No	ns	0.9951
