# Supplementary material for: REST/NRSF drives homeostatic plasticity of inhibitory synapses in a target-dependent fashion
Source: eLife. 2021 Dec 2;10:e69058. doi: 10.7554/eLife.69058 (PMC8639147; doi:10.7554/eLife.69058)
Supplement: Figure 4—source data 1. [file elife-69058-fig4-data1.pdf]

Figure 4

Figure 4B (upper panel)
eIPSCs (nA)

	NEG/veh	NEG/4AP	ODN/veh	ODN/4AP
	2.500	3.830	4.630	3.09
	3.870	2.390	5.890	5.21
	2.480	6.200	6.410	3.45
	2.420	5.850	5.420	5
	2.490	4.360	5.490	3.29
	3.350	3.660	3.580	2.812
	5.370	4.030	2.250	5.31
	5.730	6.110	2.160	5.11
	2.340	5.710	1.950	5.2
	2.100	4.100	4.700	3.5
	5.720	6.000	4.500	2.6
	3.500	5.750	5.110	4
	5.090	5.300	3.800	4.2
	3.820	4.100	3.120	4.01
	3.240	5.740	3.440	3.18
	5.930	3.040	3.260	4.37
	3.830	3.160	3.130	3.95
	3.300	5.010	3.730	5.01
	4.100	2.620	1.790	3.1
	2.410	4.310	2.200	5.32
	2.060	3.750	4.480	3.37
	3.670	4.030	3.320	3.83
	4.160	4.890	3.200	3.02
	2.200	7.190	5.590	3.09
	3.200	3.680	3.750	
	3.560	7.570	4.020	
	4.010	6.340	3.830	
	3.520	6.240	3.350	
	4.270	5.600	3.580	
	2.180	4.980		
	5.960	4.760		
	3.290			
**N**	**32**	**31**	**29**	**24**
**Media**	**3.61**	**4.85**	**3.85**	**3.96**
SD	1.19	1.31	1.22	0.90
**SE**	**0.21**	**0.23**	**0.23**	**0.18**

Figure 4B (lower panel)
PPR (I_2/I_1)

	NEG/veh	NEG/4AP	ODN/veh	ODN/4AP
	0.143	0.136	0.358	0.227
	0.287	0.298	0.360	0.255
	0.376	0.138	0.210	0.323
	0.469	0.128	0.224	0.261
	0.598	0.429	0.245	0.339
	0.283	0.281	0.284	0.264
	0.442	0.230	0.229	0.605
	0.399	0.272	0.258	0.305
	0.362	0.273	0.400	0.229
	0.216	0.175	0.204	0.348
	0.319	0.240	0.221	0.266
	0.423	0.315	0.221	0.234
	0.205	0.215	0.223	0.601
	0.450	0.359	0.370	0.464
	0.262	0.270	0.355	0.457
	0.180	0.226	0.234	0.280
	0.496	0.345	0.455	0.312
	0.239	0.141	0.440	0.342
	0.353	0.210	0.307	0.370
	0.428	0.310	0.605	0.416
	0.180	0.312	0.466	0.311
	0.275	0.375	0.485	0.296
	0.466	0.249	0.371	0.335
	0.498	0.216	0.244	0.276
	0.417	0.269	0.328	
	0.147	0.175	0.261	
	0.363	0.163	0.397	
	0.457	0.124	0.205	
	0.371	0.076	0.326	
	0.570	0.212		
	0.257	0.314		
	0.641			
**N**	**32**	**31**	**29**	**24**
**Media**	**0.36**	**0.24**	**0.32**	**0.34**
SD	0.13	0.08	0.10	0.10
**SE**	**0.02**	**0.02**	**0.02**	**0.02**

Figure 4F
RRPsyn I(nA)

	NEG/veh	NEG/4AP	ODN/veh	ODN/4AP
	2.660	5.930	5.390	3.460
	2.380	9.100	8.480	3.510
	5.200	8.190	6.260	5.270
	5.220	5.340	1.480	6.040
	2.580	3.540	2.860	4.130
	4.240	7.910	1.400	1.550
	6.060	7.290	1.930	3.120
	2.860	6.180	4.910	7.840
	6.590	3.070	3.190	2.590
	3.430	3.870	2.490	1.650
	5.060	3.590	2.120	7.240
	1.500	4.830	5.320	6.810
	7.820	8.160	2.170	4.850
	5.330	4.620	6.980	5.680
	7.480	6.390	4.700	3.870
	5.500	9.640	4.950	5.920
	6.370	7.890	5.660	7.060
	7.710	7.200	4.480	4.340
	4.000	9.610	4.080	5.550
	3.980		6.120	5.580
	2.660		4.540	5.330
	3.980			
**N**	**22**	**19**	**21**	**21**
**Media**	**4.66**	**6.44**	**4.26**	**4.83**
SD	1.85	2.12	1.93	1.77
**SE**	**0.39**	**0.49**	**0.42**	**0.39**

Figure 4G
Pr

	NEG/veh	NEG/4AP	ODN/veh	ODN/4AP
	0.530	0.618	0.552	0.572
	0.500	0.673	0.688	0.598
	0.473	0.576	0.340	0.374
	0.672	0.766	0.388	0.567
	0.512	0.509	0.669	0.591
	0.535	0.627	0.479	0.584
	0.596	0.590	0.415	0.640
	0.497	0.534	0.376	0.308
	0.317	0.647	0.594	0.463
	0.406	0.565	0.498	0.592
	0.315	0.614	0.441	0.586
	0.480	0.466	0.391	0.468
	0.617	0.486	0.536	0.390
	0.364	0.666	0.626	0.350
	0.542	0.591	0.642	0.580
	0.448	0.768	0.460	0.430
	0.500	0.763	0.410	0.590
	0.559	0.541	0.580	0.600
	0.408	0.573	0.630	0.340
	0.332		0.590	0.470
	0.468		0.500	0.570
	0.547			
**N**	**22**	**19**	**21**	**21**
**Media**	**0.48**	**0.61**	**0.51**	**0.51**
SD	0.10	0.09	0.11	0.10
**SE**	**0.02**	**0.02**	**0.02**	**0.02**

Figure 4

Figure 4B (upper panel)			
**two-way ANOVA/Tukey's tests**			
Tukey's multiple comparisons test	Significant	Summary	P Value
NEG:veh vs. NEG:4AP	Yes	***	0.0004
NEG:veh vs. ODN:veh	No	ns	0.8619
NEG:veh vs. ODN:4AP	No	ns	0.6999
NEG:4AP vs. ODN:veh	Yes	**	0.0075
NEG:4AP vs. ODN:4AP	Yes	*	0.032
ODN:veh vs. ODN:4AP	No	ns	0.9872
Figure 4B (lower panel)			
**two-way ANOVA/Tukey's tests**			
Tukey's multiple comparisons test	Significant	Summary	P Value
NEG :Veh vs. NEG :4AP	Yes	***	0.0001
NEG :Veh vs. ODN:Veh	No	ns	0.4388
NEG :Veh vs. ODN:4AP	No	ns	0.8498
NEG :4AP vs. ODN:Veh	Yes	*	0.0263
NEG :4AP vs. ODN:4AP	Yes	**	0.0065
ODN:Veh vs. ODN:4AP	No	ns	0.9304
Figure 4F			
**two-way ANOVA/Tukey's tests**			
Tukey's multiple comparisons test	Significant	Summary	P Value
NEG :Veh vs. NEG :4AP	Yes	*	0.0208
NEG :Veh vs. ODN:Veh	No	ns	0.9017
NEG :Veh vs. ODN:4AP	No	ns	0.9922
NEG :4AP vs. ODN:Veh	Yes	**	0.0032
NEG :4AP vs. ODN:4AP	Yes	*	0.0462
ODN:Veh vs. ODN:4AP	No	ns	0.7742
Figure 4G			
**two-way ANOVA/Tukey's tests**			
Tukey's multiple comparisons test	Significant	Summary	P Value
NEG :Veh vs. NEG :4AP	Yes	***	0.0007
NEG :Veh vs. ODN:Veh	No	ns	0.72
NEG :Veh vs. ODN:4AP	No	ns	0.8409
NEG :4AP vs. ODN:Veh	Yes	*	0.0184
NEG :4AP vs. ODN:4AP	Yes	**	0.0099
ODN:Veh vs. ODN:4AP	No	ns	0.9962

Figure 4d (inset of the left panel)			
**Multiple t tests**	Significant	Summary	P value
NEG:veh vs. NEG:4AP	Yes	**	0.002
NEG:veh vs. NEG:4AP	Yes	*	0.016
NEG:veh vs. NEG:4AP	Yes	*	0.048
NEG:veh vs. NEG:4AP	Yes	*	0.034
NEG:veh vs. NEG:4AP	No	ns	0.056
NEG:veh vs. NEG:4AP	Yes	*	0.038
NEG:veh vs. NEG:4AP	Yes	*	0.038
NEG:veh vs. NEG:4AP	No	ns	0.053
NEG:veh vs. NEG:4AP	Yes	*	0.046
NEG:veh vs. NEG:4AP	No	ns	0.105
