# Supplementary material for: REST/NRSF drives homeostatic plasticity of inhibitory synapses in a target-dependent fashion
Source: eLife. 2021 Dec 2;10:e69058. doi: 10.7554/eLife.69058 (PMC8639147; doi:10.7554/eLife.69058)
Supplement: Figure 4—figure supplement 1—source data 1. [file elife-69058-fig4-figsupp1-data1.pdf]

Figure 4-figure supplement 1

	**Figure4-fig suppl 1B (upper panel)** eIPSCs (nA)			
	NEG/veh	NEG/4AP	ODN/veh	ODN/4AP
	3.660	4.340	4.790	3.910
	3.760	3.710	4.650	2.630
	6.820	4.820	3.920	3.950
	2.250	4.150	4.410	3.690
	6.830	4.380	2.510	3.860
	5.820	4.740	4.650	2.630
	3.480	2.650	3.920	3.950
	2.860	6.890	4.410	3.690
	5.290	4.500	3.200	4.600
	4.500	5.000	4.200	4.000
	5.000	3.500	4.100	3.200
	6.000	3.000	3.800	3.700
	3.400	3.800	3.030	4.800
	3.200	4.800	5.800	5.000
	6.200	4.100	5.200	3.500
**N**	**15**	**15**	**15**	**15**
**Media**	**4.60**	**4.29**	**4.17**	**3.81**
SD	1.50	0.99	0.84	0.68
**SE**	**0.39**	**0.26**	**0.22**	**0.17**

	**Figure4-fig suppl 1B (lower panel)** PPR (I_2/I_1)			
	NEG/veh	NEG/4AP	ODN/veh	ODN/4AP
	0.372	0.518	0.444	0.304
	0.591	0.603	0.638	0.513
	0.564	0.409	0.517	0.370
	0.502	0.322	0.614	0.630
	0.642	0.425	0.633	0.322
	0.580	0.280	0.638	0.513
	0.511	0.590	0.517	0.370
	0.373	0.340	0.614	0.630
	0.191	0.400	0.171	0.570
	0.370	0.320	0.500	0.500
	0.400	0.390	0.420	0.530
	0.500	0.560	0.470	0.480
	0.460	0.500	0.350	0.430
	0.320	0.450	0.360	0.400
	0.330	0.480	0.300	0.340
**N**	**15**	**15**	**15**	**15**
**Media**	**0.45**	**0.44**	**0.48**	**0.46**
SD	0.12	0.10	0.14	0.11
**SE**	**0.03**	**0.03**	**0.04**	**0.03**

	**Figure4-fig suppl 1F** RRPsyn I(nA)			
	NEG/veh	NEG/4AP	ODN/veh	ODN/4AP
	6.080	5.940	9.950	8.140
	8.750	7.330	8.120	6.660
	6.300	8.090	7.150	6.070
	5.550	5.130	7.800	6.450
	10.210	8.490	5.220	5.250
	9.930	2.760	6.700	6.400
	7.220	7.580	9.000	7.000
	5.180	6.000	8.500	7.400
	5.000	7.400	5.700	7.700
	7.300	7.900	6.000	8.500
	8.000	8.300	9.300	5.800
	6.900	8.000	8.000	5.700
	7.600	6.000	6.300	6.300
	8.400	5.000	6.000	6.900
	5.900	6.300	7.400	6.000
**N**	**15**	**15**	**15**	**15**
**Media**	**7.22**	**6.68**	**7.41**	**6.68**
SD	1.62	1.58	1.42	0.93
**SE**	**0.42**	**0.41**	**0.37**	**0.24**

	**Figure4-fig suppl 1G** Pr			
	NEG/veh	NEG/4AP	ODN/veh	ODN/4AP
	0.510	0.632	0.533	0.491
	0.279	0.482	0.573	0.426
	0.438	0.513	0.464	0.606
	0.496	0.454	0.565	0.436
	0.545	0.585	0.559	0.670
	0.441	0.591	0.500	0.450
	0.471	0.613	0.460	0.600
	0.552	0.460	0.550	0.570
	0.660	0.380	0.440	0.340
	0.450	0.580	0.490	0.730
	0.370	0.420	0.530	0.490
	0.510	0.470	0.590	0.560
	0.460	0.440	0.700	0.520
	0.330	0.530	0.470	0.500
	0.300	0.350	0.400	0.480
**N**	**15**	**15**	**15**	**15**
**Media**	**0.45**	**0.50**	**0.52**	**0.52**
SD	0.10	0.09	0.07	0.10
**SE**	**0.03**	**0.02**	**0.02**	**0.03**

Figure 4-figure supplement 1

Figure4-fig suppl 1B (upper panel)			
**two-way ANOVA/Tukey's tests**			
Tukey's multiple comparisons test	Significant	Summary	P Value
NEG :veh vs. NEG :4AP	No	ns	0.8457
NEG :veh vs. ODN:veh	No	ns	0.6726
NEG :veh vs. ODN:4AP	No	ns	0.1703
NEG :4AP vs. ODN:veh	No	ns	0.9893
NEG :4AP vs. ODN:4AP	No	ns	0.5869
ODN:veh vs. ODN:4AP	No	ns	0.7749
Figure4-fig suppl 1B (lower panel)			
**two-way ANOVA/Tukey's tests**			
Tukey's multiple comparisons test	Significant	Summary	P Value
NEG :veh vs. NEG :4AP	No	ns	0.9978
NEG :veh vs. ODN:veh	No	ns	0.8829
NEG :veh vs. ODN:4AP	No	ns	0.9906
NEG :4AP vs. ODN:veh	No	ns	0.7961
NEG :4AP vs. ODN:4AP	No	ns	0.9628
ODN:veh vs. ODN:4AP	No	ns	0.9722
Figure4-fig suppl 1F			
**two-way ANOVA/Tukey's tests**			
Tukey's multiple comparisons test	Significant	Summary	P Value
NEG :veh vs. NEG :4AP	No	ns	0.7235
NEG :veh vs. ODN:veh	No	ns	0.9833
NEG :veh vs. ODN:4AP	No	ns	0.7273
NEG :4AP vs. ODN:veh	No	ns	0.4987
NEG :4AP vs. ODN:4AP	No	ns	>0,9999
ODN:veh vs. ODN:4AP	No	ns	0.5027
Figure4-fig suppl 1G			
**two-way ANOVA/Tukey's tests**			
Tukey's multiple comparisons test	Significant	Summary	P Value
NEG :veh vs. NEG :4AP	No	ns	0.521
NEG :veh vs. ODN:veh	No	ns	0.1926
NEG :veh vs. ODN:4AP	No	ns	0.1618
NEG :4AP vs. ODN:veh	No	ns	0.916
NEG :4AP vs. ODN:4AP	No	ns	0.881
ODN:veh vs. ODN:4AP	No	ns	0.9997
