# Supplementary material for: REST/NRSF drives homeostatic plasticity of inhibitory synapses in a target-dependent fashion
Source: eLife. 2021 Dec 2;10:e69058. doi: 10.7554/eLife.69058 (PMC8639147; doi:10.7554/eLife.69058)
Supplement: Figure 5—source data 1. [file elife-69058-fig5-data1.pdf]

Figure 5

**Figure 5B**				
	Somatic Density N/µm2			
	NEG/veh	NEG/4AP	ODN/veh	ODN/4AP
	0.012	0.05	0.0228	0.03
	0.014	0.04	0.0128	0.028
	0.016	0.058	0.0324	0.024
	0.022	0.036	0.0134	0.01
	0.02	0.026	0.026	0.02
	0.012	0.057	0.0246	0.02
	0.01	0.0504	0.026	0.024
	0.024	0.028	0.016	0.03
	0.02	0.051	0.0122	0.026
	0.008	0.044	0.0104	0.026
	0.012	0.042	0.028	0.024
	0.018	0.0502	0.0136	0.024
	0.014	0.032	0.0128	0.022
	0.03	0.03	0.03	0.012
	0.018	0.058	0.022	0.022
	0.024	0.044	0.023	0.016
	0.022	0.026	0.03	0.01
	0.016	0.036	0.016	0.014
	0.01	0.03	0.0192	0.01
	0.022	0.04	0.01	0.018
	0.016	0.034	0.008	0.014
	0.018	0.034	0.0126	0.014
	0.0244	0.032	0.018	0.016
	0.014	0.038	0.018	0.01
	0.018	0.0556	0.0136	0.018
	0.016	0.046	0.014	
	0.028	0.034	0.0192	
	0.016	0.038	0.0186	
	0.022	0.03	0.02	
		0.034	0.016	
		0.046	0.0184	
		0.048	0.0122	
		0.038		
		0.034		
		0.048		
		0.044		
		0.024		
		0.048		
		0.03		
		0.036		
		0.0226		
		0.026		
		0.036		
		0.026		
		0.02442		
		0.024		
		0.028		
		0.05		
**N**	**29**	**48**	**32**	**25**
Media	0.017807	0.038275	0.01843125	0.01928
SD	0.005451	0.010106	0.006457226	0.006478
SE	0.001012	0.001459	0.001141487	0.001296

Figure 5

Figure 5B			
**two-way ANOVA/Tukey's tests**			
Tukey's multiple comp	Significant	Summary	P Value
NEG:veh vs. NEG:4A	Yes	****	<0,0001
NEG:veh vs. ODN:ve	No	ns	0.9895
NEG:veh vs. ODN:4A	No	ns	0.9004
NEG:4AP vs. ODN:ve	Yes	****	<0,0001
NEG:4AP vs. ODN:4/	Yes	****	<0,0001
ODN:veh vs. ODN:4A	No	ns	0.9771
