# Supplementary material for: REST/NRSF drives homeostatic plasticity of inhibitory synapses in a target-dependent fashion
Source: eLife. 2021 Dec 2;10:e69058. doi: 10.7554/eLife.69058 (PMC8639147; doi:10.7554/eLife.69058)
Supplement: Figure 5—figure supplement 1—source data 1. [file elife-69058-fig5-figsupp1-data1.pdf]

Figure 5 - figure supplement 1

Figure 5 - figure supplement 1B			
Dendritic Density N/µm			
NEG/veh	NEG/4AP	ODN/veh	ODN/4AP
0.152	0.12	0.194	0.162
0.154	0.148	0.196	0.254
0.188	0.218	0.172	0.278
0.16	0.122	0.244	0.192
0.192	0.124	0.21	0.266
0.208	0.09	0.2	0.22
0.176	0.166	0.192	0.19
0.174	0.202	0.146	0.25
0.198	0.14	0.236	0.15
0.2	0.142	0.15	0.218
0.228	0.192	0.224	0.284
0.23	0.16	0.178	0.196
0.214	0.136	0.308	0.236
0.258	0.152	0.182	0.144
0.186	0.12	0.29	0.2552
0.244	0.176	0.198	0.244
0.232	0.14	0.232	0.194
0.18	0.172	0.152	0.22
0.212	0.192	0.172	0.258
0.228	0.22	0.246	0.25
0.224	0.132	0.206	0.226
0.222	0.11	0.202	0.23
0.23	0.162	0.216	0.238
0.312	0.08	0.262	0.26
0.182	0.234	0.236	0.258
0.328	0.206	0.266	0.182
0.198	0.186	0.244	0.186
0.252	0.176	0.214	0.202
0.162	0.182	0.198	0.266
0.186	0.15	0.286	0.154
0.234	0.234	0.272	0.166
0.228	0.166	0.268	0.204
0.14	0.074	0.166	0.242
0.126	0.156	0.2	0.142
0.194	0.132	0.22	0.196
0.214	0.154	0.286	0.198
0.272	0.164	0.256	0.132
0.238	0.154	0.318	0.2938
0.258	0.15	0.254	0.26
0.214	0.18	0.322	0.222
0.204	0.226	0.246	0.22
0.22	0.148	0.192	0.182
0.216	0.194	0.246	0.196
0.25	0.188	0.184	0.216
0.282	0.24	0.25	0.236
0.242	0.212	0.22	0.158
0.266	0.156	0.248	0.21
0.262	0.166	0.234	0.186
0.21	0.172	0.148	0.278
0.21	0.2	0.23	0.252
0.25	0.166	0.3	0.252
0.254	0.208	0.256	0.212
0.208	0.236	0.2	0.254
0.26	0.182	0.246	0.216
0.224	0.216	0.212	0.194
0.278	0.12	0.252	0.24
0.234	0.22	0.276	0.26
0.192	0.12	0.294	0.236
0.206	0.186	0.23	0.202
0.216	0.204	0.224	0.22
0.198	0.136	0.25	0.214
0.192	0.236	0.27	0.164
0.274	0.248	0.238	0.132
0.334	0.16	0.258	0.15
0.222	0.19	0.212	0.142
0.244	0.236	0.162	0.19
0.23	0.17	0.18	0.214
0.286	0.13	0.238	0.212
0.264	0.122	0.22	0.264
0.214	0.174	0.176	0.186
0.272	0.08	0.204	0.26
	0.172	0.244	0.176
	0.092	0.116	
	0.132	0.138	
	0.178	0.134	
	0.14	0.132	
	0.226	0.206	
	0.18	0.136	
	0.18	0.17	
	0.222	0.174	
	0.172	0.2	
	0.176	0.146	
		0.208	
		0.174	
		0.164	
		0.188	
		0.242	
**71**	**82**	**87**	**72**
0.223127	0.16861	0.217034	0.214486
0.040936	0.040817	0.046311	0.040448
0.004858	0.004508	0.004965	0.004767

Figure 5 - figure supplement 1

Figure 5 - figure supplement 1B			
**two-way ANOVA/Tukey's tests**			
Tukey's multiple compari	Significant	Summary	P Value
NEG:Ctrl vs. NEG:4AP	Yes	****	<0,0001
NEG:Ctrl vs. ODN:Ctrl	No	ns	0.8053
NEG:Ctrl vs. ODN:4AP	No	ns	0.615
NEG:4AP vs. ODN:Ctrl	Yes	****	<0,0001
NEG:4AP vs. ODN:4AP	Yes	****	<0,0001
ODN:Ctrl vs. ODN:4AP	No	ns	0.9816
