# Supplementary material for: REST/NRSF drives homeostatic plasticity of inhibitory synapses in a target-dependent fashion
Source: eLife. 2021 Dec 2;10:e69058. doi: 10.7554/eLife.69058 (PMC8639147; doi:10.7554/eLife.69058)
Supplement: Figure 5—figure supplement 2—source data 1. [file elife-69058-fig5-figsupp2-data1.pdf]

Figure 5-figure supplement 2

Figure5-fig suppl 2B

Somatic Density N/µm2

	NEG/veh	NEG/4AP	ODN/veh	ODN/4AP
	0.0088	0.0158	0.0126	0.011
	0.0096	0.01382	0.0182	0.0184
	0.0108	0.0156	0.015	0.0056
	0.0124	0.0114	0.0124	0.0104
	0.0102	0.0132	0.0134	0.017
	0.0146	0.01579	0.0098	0.0126
	0.0094	0.0148	0.015	0.0094
	0.0084	0.01786	0.012	0.0144
	0.0068	0.0084	0.0128	0.0108
	0.0078	0.0122	0.0076	0.0162
	0.0122	0.0138	0.0144	0.0114
	0.012	0.0136	0.0132	0.007
	0.008	0.01574	0.0116	0.0148
	0.013	0.0152	0.0158	0.0152
	0.0182	0.013	0.0184	0.0134
	0.014	0.0088	0.0148	0.0116
	0.0146	0.0146	0.0114	0.0078
	0.0088	0.0144	0.0092	0.0102
	0.011	0.0126	0.0108	0.0084
	0.01	0.0116	0.0166	0.0086
	0.0124	0.0104	0.018	
		0.01		
		0.0102		
		0.015		
		0.0068		
		0.0134		
		0.0106		
N	21	27	21	20
Media	0.011095	0.012911	0.013476	0.01171
SD	0.002797	0.002658	0.002961	0.003492
SE	0.00061	0.000512	0.000646	0.000781

Figure5-fig suppl 2C

Dendritic Density N/µm

	NEG/veh	NEG/4AP	ODN/veh	ODN/4AP
	0.28	0.158	0.234	0.24
	0.302	0.186	0.184	0.2
	0.304	0.176	0.152	0.24
	0.258	0.218	0.202	0.262
	0.25	0.124	0.234	0.21
	0.262	0.216	0.218	0.194
	0.26	0.132	0.198	0.168
	0.192	0.158	0.232	0.222
	0.182	0.186	0.204	0.326
	0.142	0.15	0.232	0.19
	0.22	0.194	0.18	0.15
	0.198	0.192	0.204	0.188
	0.158	0.332	0.192	0.208
	0.22	0.186	0.15	0.356
	0.15	0.212	0.088	0.336
	0.188	0.188	0.176	0.232
	0.188	0.19	0.22	0.272
	0.19	0.118	0.264	0.266
	0.216	0.13	0.222	0.218
	0.23	0.19	0.2	0.182
	0.25	0.202	0.186	0.142
	0.25	0.154	0.258	0.144
	0.176	0.212	0.188	0.25
	0.226	0.216	0.2	0.264
	0.204	0.156	0.254	0.19
	0.232	0.14	0.108	0.196
	0.22	0.172	0.28	0.286
	0.214	0.18	0.244	0.292
	0.146	0.302	0.344	0.244
	0.19	0.206	0.282	0.226
	0.156	0.2	0.186	0.206
	0.144	0.18	0.304	0.19
	0.128	0.264	0.24	0.186
	0.228	0.18	0.144	0.26
	0.218	0.228	0.238	0.254
	0.214	0.266	0.276	0.264
	0.23	0.166	0.174	0.208
	0.262	0.188	0.258	0.164
	0.186	0.166	0.216	0.236
	0.186	0.184	0.26	0.178
	0.222	0.318	0.182	0.222
	0.298	0.276	0.112	0.242
	0.24	0.196	0.298	0.242
	0.222	0.222	0.262	0.22
	0.21	0.218	0.204	0.256
	0.216	0.18	0.204	0.22
	0.24	0.126	0.23	0.222
	0.22	0.258	0.2	0.234
	0.318	0.214	0.206	0.334
	0.238	0.278	0.204	0.246
	0.212	0.19	0.334	0.136
	0.284	0.344	0.164	0.156
	0.254	0.264	0.25	0.12
	0.268	0.29	0.276	0.18
	0.23	0.178	0.206	0.132
	0.192	0.252	0.252	0.168
	0.25	0.222	0.164	0.184
	0.226	0.244	0.184	0.164
	0.242	0.184	0.186	0.206
	0.2	0.234	0.196	0.152
	0.22	0.19	0.244	0.146
	0.224	0.24	0.122	0.166
	0.2	0.254	0.162	0.19
	0.236	0.238	0.2	0.14
	0.226	0.186	0.208	0.268
	0.202	0.22	0.2	0.14
	0.264	0.16	0.246	0.166
		0.24	0.186	0.172
		0.334	0.286	0.192
		0.35	0.162	0.192
		0.276	0.142	0.152
		0.31	0.14	0.208
		0.21	0.18	
		0.21	0.182	
		0.208	0.226	
		0.264		
		0.196		
		0.192		
		0.23		
		0.186		
		0.188		
		0.206		
		0.192		
		0.166		
		0.246		
		0.344		
		0.236		
N	67	87	75	72
Media	0.220955	0.21331	0.211013	0.211222
SD	0.040275	0.052848	0.049942	0.05181
SE	0.00492	0.005666	0.005767	0.006106

Figure 5-figure supplement 2

Figure5-fig suppl 2B

two-way ANOVA/Tukey's tests

Tukey's multiple comparisons	Significant	Summary	P Value
NEG:veh vs. NEG:4AP	No	ns	0.1597
NEG:veh vs. ODN:veh	No	ns	0.0524
NEG:veh vs. ODN:4AP	No	ns	0.9104
NEG:4AP vs. ODN:veh	No	ns	0.9137
NEG:4AP vs. ODN:4AP	No	ns	0.5193
ODN:veh vs. ODN:4AP	No	ns	0.2331

Figure5-fig suppl 2C

two-way ANOVA/Tukey's tests

Tukey's multiple comparisons	Significant	Summary	P Value
NEG:veh vs. NEG:4AP	No	ns	0.7758
NEG:veh vs. ODN:veh	No	ns	0.6278
NEG:veh vs. ODN:4AP	No	ns	0.6509
NEG:4AP vs. ODN:veh	No	ns	0.991
NEG:4AP vs. ODN:4AP	No	ns	0.9934
ODN:veh vs. ODN:4AP	No	ns	>0,9999
