# Supplementary material for: REST/NRSF drives homeostatic plasticity of inhibitory synapses in a target-dependent fashion
Source: eLife. 2021 Dec 2;10:e69058. doi: 10.7554/eLife.69058 (PMC8639147; doi:10.7554/eLife.69058)
Supplement: Figure 6—source data 1. [file elife-69058-fig6-data1.pdf]

Figure 6

Figure 6A (upper panel)

	ctrl	4AP 6h	4AP 24h	4AP 48h	4AP 96h
	0.911	0.855	1.165	1.273	1.212
	0.930	1.142	1.520	1.515	0.899
	1.042	1.187	1.195	1.303	1.277
	1.116	1.349	1.513	1.184	1.425
	0.918	1.369	1.262	1.176	1.082
	0.922	1.090	1.546	1.041	0.807
	1.006	1.328	1.472	1.167	0.871
	1.156	1.037			
		1.364			
		1.102			
		1.463			
		1.254			
		1.260			
		1.330			
**N**	**8**	**14**	**7**	**7**	**7**
**Media**	**1.0000**	**1.2237**	**1.3820**	**1.2369**	**1.0819**
SD	0.0967	0.1641	0.1671	0.1488	0.2333
**SE**	**0.0342**	**0.0439**	**0.0632**	**0.0563**	**0.0882**

Figure 6A (center panel)

	ctrl	4AP 6h	4AP 24h	4AP 48h	4AP 96h
	1.186	1.863	1.471	1.207	0.720
	0.982	2.129	1.309	0.912	1.143
	0.934	1.717	1.216	0.968	1.044
	0.899	1.904	1.493	0.690	1.024
	0.986	2.587		0.877	0.644
	1.003	2.129		0.744	0.642
	1.147	1.897		0.814	0.577
	0.863	2.193			
		1.925			
		2.202			
		2.306			
		1.703			
		1.710			
		1.518			
**N**	**8**	**14**	**4**	**7**	**7**
**Media**	**1.0000**	**1.9844**	**1.3723**	**0.8874**	**0.8277**
SD	0.1134	0.2875	0.1325	0.1703	0.2335
**SE**	**0.0401**	**0.0768**	**0.0662**	**0.0644**	**0.0883**

Figure 6A (lower panel)

	ctrl	4AP 6h	4AP 24h	4AP 48h	4AP 96h
	0.913	2.288	3.094	0.712	1.170
	1.104	3.119	1.593	1.121	1.215
	1.016	2.377	1.636	0.927	0.696
	0.941	2.765	2.054	1.375	0.764
	0.924	2.042	1.435	1.352	0.699
	0.901	2.578	1.217	1.143	0.714
	0.867	2.336	1.719	1.027	0.773
	1.334	3.154			
		3.770			
		3.203			
		3.956			
		3.138			
		3.154			
		3.313			
**N**	**8**	**14**	**7**	**7**	**7**
**Media**	**1.0000**	**2.9424**	**1.8210**	**1.0940**	**0.8615**
SD	0.1544	0.5662	0.6171	0.2334	0.2282
**SE**	**0.0546**	**0.1513**	**0.2332**	**0.0882**	**0.0863**

Figure 6B (upper panel)

	scrRNA			shRNA		
	ctrl	4AP 24h	4AP 48h	ctrl	4AP 24h	4AP 48h
	1.000	1.277	1.630	0.663	0.821	0.683
	0.994	1.123	1.400	0.678	0.748	0.790
	1.006	1.103	1.006	0.603	0.871	0.753
	0.994	1.419	1.127	0.609	0.867	0.756
	1.006	1.474	1.363	0.408	0.778	0.623
	1.017	1.486	1.291	0.475	1.020	0.711
	0.983	1.558	1.436	1.291	0.437	0.798
	1.155	2.034	1.435	0.432	0.817	0.872
	0.845	1.500		1.435	0.560	0.749
				0.606	0.751	0.802
**N**	**9**	**9**	**8**	**10**	**10**	**10**
**Media**	**1.0000**	**1.4416**	**1.3360**	**0.7200**	**0.7670**	**0.7537**
SD	0.0781	0.2772	0.1948	0.3530	0.1642	0.0696
**SE**	**0.0260**	**0.0924**	**0.0689**	**0.1116**	**0.0519**	**0.0220**

Figure 6B (lower panel)

scrRNA			shRNA		
ctrl	4AP 24h	4AP 48h	ctrl	4AP 24h	4AP 48h
1.000	2.374	1.338	0.963	2.064	1.124
0.796	3.519	1.263	1.037	1.924	1.033
1.204	2.583	1.648	0.915	3.793	2.532
0.907	6.248	2.120	1.085	0.914	2.647
1.093	5.132	0.779	0.439	1.958	3.038
0.942	5.888	0.812	1.561	2.343	2.919
1.058	3.445	0.667	1.311	3.643	5.953
0.875	1.863	1.087	0.689	1.688	6.424
1.125		0.990	0.940	1.657	1.370
			1.060		1.350
**9**	**8**	**9**	**10**	**9**	**10**
**1.0000**	**3.8815**	**1.1893**	**1.0000**	**2.2204**	**2.8390**
0.1316	1.6710	0.4661	0.3068	0.9355	1.9237
**0.0439**	**0.5908**	**0.1554**	**0.0970**	**0.3118**	**0.6083**

Figure 6D (left panel)

eIPSCs (nA)			
ctrl/veh	ctrl/4AP	TrkBfc/veh	TrkBfc/4AP
2.669	3.690	2.231	3.120
3.169	5.600	3.442	3.507
2.456	5.541	3.138	2.754
4.013	9.900	2.462	4.308
4.472	7.400	3.558	1.976
2.500	4.924	1.530	2.486
2.070	3.568	5.184	3.080
3.448	4.722	2.560	3.700
3.228	4.126	3.000	1.650
3.602	2.630	2.700	1.896
2.418	7.646	4.300	4.812
4.115	7.400	3.100	3.200
3.692	4.656	5.182	5.288
1.702	7.000	2.130	3.556
2.590	9.000	2.976	0.833
4.168	5.098	2.652	1.926
4.238	3.596	3.278	1.966
1.312	5.116	5.136	1.890
2.450	4.430	4.382	3.678
4.008	9.500	3.896	2.620
4.600	3.187	2.000	3.466
3.700	2.276	2.280	1.874
3.868	3.696	3.108	2.610
4.208	6.338	4.000	1.488
1.365	3.940	2.452	1.688
1.524	5.076	1.386	4.446
4.548	3.676	2.462	3.488
3.568	3.632	4.786	3.698
3.440	3.902	1.876	2.684
3.986	2.242	2.500	3.552
1.948	3.946	4.093	5.530
4.028	3.650	3.134	4.082
4.024	3.910	4.332	6.126
2.304	4.876	4.154	2.592
4.250	2.526	4.384	4.942
3.948	2.624	4.920	2.830
4.266	2.297	3.190	4.496
3.430	4.074	3.228	2.372
4.554	2.814	3.060	3.208
2.098		1.654	2.678
4.020		3.230	3.266
5.008		1.774	4.714
4.622		5.380	4.172
		3.162	
**43**	**39**	**44**	**43**
**3.3866**	**4.7237**	**3.2587**	**3.2151**
1.0093	1.9850	1.0769	1.1938
**0.1539**	**0.3179**	**0.1623**	**0.1821**

Figure 6D (right panel)

PPR (I_2/I_1)			
ctrl/veh	ctrl/4AP	TrkBfc/veh	TrkBfc/4AP
0.272	0.188	0.248	0.259
0.326	0.239	0.336	0.335
0.207	0.214	0.167	0.310
0.224	0.150	0.346	0.238
0.194	0.281	0.127	0.300
0.400	0.213	0.363	0.323
0.247	0.185	0.272	0.260
0.169	0.170	0.190	0.250
0.360	0.159	0.261	0.176
0.367	0.148	0.182	0.273
0.292	0.093	0.271	0.320
0.340	0.167	0.320	0.171
0.214	0.161	0.214	0.350
0.450	0.118	0.338	0.310
0.317	0.112	0.127	0.330
0.209	0.131	0.158	0.286
0.221	0.145	0.226	0.380
0.164	0.084	0.264	0.370
0.108	0.206	0.347	0.213
0.162	0.119	0.144	0.440
0.102	0.109	0.234	0.330
0.116	0.158	0.299	0.144
0.239	0.147	0.170	0.197
0.211	0.161	0.165	0.390
0.301	0.134	0.147	0.340
0.370	0.139	0.359	0.209
0.274	0.135	0.252	0.172
0.133	0.139	0.185	0.173
0.342	0.301	0.343	0.316
0.243	0.225	0.274	0.210
0.267	0.172	0.167	0.162
0.398	0.181	0.410	0.182
0.205	0.195	0.417	0.210
0.286	0.209	0.394	0.181
0.195	0.123	0.139	0.215
0.338	0.170	0.155	0.285
0.165	0.257	0.431	0.240
0.294	0.224	0.258	0.139
0.208	0.306	0.314	0.125
0.244		0.261	0.204
0.175		0.211	0.348
0.187		0.327	0.410
0.213		0.312	0.405
		0.317	
**43**	**39**	**44**	**43**
**0.2500**	**0.1735**	**0.2600**	**0.2670**
0.0845	0.0538	0.0866	0.0834
**0.0129**	**0.0086**	**0.0131**	**0.0127**

Figure 6

Figure 6A (upper panel)

one-way ANOVA/Dunnett’s tests

Dunnett's multiple comparisons test	Significant	Summary	P Value
0 vs. 6	Yes	*	0.0147
0 vs. 24	Yes	***	0.0003
0 vs. 48	Yes	*	0.03
0 vs. 96	No	ns	0.7404

Figure 6A (center panel)

one-way ANOVA/Dunnett’s tests

Dunnett's multiple comparisons test	Significant	Summary	P Value
0 vs. 6	Yes	****	<0,0001
0 vs. 24	Yes	*	0.0329
0 vs. 48	No	ns	0.7324
0 vs. 96	No	ns	0.3877

Figure 6A (lower panel)

one-way ANOVA/Dunnett’s tests

Dunnett's multiple comparisons test	Significant	Summary	P Value
0 vs. 6	Yes	****	<0,0001
0 vs. 24	Yes	**	0.0031
0 vs. 48	No	ns	0.9809
0 vs. 96	No	ns	0.9276

Figure 6B (upper panel)

two-way ANOVA/Tukey's tests

Tukey's multiple comparisons test	Significant	Summary	P Value
**Ctrl :scrRNA vs. 4AP 24h:scrRNA**	**Yes**	*******	**0.0006**
**Ctrl :scrRNA vs. 4AP 48h:scrRNA**	**Yes**	*****	**0.019**
Ctrl :scrRNA vs. 4AP 48h:shRNA	No	ns	0.0954
Ctrl :shRNA vs. 4AP 24h:scrRNA	Yes	****	<0,0001
Ctrl :shRNA vs. 4AP 24h:shRNA	No	ns	0.9489
Ctrl :shRNA vs. 4AP 48h:scrRNA	Yes	****	<0,0001
Ctrl :shRNA vs. 4AP 48h:shRNA	No	ns	0.9489
**4AP 24h:scrRNA vs. 4AP 24h:shRNA**	**Yes**	********	**<0,0001**
4AP 24h:scrRNA vs. 4AP 48h:scrRNA	No	ns	0.786
4AP 24h:scrRNA vs. 4AP 48h:shRNA	Yes	****	<0,0001
4AP 24h:shRNA vs. 4AP 48h:scrRNA	Yes	****	<0,0001
4AP 24h:shRNA vs. 4AP 48h:shRNA	No	ns	0.9489
**4AP 48h:scrRNA vs. 4AP 48h:shRNA**	**Yes**	********	**<0,0001**

Figure 6B (lower panel)			
**two-way ANOVA/Tukey's tests**			
Tukey's multiple comparisons test	Significant	Summary	P Value
Ctrl:scrRNA vs. Ctrl:shRNA	No	ns	>0,9999
**Ctrl:scrRNA vs. 4AP 24h:scrRNA**	**Yes**	********	**<0,0001**
Ctrl:scrRNA vs. 4AP 24h:shRNA	No	ns	0.1702
**Ctrl:scrRNA vs. 4AP 48h:scrRNA**	**No**	**ns**	**0.9773**
Ctrl:scrRNA vs. 4AP 48h:shRNA	Yes	**	0.0097
Ctrl:shRNA vs. 4AP 24h:scrRNA	Yes	****	<0,0001
Ctrl:shRNA vs. 4AP 24h:shRNA	No	ns	0.1686
Ctrl:shRNA vs. 4AP 48h:scrRNA	No	ns	0.9773
Ctrl:shRNA vs. 4AP 48h:shRNA	Yes	**	0.0079
**4AP 24h:scrRNA vs. 4AP 24h:shRNA**	**Yes**	*****	**0.035**
4AP 24h:scrRNA vs. 4AP 48h:scrRNA	Yes	***	0.0001
4AP 24h:scrRNA vs. 4AP 48h:shRNA	No	ns	0.2992
4AP 24h:shRNA vs. 4AP 48h:scrRNA	No	ns	0.2992
4AP 24h:shRNA vs. 4AP 48h:shRNA	No	ns	0.6651
**4AP 48h:scrRNA vs. 4AP 48h:shRNA**	**Yes**	*****	**0.0254**

Figure 6D (left panel)			
**two-way ANOVA/Tukey's tests**			
Tukey's multiple comparisons test	Significant	Summary	P Value
**NEG:veh vs. NEG:4AP**	**Yes**	********	**<0,0001**
NEG:veh vs. ODN:veh	No	ns	0.9895
NEG:veh vs. ODN:4AP	No	ns	0.9004
NEG:4AP vs. ODN:veh	Yes	****	<0,0001
NEG:4AP vs. ODN:4AP	Yes	****	<0,0001
ODN:veh vs. ODN:4AP	No	ns	0.9771

Figure 6D (right panel)			
**two-way ANOVA/Tukey's tests**			
Tukey's multiple comparisons test	Significant	Summary	P Value
NEG:Ctrl vs. NEG:4AP	Yes	****	<0,0001
NEG:Ctrl vs. ODN:Ctrl	No	ns	0.8053
NEG:Ctrl vs. ODN:4AP	No	ns	0.615
NEG:4AP vs. ODN:Ctrl	Yes	****	<0,0001
NEG:4AP vs. ODN:4AP	Yes	****	<0,0001
ODN:Ctrl vs. ODN:4AP	No	ns	0.9816
