# Supplementary material for: REST/NRSF drives homeostatic plasticity of inhibitory synapses in a target-dependent fashion
Source: eLife. 2021 Dec 2;10:e69058. doi: 10.7554/eLife.69058 (PMC8639147; doi:10.7554/eLife.69058)
Supplement: Figure 7—source data 1. [file elife-69058-fig7-data1.pdf]

Figure 7

Figure 7B

Somatic Density N/µm^2

ctrl/veh	ctrl/4AP	TrkBfc/veh	TrkBfc/4AP
0.019	0.062	0.018	0.009
0.017	0.035	0.007	0.034
0.020	0.044	0.025	0.028
0.016	0.032	0.015	0.018
0.009	0.019	0.035	0.033
0.015	0.062	0.042	0.030
0.036	0.042	0.030	0.014
0.059	0.038	0.030	0.010
0.024	0.051	0.043	0.016
0.015	0.068	0.031	0.009
0.026	0.080	0.013	0.005
0.021	0.058	0.013	0.025
0.010	0.081	0.011	0.020
0.009	0.083	0.052	0.015
0.017	0.080	0.046	0.014
0.008	0.048	0.005	0.034
0.029	0.041	0.022	0.022
0.012	0.064	0.035	0.028
0.033	0.056	0.043	0.021
0.044	0.045	0.015	0.026
0.025	0.036	0.016	0.036
0.048	0.050	0.024	0.016
0.033	0.062	0.016	0.027
0.046	0.044	0.019	0.014
0.037	0.034	0.014	0.017
0.019	0.048	0.038	0.024
0.019	0.062	0.051	0.015
0.039	0.077	0.020	0.030
0.019	0.053	0.015	0.041
0.050	0.077	0.023	0.022
0.044	0.073	0.024	0.022
0.004	0.070	0.030	0.037
0.025	0.044	0.019	0.040
0.030	0.047		0.034
0.040			0.013
			0.013
			0.025
			0.013
			0.058
			0.046
			0.025
			0.030
			0.036
			0.045

	ctrl/veh	ctrl/4AP	TrkBfc/veh	TrkBfc/4AP
**N**	**35**	**34**	**33**	**44**
**Media**	**0.0262**	**0.0548**	**0.0254**	**0.0247**
SD	0.0138	0.0166	0.0128	0.0116
**SE**	**0.0023**	**0.0028**	**0.0022**	**0.0017**

Figure 7

Figure 7B

two-way ANOVA/Tukey's tests

Tukey's multiple comp	Significant	Summary	P Value
Ctrl :veh vs. Ctrl :4AP	Yes	****	<0,0001
Ctrl :veh vs. Trkb-fc:ve	No	ns	0.995
Ctrl :veh vs. Trkb-fc:4/	No	ns	0.964
Ctrl :4AP vs. Trkb-fc:v	Yes	****	<0,0001
Ctrl :4AP vs. Trkb-fc:4	Yes	****	<0,0001
Trkb-fc:veh vs. Trkb-fc	No	ns	0.9965
