# Supplementary material for: REST/NRSF drives homeostatic plasticity of inhibitory synapses in a target-dependent fashion
Source: eLife. 2021 Dec 2;10:e69058. doi: 10.7554/eLife.69058 (PMC8639147; doi:10.7554/eLife.69058)
Supplement: Figure 7—figure supplement 1—source data 1. [file elife-69058-fig7-figsupp1-data1.pdf]

Figure 7 - figure supplement 1

Figure 7 - figure supplement 1B

Dendritic Density N/µm

ctrl/veh	ctrl/4AP	TrkBfc/veh	TrkBfc/4AP
0.179	0.207	0.178	0.081
0.165	0.182	0.258	0.182
0.198	0.169	0.158	0.126
0.213	0.098	0.154	0.191
0.332	0.100	0.242	0.147
0.213	0.111	0.160	0.127
0.167	0.060	0.185	0.252
0.198	0.091	0.185	0.177
0.163	0.231	0.160	0.120
0.205	0.094	0.262	0.170
0.357	0.072	0.235	0.165
0.274	0.129	0.162	0.185
0.246	0.173	0.194	0.147
0.352	0.107	0.128	0.142
0.183	0.119	0.122	0.123
0.138	0.058	0.166	0.117
0.098	0.108	0.155	0.183
0.156	0.185	0.142	0.131
0.264	0.118	0.208	0.201
0.123	0.133	0.327	0.097
0.117	0.139	0.182	0.231
0.160	0.240	0.142	0.151
0.136	0.176	0.273	0.234
0.222	0.046	0.220	0.285
0.179	0.193	0.273	0.200
0.185	0.162	0.360	0.218
0.130	0.153	0.257	0.197
0.262	0.116	0.103	0.230
0.180	0.053	0.132	0.173
0.268	0.094	0.252	0.153
0.175	0.133	0.121	0.121
0.159	0.116	0.324	0.167
0.201	0.182	0.099	0.198
0.285	0.220	0.188	0.131
0.159	0.030	0.147	0.059
0.211	0.040	0.215	0.179
0.175	0.055	0.256	0.163
0.147	0.043	0.071	0.184
0.127	0.151	0.249	0.134
0.170	0.176	0.245	0.097
0.185	0.135	0.326	0.112
0.210	0.148	0.165	0.044
0.170	0.234	0.314	0.208
0.137	0.238	0.233	0.066
0.277	0.207	0.064	0.130
0.188	0.180	0.215	0.089
0.213	0.275	0.166	0.096
0.156	0.211	0.195	0.100
0.268	0.166	0.118	0.120
0.101	0.212	0.206	0.114
0.181	0.272	0.167	0.140
0.201	0.164	0.183	0.057
0.080	0.248	0.161	0.217
0.210	0.136	0.083	0.133
0.226	0.226	0.188	0.192
0.107	0.254	0.287	0.119
0.133	0.169	0.137	0.174
0.216	0.176	0.188	0.132
0.238	0.164	0.080	0.100
0.155	0.190	0.101	0.130
0.150	0.163	0.110	0.118
0.154	0.035	0.188	0.154
0.147	0.148	0.207	0.068
	0.147	0.144	0.317
**63**	**64**	**64**	**64**
**0.1901**	**0.1494**	**0.1893**	**0.1516**
0.0587	0.0625	0.0676	0.0544
**0.0074**	**0.0078**	**0.0084**	**0.0068**

Figure 7 - figure supplement 1

Figure 7 - figure supplement 1B

two-way ANOVA/Tukey's tests

Tukey's multiple comparison	Significant	Summary	P Value
Ctrl :veh vs. Ctrl :4AP	Yes	**	0.0012
Ctrl :veh vs. Trkb-fc:veh	No	ns	0.9999
Ctrl :veh vs. Trkb-fc:4AP	Yes	**	0.0025
Ctrl :4AP vs. Trkb-fc:veh	Yes	**	0.0015
Ctrl :4AP vs. Trkb-fc:4AP	No	ns	0.997
Trkb-fc:veh vs. Trkb-fc:4AP	Yes	**	0.0031
