# Supplementary material for: REST/NRSF drives homeostatic plasticity of inhibitory synapses in a target-dependent fashion
Source: eLife. 2021 Dec 2;10:e69058. doi: 10.7554/eLife.69058 (PMC8639147; doi:10.7554/eLife.69058)
Supplement: Figure 7—figure supplement 2—source data 1. [file elife-69058-fig7-figsupp2-data1.pdf]

Figure 7-figure supplement 2

Figure7-fig suppl 2B

Somatic Density N/µm^2

	ctrl/veh	ctrl/4AP	TrkBfc/veh	TrkBfc/4AP
	0.016	0.013	0.018	0.029
	0.037	0.011	0.014	0.018
	0.036	0.022	0.035	0.029
	0.035	0.019	0.016	0.017
	0.030	0.016	0.024	0.016
	0.035	0.026	0.021	0.011
	0.023	0.046	0.029	0.035
	0.026	0.022	0.026	0.021
	0.050	0.026	0.020	0.017
	0.032	0.047	0.034	0.041
	0.019	0.038	0.030	0.015
	0.048	0.031	0.036	0.024
	0.022	0.044	0.014	0.026
	0.021	0.039	0.041	0.031
	0.023	0.020	0.023	0.019
	0.013	0.018	0.018	0.033
	0.012	0.014	0.036	0.042
	0.014	0.035	0.028	0.026
	0.033	0.016	0.023	0.016
	0.014	0.024	0.018	0.037
	0.022	0.021	0.029	0.036
	0.019	0.029	0.027	0.035
	0.016	0.026	0.016	0.030
	0.026	0.020	0.018	0.033
	0.034	0.034	0.034	0.024
	0.022	0.030	0.025	0.023
	0.013	0.036	0.018	0.041
	0.043	0.014	0.043	0.036
	0.034	0.041	0.019	0.020
	0.036	0.023	0.025	0.045
	0.040	0.018	0.026	0.023
	0.034	0.036	0.037	0.025
	0.020	0.030	0.015	0.023
			0.036	0.037
			0.040	0.018
			0.024	0.012
**N**	**33**	**33**	**36**	**36**
**Media**	**0.0271**	**0.0267**	**0.0260**	**0.0268**
SD	0.0105	0.0102	0.0084	0.0092
**SE**	**0.0018**	**0.0018**	**0.0014**	**0.0015**

Figure7-fig suppl 2C

Dendritic Density N/µm

	ctrl/veh	ctrl/4AP	TrkBfc/veh	TrkBfc/4AP
	0.314	0.137	0.176	0.123
	0.294	0.217	0.201	0.300
	0.200	0.273	0.214	0.127
	0.294	0.193	0.201	0.096
	0.250	0.199	0.200	0.352
	0.364	0.143	0.243	0.193
	0.219	0.174	0.146	0.195
	0.252	0.242	0.320	0.144
	0.255	0.139	0.195	0.119
	0.098	0.160	0.211	0.143
	0.194	0.196	0.164	0.200
	0.129	0.266	0.308	0.193
	0.260	0.203	0.123	0.229
	0.263	0.222	0.103	0.380
	0.222	0.241	0.138	0.137
	0.313	0.107	0.216	0.215
	0.277	0.279	0.148	0.308
	0.293	0.275	0.163	0.362
	0.211	0.346	0.231	0.300
	0.235	0.279	0.198	0.401
	0.198	0.278	0.159	0.401
	0.169	0.184	0.106	0.199
	0.211	0.228	0.152	0.140
	0.194	0.195	0.332	0.187
	0.180	0.207	0.270	0.328
	0.165	0.155	0.220	0.273
	0.231	0.157	0.351	0.207
	0.208	0.290	0.462	0.268
	0.149	0.214	0.419	0.340
	0.233	0.208	0.188	0.120
	0.231	0.243	0.312	0.195
	0.255	0.181	0.185	0.101
	0.126	0.174	0.233	0.101
	0.099	0.373	0.109	0.134
	0.196	0.234	0.297	0.093
	0.147	0.202	0.349	0.135
	0.126	0.177	0.229	0.072
	0.293	0.197	0.173	0.127
	0.157	0.128	0.308	0.140
	0.313	0.188	0.135	0.186
	0.277	0.177	0.129	0.180
	0.189	0.229	0.155	0.168
	0.239	0.226	0.074	0.298
	0.178	0.208	0.165	0.122
	0.170	0.354	0.217	0.152
	0.220	0.167	0.084	0.134
	0.190	0.204	0.123	0.114
	0.298	0.182	0.186	0.195
	0.246	0.123	0.179	0.161
	0.165	0.220	0.143	0.146
	0.075	0.263	0.130	0.118
	0.227	0.240	0.112	0.369
	0.219	0.162	0.129	0.292
		0.188	0.192	0.271
		0.119	0.119	0.189
			0.235	0.134
			0.198	0.172
			0.159	0.200
			0.213	0.379
			0.257	0.115
			0.205	0.155
			0.183	0.154
			0.190	0.124
			0.212	0.132
			0.111	0.154
			0.169	0.166
			0.132	0.299
			0.155	0.398
			0.162	0.109
			0.119	0.328
			0.099	0.127
			0.156	0.207
			0.130	
			0.225	
			0.180	
			0.163	
			0.213	
			0.124	
			0.142	
			0.182	
			0.185	
			0.134	
			0.154	
			0.140	
			0.189	
**N**	**53**	**55**	**85**	**72**
**Media**	**0.2172**	**0.2103**	**0.1898**	**0.2018**
SD	0.0621	0.0571	0.0721	0.0905
**SE**	**0.0085**	**0.0077**	**0.0078**	**0.0107**

Figure 7-figure supplement 2

Figure7-fig suppl 2B

two-way ANOVA/Tukey's tests

Tukey's multiple comparisons t	Significant	Summary	P Value
Ctrl :veh vs. Ctrl :4AP	No	ns	0.9981
Ctrl :veh vs. Trkb-fc:veh	No	ns	0.9586
Ctrl :veh vs. Trkb-fc:4AP	No	ns	0.9993
Ctrl :4AP vs. Trkb-fc:veh	No	ns	0.9881
Ctrl :4AP vs. Trkb-fc:4AP	No	ns	>0,9999
Trkb-fc:veh vs. Trkb-fc:4AP	No	ns	0.9805

Figure7-fig suppl 2C

two-way ANOVA/Tukey's tests

Tukey's multiple comparisons t	Significant	Summary	P Value
Ctrl :veh vs. Ctrl :4AP	No	ns	0.9612
Ctrl :veh vs. Trk-fc:veh	No	ns	0.1435
Ctrl :veh vs. Trk-fc:4AP	No	ns	0.6484
Ctrl :4AP vs. Trk-fc:veh	No	ns	0.3701
Ctrl :4AP vs. Trk-fc:4AP	No	ns	0.9147
Trk-fc:veh vs. Trk-fc:4AP	No	ns	0.7383
