# Supplementary material for: REST/NRSF drives homeostatic plasticity of inhibitory synapses in a target-dependent fashion
Source: eLife. 2021 Dec 2;10:e69058. doi: 10.7554/eLife.69058 (PMC8639147; doi:10.7554/eLife.69058)
Supplement: Figure 7—figure supplement 3—source data 1. [file elife-69058-fig7-figsupp3-data1.pdf]

Figure 7-figure supplement 3

Figure 7-figure supplement 3B

Somatic Density N/μm^2

	NEG/veh(TrkBfc)/veh(4AP)	NEG/veh(TrkBfc)/4AP	ODN/veh(TrkBfc)/4AP	NEG/TrkBfc/4AP	ODN/TrkBfc/4AP	4AP/NEG/BDNF	4AP/ODN/BDNF
	0.0383	0.0589	0.0385	0.0699	0.0865	0.0661	0.0603
	0.0289	0.0723	0.0462	0.0360	0.0419	0.0512	0.0804
	0.0547	0.0616	0.0496	0.0433	0.0302	0.0696	0.0442
	0.0474	0.0671	0.0386	0.0394	0.0281	0.1066	0.0870
	0.0627	0.0497	0.0310	0.0330	0.0368	0.0551	0.0777
	0.0402	0.0524	0.0289	0.0358	0.0265	0.0828	0.0933
	0.0428	0.0702	0.0456	0.0516	0.0516	0.0822	0.1054
	0.0483	0.0635	0.0395	0.0408	0.0418	0.0591	0.0716
	0.0536	0.0670	0.0495	0.0516	0.0467	0.0629	0.0741
	0.0537	0.0492	0.0524	0.0637	0.0418	0.0803	0.0721
	0.0337	0.0535	0.0530	0.0725	0.0628	0.1109	0.0858
	0.0481	0.0521	0.0489	0.0431	0.0393	0.0638	0.0650
	0.0552	0.0684	0.0402	0.0526	0.0482	0.0737	0.0760
	0.0593	0.1175	0.0357	0.0470	0.0482	0.0695	0.0794
	0.0446	0.0947	0.0381	0.0404	0.0454	0.0634	0.0522
	0.0764	0.1001	0.0750	0.0412	0.0359	0.0661	0.0781
	0.0607	0.1126	0.0782	0.0414	0.0496	0.0698	0.0608
	0.0627	0.0910	0.0486	0.0409	0.0423	0.0577	0.0913
	0.0559	0.0873	0.0387	0.0222	0.0633	0.0810	0.0802
	0.0673	0.0804	0.0373	0.0497	0.0601	0.0657	0.0491
	0.0261	0.0820	0.0340	0.0432	0.0410	0.0617	0.0737
	0.0336	0.0686	0.0411	0.0575	0.0516	0.0981	0.0863
	0.0475	0.0965	0.0563	0.0768	0.0270	0.0700	0.0870
	0.0788	0.0971	0.0418	0.0658	0.0374	0.0989	0.0691
	0.0740	0.0864	0.0521	0.0290	0.0306	0.0644	0.0748
	0.0713	0.0811	0.0320	0.0258	0.0276	0.0468	0.0955
	0.0625	0.0436	0.0435	0.0557	0.0662	0.0751	0.0807
	0.0417	0.0790	0.0511	0.0660	0.0501	0.0720	0.0545
	0.0443	0.0716	0.0510	0.0661	0.0511	0.0682	0.1027
	0.0587	0.1079	0.0514	0.0571	0.0542	0.0566	0.0629
			0.0672	0.0496	0.0380		
			0.0348				
			0.0281				
			0.0387				
			0.0262				
**N**	30	30	35	31	31	30	30
**Media**	0.0524	0.0761	0.0446	0.0487	0.0452	0.0717	0.0757
SD	0.0138	0.0199	0.0120	0.0139	0.0134	0.0155	0.0151
**SE**	0.0025	0.0036	0.0020	0.0025	0.0024	0.0028	0.0028

Figure 7-figure supplement 3

Figure7-fig suppl 3B

**Two-way ANOVA**	Ordinary				
Alpha	0.05				
Source of Variation	% of total variatio	P value	P value summa	Significant?	
Interaction	12.79	<0.0001	****	Yes	
Row Factor	11.78	<0.0001	****	Yes	
Column Factor	19.87	<0.0001	****	Yes	
ANOVA table	SS (Type III)	DF	MS	F (DFn, DFd	P value
Interaction	0.006206	1	0.006206	F (1, 123) = 2	P<0.0001
Row Factor	0.005713	1	0.005713	F (1, 123) = 2	P<0.0001
Column Factor	0.009638	1	0.009638	F (1, 123) = 4	P<0.0001
Residual	0.02759	123	0.0002243		

**two-way ANOVA/Tukey's tests**	Significant	Summary	Adjusted P Value
veh(TrkBfc):NEG 4AP vs. veh(TrkBfc):ODN 4AP	Yes	****	<0.0001
veh(TrkBfc):NEG 4AP vs. TrkBfc:NEG 4AP	Yes	****	<0.0001
veh(TrkBfc):NEG 4AP vs. TrkBfc:ODN 4AP	Yes	****	<0.0001
veh(TrkBfc):ODN 4AP vs. TrkBfc:NEG 4AP	No	ns	0.698
veh(TrkBfc):ODN 4AP vs. TrkBfc:ODN 4AP	No	ns	0.9987
TrkBfc:NEG 4AP vs. TrkBfc:ODN 4AP	No	ns	0.8015

Figure7-fig suppl 3B

Two-way ANOVA	Ordinary				
Alpha	0.05				
Source of Variation	% of total variatio	P value	P value summa	Significant?	
Interaction	18.57	<0.0001	****	Yes	
Row Factor	10.41	<0.0001	****	Yes	
Column Factor	11.05	<0.0001	****	Yes	
ANOVA table	SS (Type III)	DF	MS	F (DFn, DFd	P value
Interaction	0.009814	1	0.009814	F (1, 121) = 3	P<0.0001
Row Factor	0.005499	1	0.005499	F (1, 121) = 2	P<0.0001
Column Factor	0.005837	1	0.005837	F (1, 121) = 2	P<0.0001
Residual	0.03004	121	0.0002483		

**two-way ANOVA/Tukey's tests**	Significant	Summary	Adjusted P Value
veh(TrkBfc):NEG 4AP vs. veh(TrkBfc):ODN 4AP	Yes	****	<0.0001
veh(TrkBfc):NEG 4AP vs. BDNF:NEG 4AP	No	ns	0.6915
veh(TrkBfc):NEG 4AP vs. BDNF:ODN 4AP	No	ns	0.9996
veh(TrkBfc):ODN 4AP vs. BDNF:NEG 4AP	Yes	****	<0.0001
veh(TrkBfc):ODN 4AP vs. BDNF:ODN 4AP	Yes	****	<0.0001
BDNF:NEG 4AP vs. BDNF:ODN 4AP	No	ns	0.7504
