# Supplementary material for: REST/NRSF drives homeostatic plasticity of inhibitory synapses in a target-dependent fashion
Source: eLife. 2021 Dec 2;10:e69058. doi: 10.7554/eLife.69058 (PMC8639147; doi:10.7554/eLife.69058)
Supplement: Figure 8—source data 1. [file elife-69058-fig8-data1.pdf]

Figure 8

Figure 8A

	NEG/veh						NEG/4AP						ODN/veh						ODN/4AP					
	0hrs	1hrs	3hrs	6hrs	12hrs	24hrs	0hrs	1hrs	3hrs	6hrs	12hrs	24hrs	0hrs	1hrs	3hrs	6hrs	12hrs	24hrs	0hrs	1hrs	3hrs	6hrs	12hrs	24hrs
	1.254	1.254	0.381	1.476	0.411	0.615	1.254	0.898	2.549	1.851	2.720	1.228	1.254	1.158	2.520	1.840	3.099	0.602	1.254	0.813	1.760	1.714	1.768	1.376
	1.032	1.032	1.113	1.220	0.901	1.713	1.032	0.596	1.516	1.841	1.543	4.343	1.032	0.482	0.810	1.048	1.433	1.468	1.032	1.528	2.517	3.913	1.712	1.686
	0.713	0.713	1.507	0.304	1.090	0.672	0.713	0.388	2.804	0.916	2.271	1.275	0.713	0.956	2.434	1.766	1.384	0.469	0.713	1.741	1.848	2.980	0.783	2.307
	0.772	0.772	1.154	0.671	0.910	1.000	0.772	3.986	2.030	6.051	3.217	1.820	0.772	1.721	0.705	2.950	0.961	0.363	0.772	2.168	2.021	3.407	4.617	0.979
	1.102	1.102	0.727	1.829	1.031	1.017	1.102	3.145	1.349	4.814	1.923	2.948	1.102	6.511	5.185	3.239	0.629	0.453	1.102	4.604	1.533	3.075	3.000	2.017
	1.126	1.126	1.119	0.500	1.094	0.983	1.126	3.487	2.000	1.379	1.515	2.905	1.126	1.795	4.174	1.202	2.554	0.267	1.126	5.254	1.948	1.011		2.395
	1.567	1.567	1.249	1.501	0.875	0.478	1.567	2.994	2.700	2.479	2.908	1.771	1.567	3.992	0.554	1.120	0.893	4.351	1.567	1.287	1.564	0.802		2.900
	0.433	0.433	0.751	0.499	1.000	0.581	0.433	5.234	3.000	0.704	2.700		0.433	1.056	1.114	0.807	1.284	0.590	0.433	1.052		1.452		
				0.868		1.000		2.764		1.066					0.559	0.152				1.351		1.643		
				1.210						2.248						1.024						2.724		
				0.921						3.588						1.345						2.801		
																1.559								
**N**	**8**	**8**	**8**	**11**	**8**	**9**	**8**	**9**	**8**	**11**	**8**	**7**	**8**	**8**	**9**	**12**	**8**	**8**	**8**	**9**	**7**	**11**	**5**	**7**
**Media**	**1.0000**	**1.0000**	**1.0000**	**1.0000**	**0.9140**	**0.8954**	**1.0000**	**2.6103**	**2.2436**	**2.4489**	**2.3495**	**2.3272**	**1.0000**	**2.2088**	**2.0061**	**1.5044**	**1.5295**	**1.0704**	**1.0000**	**2.1998**	**1.8847**	**2.3201**	**2.3761**	**1.9513**
SD	0.3522	0.3522	0.3566	0.4875	0.2200	0.3732	0.3522	1.6557	0.6120	1.7055	0.6392	1.1302	0.3522	2.0383	1.7093	0.8686	0.8571	1.3765	0.3522	1.6035	0.3330	1.0353	1.4796	0.6551
**SE**	**0.1245**	**0.1245**	**0.1261**	**0.1470**	**0.0778**	**0.1244**	**0.1245**	**0.5519**	**0.2164**	**0.5142**	**0.2260**	**0.4272**	**0.1245**	**0.7206**	**0.5698**	**0.2507**	**0.3030**	**0.4867**	**0.1245**	**0.5345**	**0.1259**	**0.3122**	**0.6617**	**0.2476**

Figure 8B

	NEG/veh						NEG/4AP						ODN/veh						ODN/4AP					
	0hrs	1hrs	3hrs	6hrs	12hrs	24hrs	0hrs	1hrs	3hrs	6hrs	12hrs	24hrs	0hrs	1hrs	3hrs	6hrs	12hrs	24hrs	0hrs	1hrs	3hrs	6hrs	12hrs	24hrs
	1.100	1.100	0.703	0.623	1.558	0.654	1.100	3.103	0.351	3.966	2.625	4.456	1.100	1.090	0.978	0.750	1.046	1.000	1.100	2.982	0.276	1.348	1.935	2.453
	1.032	1.032	0.769	0.837	0.200	1.129	1.032	4.802	5.225	2.693	1.000	1.000	1.032	0.646	0.996	0.589	1.443	1.000	1.032	0.236	1.000	1.619	2.760	6.096
	0.868	0.868	1.527	1.540	1.242	1.217	0.868	5.317	5.763	2.846	2.996	1.000	0.868	0.498	0.757	0.915	1.206	1.044	0.868	2.191	0.281	2.023	1.153	1.973
	1.265	1.265	1.273	1.194	0.677	1.144	1.265	1.973	3.315	3.472	0.348	0.364	1.265	1.460	0.489	2.001	1.800	0.556	1.265	0.375	1.000	6.284	5.000	1.000
	0.473	0.473	1.144	1.328	1.323	0.633	0.473	1.732	2.320	7.708	2.814	0.460	0.473	1.022	0.886	0.397	0.634	0.749	0.473	0.434	6.472	9.539	1.009	1.000
	1.262	1.262	0.583	0.478	1.216	1.223	1.262	2.000	0.603	3.978	2.343	0.244	1.262	0.793	4.728	0.345	0.616	0.948	1.262	0.612	1.000	1.648	4.000	6.124
	0.786	0.786	1.759	1.170	0.733	1.130	0.786	1.010	3.989	3.262	4.654	0.920	0.786	1.495	0.370	2.413	1.470	2.700	0.786	0.839	3.002	4.770	5.000	8.487
	1.531	1.531	0.772	0.830	1.051	0.920	1.531		1.006	5.079	2.018	1.450	1.531	2.080	0.495	0.754	0.745	1.000	1.531	0.943	0.056	3.495		5.675
	0.682	0.682	0.469			0.950	0.682					2.674	0.682	1.107	0.347	1.752			0.682	0.599	5.330	3.839		
**N**	**9**	**9**	**9**	**8**	**8**	**9**	**9**	**7**	**8**	**8**	**8**	**9**	**9**	**9**	**9**	**9**	**8**	**8**	**9**	**9**	**9**	**9**	**7**	**8**
**Media**	**1.0000**	**1.0000**	**1.0000**	**1.0000**	**1.0000**	**1.0000**	**1.0000**	**2.8481**	**2.8215**	**4.1253**	**2.3497**	**1.3965**	**1.0000**	**1.1324**	**1.1163**	**1.1015**	**1.1199**	**1.1248**	**1.0000**	**1.0233**	**2.0465**	**3.8407**	**2.9795**	**4.1011**
SD	0.3307	0.3307	0.4472	0.3656	0.4374	0.2274	0.3307	1.6373	2.0909	1.6322	1.3071	1.3606	0.3307	0.4882	1.3776	0.7553	0.4364	0.6579	0.3307	0.9337	2.3660	2.7086	1.7111	2.8351
**SE**	**0.1102**	**0.1102**	**0.1491**	**0.1293**	**0.1546**	**0.0758**	**0.1102**	**0.6188**	**0.7392**	**0.5771**	**0.4621**	**0.4535**	**0.1102**	**0.1627**	**0.4592**	**0.2518**	**0.1543**	**0.2326**	**0.1102**	**0.3112**	**0.7887**	**0.9029**	**0.6467**	**1.0024**

Figure 8C

	NEG/veh						NEG/4AP						ODN/veh						ODN/4AP					
	0hrs	1hrs	3hrs	6hrs	12hrs	24hrs	0hrs	1hrs	3hrs	6hrs	12hrs	24hrs	0hrs	1hrs	3hrs	6hrs	12hrs	24hrs	0hrs	1hrs	3hrs	6hrs	12hrs	24hrs
	1.111	1.111	0.643	0.896	0.957	0.537	1.111	1.459	1.780	2.243	2.107	3.087	1.111	1.288	0.738	1.098	1.007	1.000	1.111	0.765	0.491	0.709	2.526	1.713
	1.045	1.045	0.646	1.231	1.117	1.165	1.045	1.162	1.670	0.931	2.462	4.164	1.045	0.868	0.753	0.978	1.248	0.500	1.045	0.601	1.723	1.111	2.093	2.697
	0.844	0.844	1.711	0.873	0.925	1.299	0.844	0.706	1.880	2.009	2.462	1.428	0.844	0.867	0.690	0.962	1.056	0.600	0.844	0.947	0.567	0.787	2.000	1.209
	1.246	1.246	0.965	1.108	0.633	0.924	1.246	1.541	0.929	1.358	3.350	1.670	1.246	1.186	1.327	1.739	1.117	1.126	1.246	1.219	1.812	2.497	1.344	1.000
	0.676	0.676	0.943	0.681	1.367	0.645	0.676	0.782	0.877	2.722	1.680	1.990	0.676	1.385	1.556	1.280	1.034	0.537	0.676	0.787	1.005	2.458	1.905	1.420
	1.077	1.077	1.091	1.211	1.189	1.431	1.077	1.230	1.157	3.215	1.824	2.023	1.077	0.876	1.397	0.427	0.994	1.873	1.077	0.818	1.706	1.243	1.814	1.710
	0.893	0.893	1.377	0.993	0.831	0.959	0.893	1.057	1.420	2.163	2.370	1.970	0.893	1.505	0.497	1.459	0.808	1.528	0.893	1.001	0.883	2.893	1.000	1.450
	1.228	1.228	0.989	1.007	0.980	1.085	1.228	0.959	1.800	3.708	2.567	1.782	1.228	1.326	0.727	0.870	0.759	1.328	1.228	1.207	0.644	1.300	1.000	3.091
	0.880	0.880	0.634			0.956	0.880	0.980				3.059	0.880	0.974	0.617	0.872			0.880	0.982	1.952	2.000		2.793
																								0.500
**N**	**9**	**9**	**9**	**8**	**8**	**9**	**9**	**9**	**8**	**8**	**8**	**9**	**9**	**9**	**9**	**9**	**8**	**8**	**9**	**9**	**9**	**9**	**8**	**10**
**Media**	**1.0000**	**1.0000**	**1.0000**	**1.0000**	**1.0000**	**1.0000**	**1.0000**	**1.0972**	**1.4392**	**2.2935**	**2.3527**	**2.3526**	**1.0000**	**1.1416**	**0.9225**	**1.0762**	**1.0029**	**1.0616**	**1.0000**	**0.9251**	**1.1982**	**1.6664**	**1.7104**	**1.7583**
SD	0.1898	0.1898	0.3617	0.1845	0.2251	0.2870	0.1898	0.2822	0.4053	0.9155	0.5150	0.8911	0.1898	0.2497	0.3905	0.3800	0.1579	0.5012	0.1898	0.2051	0.5939	0.8096	0.5462	0.8434
**SE**	**0.0633**	**0.0633**	**0.1206**	**0.0652**	**0.0796**	**0.0957**	**0.0633**	**0.0941**	**0.1433**	**0.3237**	**0.1821**	**0.2970**	**0.0633**	**0.0832**	**0.1302**	**0.1267**	**0.0558**	**0.1772**	**0.0633**	**0.0684**	**0.1980**	**0.2699**	**0.1931**	**0.2667**

Figure 8D

	NEG/veh						NEG/4AP						ODN/veh						ODN/4AP					
	0hrs	1hrs	3hrs	6hrs	12hrs	24hrs	0hrs	1hrs	3hrs	6hrs	12hrs	24hrs	0hrs	1hrs	3hrs	6hrs	12hrs	24hrs	0hrs	1hrs	3hrs	6hrs	12hrs	24hrs
	0.638	0.638	0.866	1.005	1.086	0.807	0.638	0.841	0.904	1.764	2.018	2.417	0.638	1.819	0.807	0.939	1.088	1.000	0.638	0.904	0.713	1.660	1.773	1.596
	1.141	1.141	0.757	1.172	1.010	0.976	1.141	0.813	0.949	2.000	2.737	3.000	1.141	1.083	1.038	0.978	1.673	1.424	1.141	0.683	1.129	0.978	1.749	3.000
	1.221	1.221	1.377	0.823	0.904	1.216	1.221	0.910	1.077	1.127	2.133	1.499	1.221	1.202	0.915	0.919	0.764	1.000	1.221	1.072	0.737	1.990	1.689	1.749
	0.988	0.988	0.858	1.009	0.759	0.971	0.988	1.732	1.122	1.163	0.991	1.893	0.988	0.942	0.885	1.648	0.851	0.704	0.988	0.914	0.973	1.404	0.903	1.628
	0.942	0.942	0.822	0.833	1.241	0.798	0.942	0.784	0.831	2.303	0.604	2.400	0.942	1.116	1.025	2.071	0.865	0.734	0.942	0.874	0.736	1.000	1.984	1.485
	1.070	1.070	1.320	1.158	1.156	1.230	1.070	0.895	0.868	2.956	2.231	2.048	1.070	1.073	1.066	0.543	0.717	1.095	1.070	1.109	0.957	0.757	2.045	2.268
	0.819	0.819	1.299	1.021	0.844	0.711	0.819	0.897	1.449	2.315	2.112	1.280	0.819	0.813	0.639	0.814	0.764	1.641	0.819	0.891	0.887	2.184	0.963	1.985
	1.361	1.361	1.052	0.979		1.039	1.361	0.984	0.764	3.000	1.733	1.988	1.361	0.813	0.748	0.545	0.937	1.165	1.361	0.967	0.618	2.045	2.000	1.644
	0.820	0.820	0.649			1.250	0.820	1.052				2.483	0.820	0.671	0.784	1.021			0.820	0.767	1.956	1.839		1.506
																								2.000
**N**	**9**	**9**	**9**	**8**	**7**	**9**	**9**	**9**	**8**	**8**	**8**	**9**	**9**	**9**	**9**	**9**	**8**	**8**	**9**	**9**	**9**	**9**	**8**	**10**
**Media**	**1.0000**	**1.0000**	**1.0000**	**1.0000**	**1.0000**	**1.0000**	**1.0000**	**0.9897**	**0.9955**	**2.0787**	**1.8197**	**2.1121**	**1.0000**	**1.0590**	**0.8786**	**1.0531**	**0.9573**	**1.0953**	**1.0000**	**0.9090**	**0.9674**	**1.5396**	**1.6383**	**1.8862**
SD	0.2245	0.2245	0.2713	0.1282	0.1739	0.2021	0.2245	0.2905	0.2190	0.7144	0.6975	0.5289	0.2245	0.3329	0.1466	0.5005	0.3121	0.3192	0.2245	0.1335	0.4035	0.5262	0.4542	0.4646
**SE**	**0.0748**	**0.0748**	**0.0904**	**0.0453**	**0.0657**	**0.0674**	**0.0748**	**0.0968**	**0.0774**	**0.2526**	**0.2466**	**0.1763**	**0.0748**	**0.1110**	**0.0489**	**0.1668**	**0.1103**	**0.1129**	**0.0748**	**0.0445**	**0.1345**	**0.1754**	**0.1606**	**0.1469**

Figure 8

Figure 8E

	NEG/veh						NEG/4AP						ODN/veh						ODN/4AP					
	0hrs	1hrs	3hrs	6hrs	12hrs	24hrs	0hrs	1hrs	3hrs	6hrs	12hrs	24hrs	0hrs	1hrs	3hrs	6hrs	12hrs	24hrs	0hrs	1hrs	3hrs	6hrs	12hrs	24hrs
	1.000	1.175	0.395	0.989	1.683	0.508	1.000	2.945	0.321	2.000	3.135	5.595	0.730	0.730	1.036	0.986	1.226	2.244	0.556	0.556	2.498	3.000	1.990	1.713
	1.086	1.086	2.039	1.444	0.600	1.449	1.000	2.063	1.906	3.092	3.450	5.546	0.532	0.532	1.000	0.846	1.085	1.000	0.253	0.253	0.328	3.000	3.358	4.236
	0.739	0.739	0.566	0.567	0.717	1.043	0.846	0.846	1.411	3.000	2.649	0.944	0.601	0.601	2.712	0.746	2.122	2.862	0.803	0.803	1.382	0.471	1.102	0.526
	1.208	1.208	0.769	0.484	2.677	1.027	0.681	0.681	0.686	5.035	3.549	3.000	1.457	1.457	2.969	0.367	0.204	1.000	1.167	1.167	2.797	1.000	1.409	1.325
	1.000	0.585	0.787	1.516	0.204	0.902	0.471	0.471	0.581	5.422	2.665	4.000	1.000	2.189	1.844	0.728	0.995	0.506	1.159	1.159	0.766	2.862	1.673	0.463
	1.207	1.207	1.444		0.119	1.071	1.492	1.492	0.504	5.609	2.177	3.000	0.882	0.882	1.287	1.345	0.103	0.945	0.833	0.833	1.493	3.880	1.221	3.992
	1.000	1.375	1.659		0.669	1.325	0.821	0.821	0.521		2.994	1.849	0.696	0.696	0.273		2.250	1.602	1.000	2.201	2.212		2.173	3.393
	0.887	0.887	0.871		1.331	0.906	0.882	0.882	1.439		3.503	2.031	0.867	0.867	0.578		1.272	0.977	1.000	2.395	0.506			
	0.738	0.738	0.470			0.769	0.784	0.784	0.685			4.517			0.395		0.516		1.000	2.072	0.259			
**N**	**9**	**9**	**9**	**5**	**8**	**9**	**9**	**9**	**9**	**6**	**8**	**9**	**8**	**8**	**9**	**6**	**9**	**8**	**9**	**9**	**9**	**6**	**7**	**7**
**Media**	**0.9850**	**1.0000**	**1.0000**	**1.0000**	**1.0000**	**1.0000**	**0.8864**	**1.2206**	**0.8949**	**4.0263**	**3.0152**	**3.3868**	**0.8457**	**0.9943**	**1.3437**	**0.8363**	**1.0860**	**1.3921**	**0.8636**	**1.2712**	**1.3600**	**2.3688**	**1.8465**	**2.2354**
SD	0.1736	0.2708	0.5769	0.4787	0.8576	0.2804	0.2791	0.8073	0.5470	1.5167	0.4909	1.6457	0.2913	0.5604	0.9755	0.3228	0.7550	0.7924	0.2972	0.7706	0.9660	1.3264	0.7725	1.6118
**SE**	**0.0579**	**0.0903**	**0.1923**	**0.2141**	**0.3032**	**0.0935**	**0.0930**	**0.2691**	**0.1823**	**0.6192**	**0.1736**	**0.5486**	**0.1030**	**0.1981**	**0.3252**	**0.1318**	**0.2517**	**0.2802**	**0.0991**	**0.2569**	**0.3220**	**0.5415**	**0.2920**	**0.6092**

Figure 8F

	NEG/veh						NEG/4AP						ODN/veh						ODN/4AP					
	0hrs	1hrs	3hrs	6hrs	12hrs	24hrs	0hrs	1hrs	3hrs	6hrs	12hrs	24hrs	0hrs	1hrs	3hrs	6hrs	12hrs	24hrs	0hrs	1hrs	3hrs	6hrs	12hrs	24hrs
	1.000	0.954	0.525	0.926	1.193	0.716	1.000	1.806	0.807	0.870	2.610	3.338	0.730	1.781	0.869	0.968	1.039	1.000	0.556	1.033	2.000	3.400	1.435	2.610
	1.086	1.102	1.547	0.903	0.922	1.082	1.000	1.909	2.587	2.839	1.130	3.946	0.532	1.046	1.812	0.804	1.207	2.879	0.253	0.667	0.705	0.922	1.226	6.375
	0.739	0.944	0.929	1.171	0.885	1.202	0.846	1.204	2.862	1.015	1.709	2.500	0.601	0.880	1.815	0.987	1.264	1.000	0.803	1.020	2.773	0.911	2.771	0.959
	1.208	1.318	1.191	0.755	2.053	1.088	0.681	1.203	1.496	3.908	3.890	0.607	1.457	1.159	1.256	0.500	0.636	1.271	1.167	0.733	3.122	2.000	2.143	2.072
	1.000	0.563	0.585	1.245	0.438	0.763	0.471	0.518	1.149	3.793	2.808	1.262	1.000	1.379	1.162	1.192	1.204	0.742	1.159	1.205	1.558	2.000	4.261	1.203
	1.207	1.119	1.224		0.509	1.149	1.492	0.827	0.952	4.667	2.076	1.119	0.882	0.822	1.830	1.499	0.528	1.120	0.833	0.679	1.388	2.000	1.511	2.473
	1.000	0.881	0.811		1.011	0.971	0.821	1.195	1.408		1.688	0.828	0.696	2.773	0.696		1.552	0.991	1.000	1.702	2.000		1.234	2.051
	0.887	1.016	1.356		0.989	1.170	0.882	1.596	3.482		1.249	1.544	0.867	0.730	1.251		1.113	1.091	1.000	2.174	1.458			1.165
	0.738	1.103	0.834			0.859	0.784	0.812	2.884			1.428		0.971	0.759		0.786		1.000	2.817	1.309			
**N**	**9**	**9**	**9**	**5**	**8**	**9**	**9**	**9**	**9**	**6**	**8**	**9**	**8**	**9**	**9**	**6**	**9**	**8**	**9**	**9**	**9**	**6**	**7**	**8**
**Media**	**0.9850**	**1.0000**	**1.0000**	**1.0000**	**1.0000**	**1.0000**	**0.8864**	**1.2300**	**1.9586**	**2.8486**	**2.1451**	**1.8414**	**0.8457**	**1.2823**	**1.2720**	**0.9917**	**1.0364**	**1.2617**	**0.8636**	**1.3367**	**1.8127**	**1.8723**	**2.0831**	**2.3633**
SD	0.1736	0.2081	0.3498	0.2027	0.4961	0.1817	0.2791	0.4707	0.9939	1.5871	0.9210	1.1604	0.2913	0.6447	0.4566	0.3390	0.3282	0.6702	0.2972	0.7485	0.7548	0.9175	1.1122	1.7361
**SE**	**0.0579**	**0.0694**	**0.1166**	**0.0906**	**0.1754**	**0.0606**	**0.0930**	**0.1569**	**0.3313**	**0.6479**	**0.3256**	**0.3868**	**0.1030**	**0.2149**	**0.1522**	**0.1384**	**0.1094**	**0.2370**	**0.0991**	**0.2495**	**0.2516**	**0.3746**	**0.4204**	**0.6138**

Figure 8G

	NEG/veh						NEG/4AP						ODN/veh						ODN/4AP					
	0hrs	1hrs	3hrs	6hrs	12hrs	24hrs	0hrs	1hrs	3hrs	6hrs	12hrs	24hrs	0hrs	1hrs	3hrs	6hrs	12hrs	24hrs	0hrs	1hrs	3hrs	6hrs	12hrs	24hrs
	1.000	0.924	0.628	0.942	1.237	0.560	0.846	1.286	0.991	1.000	1.436	2.228	1.000	1.603	0.813	0.849	1.011	1.401	1.000	0.964	2.226	0.623	1.121	1.523
	1.086	1.171	1.414	1.089	0.874	1.098	0.681	1.390	1.615	2.134	0.976	2.863	0.882	1.008	2.029	0.940	0.955	1.743	1.000	0.681	0.642	0.884	1.015	3.365
	0.739	0.905	0.958	0.969	0.889	1.342	1.000	1.114	1.792	0.760	0.994	1.525	0.696	0.935	1.799	0.841	0.965	1.000	1.000	1.044	2.452	0.888	1.116	1.376
	1.208	1.144	1.118	0.577	1.816	0.948	1.492	1.433	1.280	2.756	2.502	1.448	0.867	1.043	1.224	0.447	0.709	1.254		0.732	1.984	2.658	2.524	1.766
	1.000	0.816	0.677	1.423	0.623	0.968	0.821	0.729	1.062	2.486	2.750	1.565	0.556	1.378	1.173	0.779	1.610	0.812		0.894	1.107	1.183	1.905	1.303
	1.207	1.040	1.205		0.561	1.084	0.882	0.898	0.800	2.000	1.731	1.650	1.000	0.991	1.452	1.271	0.469	0.976		0.627	1.280	1.913	2.906	1.372
	1.000	1.106	1.380		0.827	0.950	0.784	1.223	0.873		1.579	0.913	0.803	2.357	0.534		1.298	1.138		1.494	1.877		1.425	2.241
	0.887	0.990	0.926		1.173	1.022	0.730	1.163	1.520		1.585	1.579	1.167	0.966	0.855		1.110	0.839		1.838	1.003		1.881	1.267
	0.738	0.905	0.693			1.028	1.000	0.915	1.365			1.175	1.159	1.001	0.693		0.654			1.616	0.507		1.227	
	1.000						0.601						0.833											
	1.000						1.457																	
N	11	9	9	5	8	9	11	9	9	6	8	9	10	9	9	6	9	8	3	9	9	6	9	8
Media	0.9877	1.0000	1.0000	1.0000	1.0000	1.0000	0.9359	1.1279	1.2554	1.8560	1.6941	1.6607	0.8964	1.2536	1.1746	0.8545	0.9757	1.1453	1.0000	1.0987	1.4532	1.3583	1.6800	1.7765
SD	0.1554	0.1227	0.2993	0.3042	0.4044	0.2045	0.2926	0.2384	0.3465	0.8047	0.6400	0.5746	0.1925	0.4712	0.5091	0.2655	0.3458	0.3136	0.0000	0.4422	0.7038	0.7766	0.6755	0.7181
SE	0.0468	0.0409	0.0998	0.1361	0.1430	0.0682	0.0882	0.0795	0.1155	0.3285	0.2263	0.1915	0.0609	0.1571	0.1697	0.1084	0.1153	0.1109	0.0000	0.1474	0.2346	0.3170	0.2252	0.2539

Figure 8H

	NEG/veh						NEG/4AP						ODN/veh						ODN/4AP					
	0hrs	1hrs	3hrs	6hrs	12hrs	24hrs	0hrs	1hrs	3hrs	6hrs	12hrs	24hrs	0hrs	1hrs	3hrs	6hrs	12hrs	24hrs	0hrs	1hrs	3hrs	6hrs	12hrs	24hrs
	1.000	1.319	0.992	0.863	1.053	0.891	0.846	0.533	1.159	0.832	0.832	1.772	1.000	1.643	0.819	1.002	1.191	1.088	1.000	0.939	4.052	0.535	0.877	1.000
	1.086	0.968	0.833	0.981	1.150	0.987	1.000	0.716	3.666	1.902	0.881	2.253	0.882	1.140	1.529	0.768	0.991	1.753	1.000	0.722	3.940	0.605	0.741	0.400
	0.739	0.713	1.175	1.156	0.797	1.121	0.471	0.671	3.144	1.708	1.016	1.396	0.696	0.891	1.054	0.806	0.932	1.000	1.000	1.055	1.252	0.551	0.908	0.371
	1.208	1.276	1.094	0.976	1.627	1.014	1.492	1.784	1.762	1.263	1.861	0.431	0.867	1.157	1.116	1.211	0.767	0.698	1.000	0.710	2.067	1.836	1.563	1.075
	1.000	0.661	0.761	0.785	0.623	0.805	0.821	0.735	1.236	1.814	1.659	0.581	1.000	0.977	1.132	1.329	1.058	0.956	1.000	0.606	1.531	1.564	1.718	0.332
	1.207	1.062	1.145	1.239	0.750	1.181	0.882	0.888	1.865	1.737	1.303	0.339	1.000	0.841	1.861	1.114	0.599	0.400		0.583	1.692	2.290	1.960	1.679
	1.000	0.980	0.951	0.715	1.221	0.965	0.784	1.616	1.617	2.110	1.011	0.067	0.803	1.540	0.916	0.410	0.648	0.731		1.551	1.049	2.209	1.026	1.000
	0.887	1.074	1.182	1.285	0.779	0.955	0.730	2.498	2.120	2.505	0.933	1.539	1.167	0.972	0.793	0.681	1.053	1.616		2.732		0.840	1.447	1.665
	0.738	0.945	0.867			1.079	1.000	1.027				0.438	1.159	0.864	0.851	1.084	0.884			3.447		1.776	0.795	
	1.000						1.000						0.833											
	1.000						1.457																	
N	11	9	9	8	8	9	11	9	8	8	8	9	10	9	9	9	9	8	5	9	7	9	9	8
Media	0.9877	1.0000	1.0000	1.0000	1.0000	1.0000	0.9531	1.1632	2.0710	1.7338	1.1871	0.9796	0.9408	1.1141	1.1192	0.9340	0.9026	1.0303	1.0000	1.3716	2.2259	1.3563	1.2261	0.9402
SD	0.1554	0.2205	0.1578	0.2106	0.3297	0.1156	0.2993	0.6603	0.8920	0.5079	0.3847	0.7692	0.1524	0.2937	0.3592	0.2906	0.1979	0.4586	0.0000	1.0333	1.2516	0.7248	0.4510	0.5452
SE	0.0468	0.0735	0.0526	0.0745	0.1166	0.0385	0.0902	0.2201	0.3154	0.1796	0.1360	0.2564	0.0482	0.0979	0.1197	0.0969	0.0660	0.1621	0.0000	0.3444	0.4731	0.2416	0.1503	0.1927

Figure 8

Figure 8J (upper panel)

	NEG/veh	NEG/4AP	ODN/veh	ODN/4AP
	114.6367	168.7425	244.9662	187.7173
	104.8374	232.9807	239.8466	213.5883
	95.75623	212.5008	196.4015	253.5087
	89.20836	161.5531	216.5148	220.0667
	96.55971	348.3558	316.4863	154.858
	99.00161	265.2511		
**N**	**6**	**6**	**5**	**5**
**Media**	**100.0000**	**231.5640**	**242.8431**	**205.9478**
SD	8.7742	69.2656	45.5160	36.9536
**SE**	**3.5821**	**28.2776**	**20.3554**	**16.5262**

Figure 8J (center panel)

	NEG/veh	NEG/4AP	ODN/veh	ODN/4AP
	69.96064	154.6214	78.96081	78.6385
	114.9258	169.3572	78.28458	73.14586
	80.13842	171.7354	69.99998	96.93646
	96.04458	183.4622	37.02048	115.3311
	138.9305	200.3438	80.45357	68.60624
**N**	**5**	**5**	**5**	**5**
**Media**	**100.0000**	**175.9040**	**68.9439**	**86.5316**
SD	27.6173	17.0852	18.3049	19.3679
**SE**	**12.3508**	**7.6407**	**8.1862**	**8.6616**

Figure 8J (lower panel)

	NEG/veh	NEG/4AP	ODN/veh	ODN/4AP
	109.4663	126.39	111.503	183.7737
	125.8986	161.3319	141.0861	183.3982
	99.96003	185.755	120.8976	156.1881
	75.94982	159.5488	114.4821	143.389
	88.72526	126.9813	86.80598	122.2708
		163.2185		195.2917
**N**	**5**	**6**	**5**	**6**
**Media**	**100.0000**	**153.8709**	**114.9550**	**164.0519**
SD	19.1434	23.1090	19.5106	28.1908
**SE**	**8.5612**	**9.4342**	**8.7254**	**11.5089**

Figure 8

Figure 8A

Tukey's multiple comparisons test	Significant	Summary	P Value
0hr			
NEG/veh vs. NEG/4AP	No	ns	>0,9999
NEG/veh vs. ODN/veh	No	ns	>0,9999
NEG/veh vs. ODN/4AP	No	ns	>0,9999
NEG/4AP vs. ODN/veh	No	ns	>0,9999
NEG/4AP vs. ODN/4AP	No	ns	>0,9999
ODN/veh vs. ODN/4AP	No	ns	>0,9999
1hrs			
NEG/veh vs. NEG/4AP	Yes	**	0.0096
NEG/veh vs. ODN/veh	No	ns	0.0991
NEG/veh vs. ODN/4AP	No	ns	0.0884
NEG/4AP vs. ODN/veh	No	ns	0.8587
NEG/4AP vs. ODN/4AP	No	ns	0.8387
ODN/veh vs. ODN/4AP	No	ns	>0,9999
3hrs			
NEG/veh vs. NEG/4AP	No	ns	0.0848
NEG/veh vs. ODN/veh	No	ns	0.1993
NEG/veh vs. ODN/4AP	No	ns	0.3617
NEG/4AP vs. ODN/veh	No	ns	0.966
NEG/4AP vs. ODN/4AP	No	ns	0.9107
ODN/veh vs. ODN/4AP	No	ns	0.9957
6hrs			
NEG/veh vs. NEG/4AP	Yes	**	0.0075
NEG/veh vs. ODN/veh	No	ns	0.6555
NEG/veh vs. ODN/4AP	Yes	*	0.0181
NEG/4AP vs. ODN/veh	No	ns	0.1373
NEG/4AP vs. ODN/4AP	No	ns	0.9916
ODN/veh vs. ODN/4AP	No	ns	0.245
12hrs			
NEG/veh vs. NEG/4AP	Yes	*	0.0333
NEG/veh vs. ODN/veh	No	ns	0.6418
NEG/veh vs. ODN/4AP	No	ns	0.0711
NEG/4AP vs. ODN/veh	No	ns	0.3992
NEG/4AP vs. ODN/4AP	No	ns	>0,9999
ODN/veh vs. ODN/4AP	No	ns	0.4884
24hrs			
NEG/veh vs. NEG/4AP	Yes	*	0.036
NEG/veh vs. ODN/veh	No	ns	0.9859
NEG/veh vs. ODN/4AP	No	ns	0.1903
NEG/4AP vs. ODN/veh	No	ns	0.0967
NEG/4AP vs. ODN/4AP	No	ns	0.9073
ODN/veh vs. ODN/4AP	No	ns	0.3656

Figure 8B

Tukey's multiple comparisons test	Significant	Summary	P Value
0hr			
NEG/veh vs. NEG/4AP	No	ns	>0,9999
NEG/veh vs. ODN/veh	No	ns	>0,9999
NEG/veh vs. ODN/4AP	No	ns	>0,9999
NEG/4AP vs. ODN/veh	No	ns	>0,9999
NEG/4AP vs. ODN/4AP	No	ns	>0,9999
ODN/veh vs. ODN/4AP	No	ns	>0,9999
1hrs			
NEG/veh vs. NEG/4AP	Yes	*	0.0296
NEG/veh vs. ODN/veh	No	ns	0.9965
NEG/veh vs. ODN/4AP	No	ns	>0,9999
NEG/4AP vs. ODN/veh	No	ns	0.0504
NEG/4AP vs. ODN/4AP	Yes	*	0.0326
ODN/veh vs. ODN/4AP	No	ns	0.9981
3hrs			
NEG/veh vs. NEG/4AP	Yes	*	0.0249
NEG/veh vs. ODN/veh	No	ns	0.9976
NEG/veh vs. ODN/4AP	No	ns	0.3326
NEG/4AP vs. ODN/veh	Yes	*	0.041
NEG/4AP vs. ODN/4AP	No	ns	0.6193
ODN/veh vs. ODN/4AP	No	ns	0.4391
6hrs			
NEG/veh vs. NEG/4AP	Yes	****	<0,0001
NEG/veh vs. ODN/veh	No	ns	0.9986
NEG/veh vs. ODN/4AP	Yes	****	<0,0001
NEG/4AP vs. ODN/veh	Yes	****	<0,0001
NEG/4AP vs. ODN/4AP	No	ns	0.9704
ODN/veh vs. ODN/4AP	Yes	****	<0,0001
12hrs			
NEG/veh vs. NEG/4AP	No	ns	0.1726
NEG/veh vs. ODN/veh	No	ns	0.9978
NEG/veh vs. ODN/4AP	Yes	*	0.0211
NEG/4AP vs. ODN/veh	No	ns	0.2443
NEG/4AP vs. ODN/4AP	No	ns	0.7911
ODN/veh vs. ODN/4AP	Yes	*	0.0345
24hrs			
NEG/veh vs. NEG/4AP	No	ns	0.9189
NEG/veh vs. ODN/veh	No	ns	0.9974
NEG/veh vs. ODN/4AP	Yes	****	<0,0001
NEG/4AP vs. ODN/veh	No	ns	0.9741
NEG/4AP vs. ODN/4AP	Yes	***	0.0002
ODN/veh vs. ODN/4AP	Yes	****	<0,0001

Figure 8C

Tukey's multiple comparisons test	Significant	Summary	P Value
0hrs			
NEG/veh vs. NEG/4AP	No	ns	>0,9999
NEG/veh vs. ODN/veh	No	ns	>0,9999
NEG/veh vs. ODN/4AP	No	ns	>0,9999
NEG/4AP vs. ODN/veh	No	ns	>0,9999
NEG/4AP vs. ODN/4AP	No	ns	>0,9999
ODN/veh vs. ODN/4AP	No	ns	>0,9999
1hrs			
NEG/veh vs. NEG/4AP	No	ns	0.9721
NEG/veh vs. ODN/veh	No	ns	0.9203
NEG/veh vs. ODN/4AP	No	ns	0.9869
NEG/4AP vs. ODN/veh	No	ns	0.9972
NEG/4AP vs. ODN/4AP	No	ns	0.8667
ODN/veh vs. ODN/4AP	No	ns	0.7655
3hrs			
NEG/veh vs. NEG/4AP	No	ns	0.2262
NEG/veh vs. ODN/veh	No	ns	0.9855
NEG/veh vs. ODN/4AP	No	ns	0.8102
NEG/4AP vs. ODN/veh	No	ns	0.1138
NEG/4AP vs. ODN/4AP	No	ns	0.7205
ODN/veh vs. ODN/4AP	No	ns	0.6038
6hrs			
NEG/veh vs. NEG/4AP	Yes	****	<0,0001
NEG/veh vs. ODN/veh	No	ns	0.9874
NEG/veh vs. ODN/4AP	Yes	*	0.0214
NEG/4AP vs. ODN/veh	Yes	****	<0,0001
NEG/4AP vs. ODN/4AP	Yes	*	0.0346
ODN/veh vs. ODN/4AP	Yes	*	0.0431
12hrs			
NEG/veh vs. NEG/4AP	Yes	****	<0,0001
NEG/veh vs. ODN/veh	No	ns	>0,9999
NEG/veh vs. ODN/4AP	Yes	*	0.0158
NEG/4AP vs. ODN/veh	Yes	****	<0,0001
NEG/4AP vs. ODN/4AP	Yes	*	0.0358
ODN/veh vs. ODN/4AP	Yes	*	0.0164
24hrs			
NEG/veh vs. NEG/4AP	Yes	****	<0,0001
NEG/veh vs. ODN/veh	No	ns	0.9932
NEG/veh vs. ODN/4AP	Yes	**	0.0033
NEG/4AP vs. ODN/veh	Yes	****	<0,0001
NEG/4AP vs. ODN/4AP	Yes	*	0.034
ODN/veh vs. ODN/4AP	Yes	*	0.0116

Figure 8D

Tukey's multiple comparisons test	Significant	Summary	P Value
0hrs			
NEG/veh vs. NEG/4AP	No	ns	>0,9999
NEG/veh vs. ODN/veh	No	ns	>0,9999
NEG/veh vs. ODN/4AP	No	ns	>0,9999
NEG/4AP vs. ODN/veh	No	ns	>0,9999
NEG/4AP vs. ODN/4AP	No	ns	>0,9999
ODN/veh vs. ODN/4AP	No	ns	>0,9999
1hrs			
NEG/veh vs. NEG/4AP	No	ns	>0,9999
NEG/veh vs. ODN/veh	No	ns	0.9865
NEG/veh vs. ODN/4AP	No	ns	0.9532
NEG/4AP vs. ODN/veh	No	ns	0.9784
NEG/4AP vs. ODN/4AP	No	ns	0.9666
ODN/veh vs. ODN/4AP	No	ns	0.8235
3hrs			
NEG/veh vs. NEG/4AP	No	ns	>0,9999
NEG/veh vs. ODN/veh	No	ns	0.8973
NEG/veh vs. ODN/4AP	No	ns	0.9976
NEG/4AP vs. ODN/veh	No	ns	0.9144
NEG/4AP vs. ODN/4AP	No	ns	0.9986
ODN/veh vs. ODN/4AP	No	ns	0.9563
6hrs			
NEG/veh vs. NEG/4AP	Yes	****	<0,0001
NEG/veh vs. ODN/veh	No	ns	0.9909
NEG/veh vs. ODN/4AP	Yes	*	0.0154
NEG/4AP vs. ODN/veh	Yes	****	<0,0001
NEG/4AP vs. ODN/4AP	Yes	*	0.0155
ODN/veh vs. ODN/4AP	Yes	*	0.0285
12hrs			
NEG/veh vs. NEG/4AP	Yes	***	0.0002
NEG/veh vs. ODN/veh	No	ns	0.996
NEG/veh vs. ODN/4AP	Yes	**	0.0054
NEG/4AP vs. ODN/veh	Yes	****	<0,0001
NEG/4AP vs. ODN/4AP	No	ns	0.7583
ODN/veh vs. ODN/4AP	Yes	**	0.0016
24hrs			
NEG/veh vs. NEG/4AP	Yes	****	<0,0001
NEG/veh vs. ODN/veh	No	ns	0.9511
NEG/veh vs. ODN/4AP	Yes	****	<0,0001
NEG/4AP vs. ODN/veh	Yes	****	<0,0001
NEG/4AP vs. ODN/4AP	No	ns	0.542
ODN/veh vs. ODN/4AP	Yes	****	<0,0001

Figure 8

Figure 8E

Tukey's multiple comparisons test	Significant	Summary	P Value
0hr			
NEG/veh vs. NEG/4AP	No	ns	0.9941
NEG/veh vs. ODN/veh	No	ns	0.9852
NEG/veh vs. ODN/4AP	No	ns	0.9892
NEG/4AP vs. ODN/veh	No	ns	0.9996
NEG/4AP vs. ODN/4AP	No	ns	>0,9999
ODN/veh vs. ODN/4AP	No	ns	>0,9999
1hrs			
NEG/veh vs. NEG/4AP	No	ns	0.9405
NEG/veh vs. ODN/veh	No	ns	>0,9999
NEG/veh vs. ODN/4AP	No	ns	0.896
NEG/4AP vs. ODN/veh	No	ns	0.9413
NEG/4AP vs. ODN/4AP	No	ns	0.9992
ODN/veh vs. ODN/4AP	No	ns	0.8986
3hrs			
NEG/veh vs. NEG/4AP	No	ns	0.9929
NEG/veh vs. ODN/veh	No	ns	0.8099
NEG/veh vs. ODN/4AP	No	ns	0.7875
NEG/4AP vs. ODN/veh	No	ns	0.6513
NEG/4AP vs. ODN/4AP	No	ns	0.6247
ODN/veh vs. ODN/4AP	No	ns	>0,9999
6hrs			
NEG/veh vs. NEG/4AP	Yes	****	<0,0001
NEG/veh vs. ODN/veh	No	ns	0.9875
NEG/veh vs. ODN/4AP	Yes	*	0.0322
NEG/4AP vs. ODN/veh	Yes	****	<0,0001
NEG/4AP vs. ODN/4AP	Yes	**	0.0032
ODN/veh vs. ODN/4AP	Yes	**	0.0078
12hrs			
NEG/veh vs. NEG/4AP	Yes	****	<0,0001
NEG/veh vs. ODN/veh	No	ns	0.9964
NEG/veh vs. ODN/4AP	No	ns	0.1931
NEG/4AP vs. ODN/veh	Yes	****	<0,0001
NEG/4AP vs. ODN/4AP	Yes	*	0.0325
ODN/veh vs. ODN/4AP	No	ns	0.2572
24hrs			
NEG/veh vs. NEG/4AP	Yes	****	<0,0001
NEG/veh vs. ODN/veh	No	ns	0.7581
NEG/veh vs. ODN/4AP	Yes	*	0.0166
NEG/4AP vs. ODN/veh	Yes	****	<0,0001
NEG/4AP vs. ODN/4AP	Yes	*	0.0297
ODN/veh vs. ODN/4AP	No	ns	0.196

Figure 8F

Tukey's multiple comparisons test	Significant	Summary	P Value
0hrs			
NEG/veh vs. NEG/4AP	No	ns	0.9923
NEG/veh vs. ODN/veh	No	ns	0.9807
NEG/veh vs. ODN/4AP	No	ns	0.9859
NEG/4AP vs. ODN/veh	No	ns	0.9995
NEG/4AP vs. ODN/4AP	No	ns	>0,9999
ODN/veh vs. ODN/4AP	No	ns	>0,9999
1hrs			
NEG/veh vs. NEG/4AP	No	ns	0.9144
NEG/veh vs. ODN/veh	No	ns	0.8535
NEG/veh vs. ODN/4AP	No	ns	0.7744
NEG/4AP vs. ODN/veh	No	ns	0.9988
NEG/4AP vs. ODN/4AP	No	ns	0.9903
ODN/veh vs. ODN/4AP	No	ns	0.9987
3hrs			
NEG/veh vs. NEG/4AP	Yes	*	0.0357
NEG/veh vs. ODN/veh	No	ns	0.8668
NEG/veh vs. ODN/4AP	No	ns	0.1002
NEG/4AP vs. ODN/veh	No	ns	0.2114
NEG/4AP vs. ODN/4AP	No	ns	0.976
ODN/veh vs. ODN/4AP	No	ns	0.4186
6hrs			
NEG/veh vs. NEG/4AP	Yes	***	0.0004
NEG/veh vs. ODN/veh	No	ns	>0,9999
NEG/veh vs. ODN/4AP	No	ns	0.22
NEG/4AP vs. ODN/veh	Yes	***	0.0002
NEG/4AP vs. ODN/4AP	No	ns	0.1106
ODN/veh vs. ODN/4AP	No	ns	0.1769
12hrs			
NEG/veh vs. NEG/4AP	Yes	*	0.0133
NEG/veh vs. ODN/veh	No	ns	0.9996
NEG/veh vs. ODN/4AP	Yes	*	0.0287
NEG/4AP vs. ODN/veh	Yes	*	0.0138
NEG/4AP vs. ODN/4AP	No	ns	0.9985
ODN/veh vs. ODN/4AP	Yes	*	0.0304
24hrs			
NEG/veh vs. NEG/4AP	No	ns	0.0829
NEG/veh vs. ODN/veh	No	ns	0.8886
NEG/veh vs. ODN/4AP	Yes	**	0.0013
NEG/4AP vs. ODN/veh	No	ns	0.3831
NEG/4AP vs. ODN/4AP	No	ns	0.4775
ODN/veh vs. ODN/4AP	Yes	*	0.0188

Figure 8G

Tukey's multiple comparisons test	Significant	Summary	P Value
0hrs			
NEG/veh vs. NEG/4AP	No	ns	0.9933
NEG/veh vs. ODN/veh	No	ns	0.9676
NEG/veh vs. ODN/4AP	No	ns	>0,9999
NEG/4AP vs. ODN/veh	No	ns	0.9972
NEG/4AP vs. ODN/4AP	No	ns	0.9964
ODN/veh vs. ODN/4AP	No	ns	0.9857
1hrs			
NEG/veh vs. NEG/4AP	No	ns	0.9329
NEG/veh vs. ODN/veh	No	ns	0.6378
NEG/veh vs. ODN/4AP	No	ns	0.9674
NEG/4AP vs. ODN/veh	No	ns	0.9359
NEG/4AP vs. ODN/4AP	No	ns	0.9991
ODN/veh vs. ODN/4AP	No	ns	0.8878
3hrs			
NEG/veh vs. NEG/4AP	No	ns	0.6326
NEG/veh vs. ODN/veh	No	ns	0.8472
NEG/veh vs. ODN/4AP	No	ns	0.1521
NEG/4AP vs. ODN/veh	No	ns	0.9817
NEG/4AP vs. ODN/4AP	No	ns	0.7923
ODN/veh vs. ODN/4AP	No	ns	0.5638
6hrs			
NEG/veh vs. NEG/4AP	Yes	*	0.0116
NEG/veh vs. ODN/veh	No	ns	0.952
NEG/veh vs. ODN/4AP	No	ns	0.5629
NEG/4AP vs. ODN/veh	Yes	**	0.0011
NEG/4AP vs. ODN/4AP	No	ns	0.233
ODN/veh vs. ODN/4AP	No	ns	0.2235
12hrs			
NEG/veh vs. NEG/4AP	Yes	*	0.0138
NEG/veh vs. ODN/veh	No	ns	0.9995
NEG/veh vs. ODN/4AP	Yes	*	0.0128
NEG/4AP vs. ODN/veh	Yes	**	0.0074
NEG/4AP vs. ODN/4AP	No	ns	>0,9999
ODN/veh vs. ODN/4AP	Yes	**	0.0066
24hrs			
NEG/veh vs. NEG/4AP	Yes	*	0.0126
NEG/veh vs. ODN/veh	No	ns	0.9126
NEG/veh vs. ODN/4AP	Yes	**	0.0031
NEG/4AP vs. ODN/veh	No	ns	0.0943
NEG/4AP vs. ODN/4AP	No	ns	0.9531
ODN/veh vs. ODN/4AP	Yes	*	0.0306

Figure 8H

Tukey's multiple comparisons test	Significant	Summary	P Value
0hrs			
NEG/veh vs. NEG/4AP	No	ns	0.9986
NEG/veh vs. ODN/veh	No	ns	0.9969
NEG/veh vs. ODN/4AP	No	ns	>0,9999
NEG/4AP vs. ODN/veh	No	ns	>0,9999
NEG/4AP vs. ODN/4AP	No	ns	0.9983
ODN/veh vs. ODN/4AP	No	ns	0.9968
1hrs			
NEG/veh vs. NEG/4AP	No	ns	0.9099
NEG/veh vs. ODN/veh	No	ns	0.9665
NEG/veh vs. ODN/4AP	No	ns	0.4304
NEG/4AP vs. ODN/veh	No	ns	0.9971
NEG/4AP vs. ODN/4AP	No	ns	0.8304
ODN/veh vs. ODN/4AP	No	ns	0.7201
3hrs			
NEG/veh vs. NEG/4AP	Yes	***	0.0002
NEG/veh vs. ODN/veh	No	ns	0.9621
NEG/veh vs. ODN/4AP	Yes	****	<0,0001
NEG/4AP vs. ODN/veh	Yes	**	0.0013
NEG/4AP vs. ODN/4AP	No	ns	0.9394
ODN/veh vs. ODN/4AP	Yes	***	0.0002
6hrs			
NEG/veh vs. NEG/4AP	Yes	*	0.027
NEG/veh vs. ODN/veh	No	ns	0.9937
NEG/veh vs. ODN/4AP	No	ns	0.4949
NEG/4AP vs. ODN/veh	Yes	**	0.0097
NEG/4AP vs. ODN/4AP	No	ns	0.4433
ODN/veh vs. ODN/4AP	No	ns	0.3151
12hrs			
NEG/veh vs. NEG/4AP	No	ns	0.8893
NEG/veh vs. ODN/veh	No	ns	0.9805
NEG/veh vs. ODN/4AP	No	ns	0.8077
NEG/4AP vs. ODN/veh	No	ns	0.6742
NEG/4AP vs. ODN/4AP	No	ns	0.9987
ODN/veh vs. ODN/4AP	No	ns	0.5517
24hrs			
NEG/veh vs. NEG/4AP	No	ns	0.9998
NEG/veh vs. ODN/veh	No	ns	0.9994
NEG/veh vs. ODN/4AP	No	ns	0.9953
NEG/4AP vs. ODN/veh	No	ns	0.9971
NEG/4AP vs. ODN/4AP	No	ns	0.9987
ODN/veh vs. ODN/4AP	No	ns	0.9857

Figure 8

Figure 8J (upper panel)			
Tukey's multiple comparisons test	Significant′	Summary	P Value
NEG :veh vs. NEG :4AP	Yes	***	0.0005
NEG :veh vs. ODN :veh	Yes	***	0.0004
NEG :veh vs. ODN :4AP	Yes	**	0.0065
NEG :4AP vs. ODN :veh	No	ns	0.9769
NEG :4AP vs. ODN :4AP	No	ns	0.795
ODN :veh vs. ODN :4AP	No	ns	0.594

Figure 8J (center panel)			
Tukey's multiple comparisons test	Significant′	Summary	P Value
NEG :veh vs. NEG :4AP	Yes	***	0.0002
NEG :veh vs. ODN :veh	No	ns	0.1307
NEG :veh vs. ODN :4AP	No	ns	0.7439
NEG :4AP vs. ODN :veh	Yes	****	< 0.0001
NEG :4AP vs. ODN :4AP	Yes	****	< 0.0001
ODN :veh vs. ODN :4AP	No	ns	0.5618

Figure 8J (lower panel)			
Tukey's multiple comparisons test	Significant′	Summary	P Value
NEG :veh vs. NEG :4AP	Yes	**	0.0059
NEG :veh vs. ODN :veh	No	ns	0.7389
NEG :veh vs. ODN :4AP	Yes	**	0.0012
NEG :4AP vs. ODN :veh	No	ns	0.055
NEG :4AP vs. ODN :4AP	No	ns	0.8702
ODN :veh vs. ODN :4AP	Yes	*	0.0123

Fig.8I_REST dataset

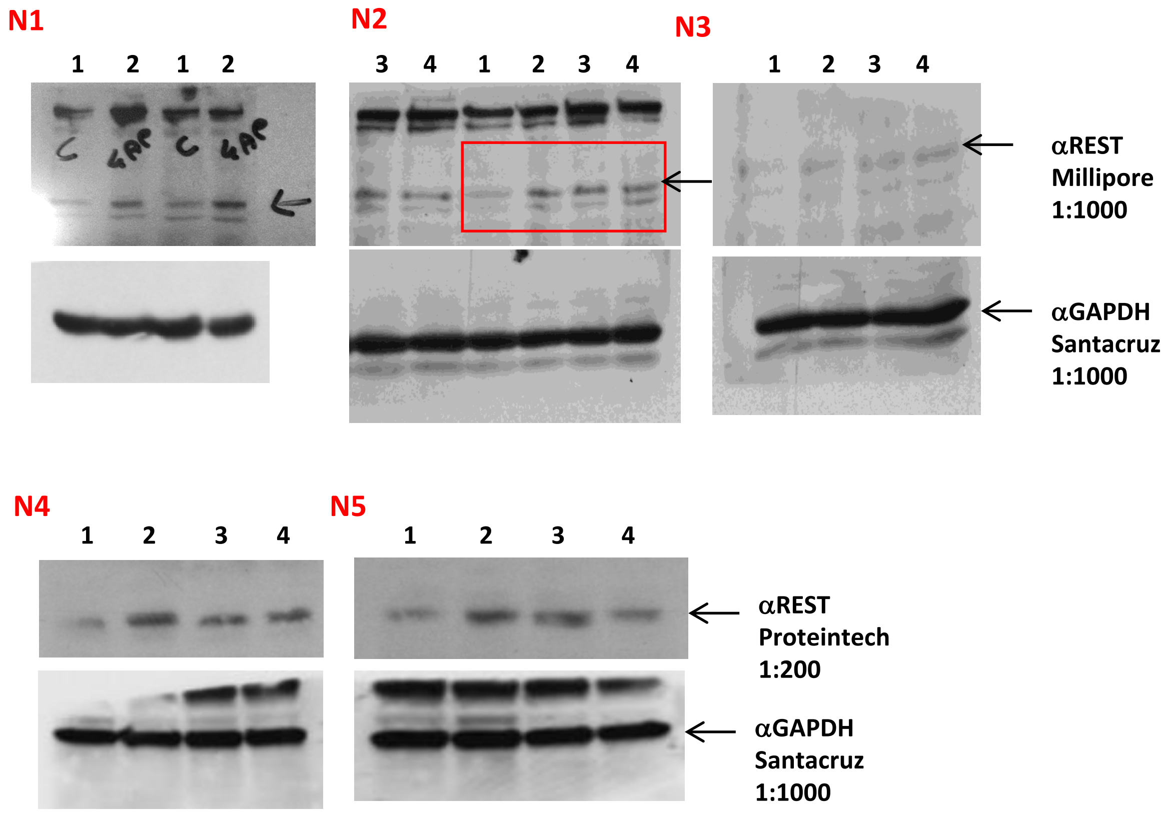

Arrows indicate the REST or GAPDH specific bands.
The red square indicates the representative blot of Figure 8I.

Layout legend: 1 is for NEG veh
2 is for NEG 4AP
3 is for ODN veh
4 is for ODN 4AP

Fig.8I_Npas4 dataset

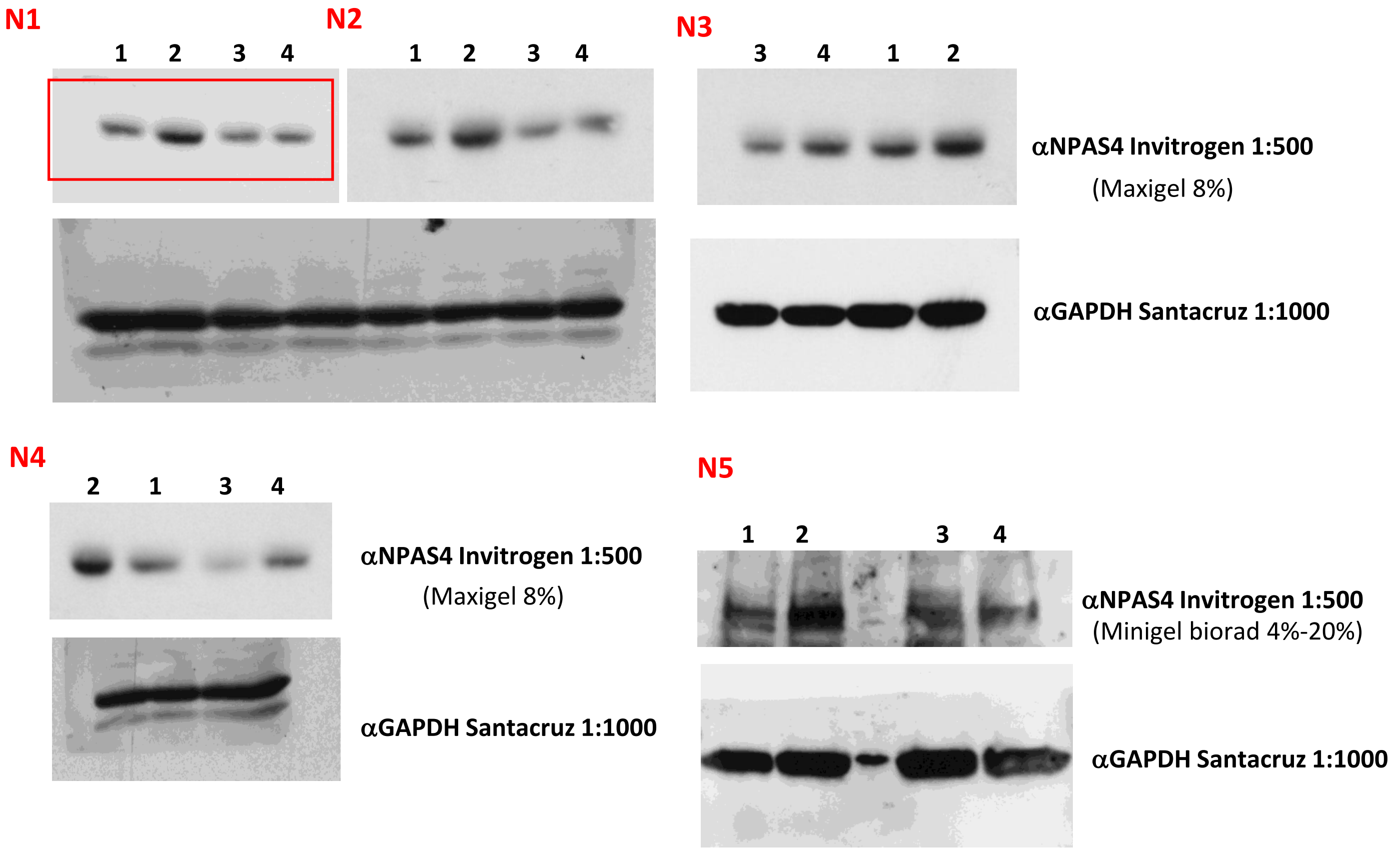

The red square indicates the representative blot of Figure 8I.

Layout legend: 1 is for NEG veh
2 is for NEG 4AP
3 is for ODN veh
4 is for ODN 4AP

Fig.8I_Syt4 dataset

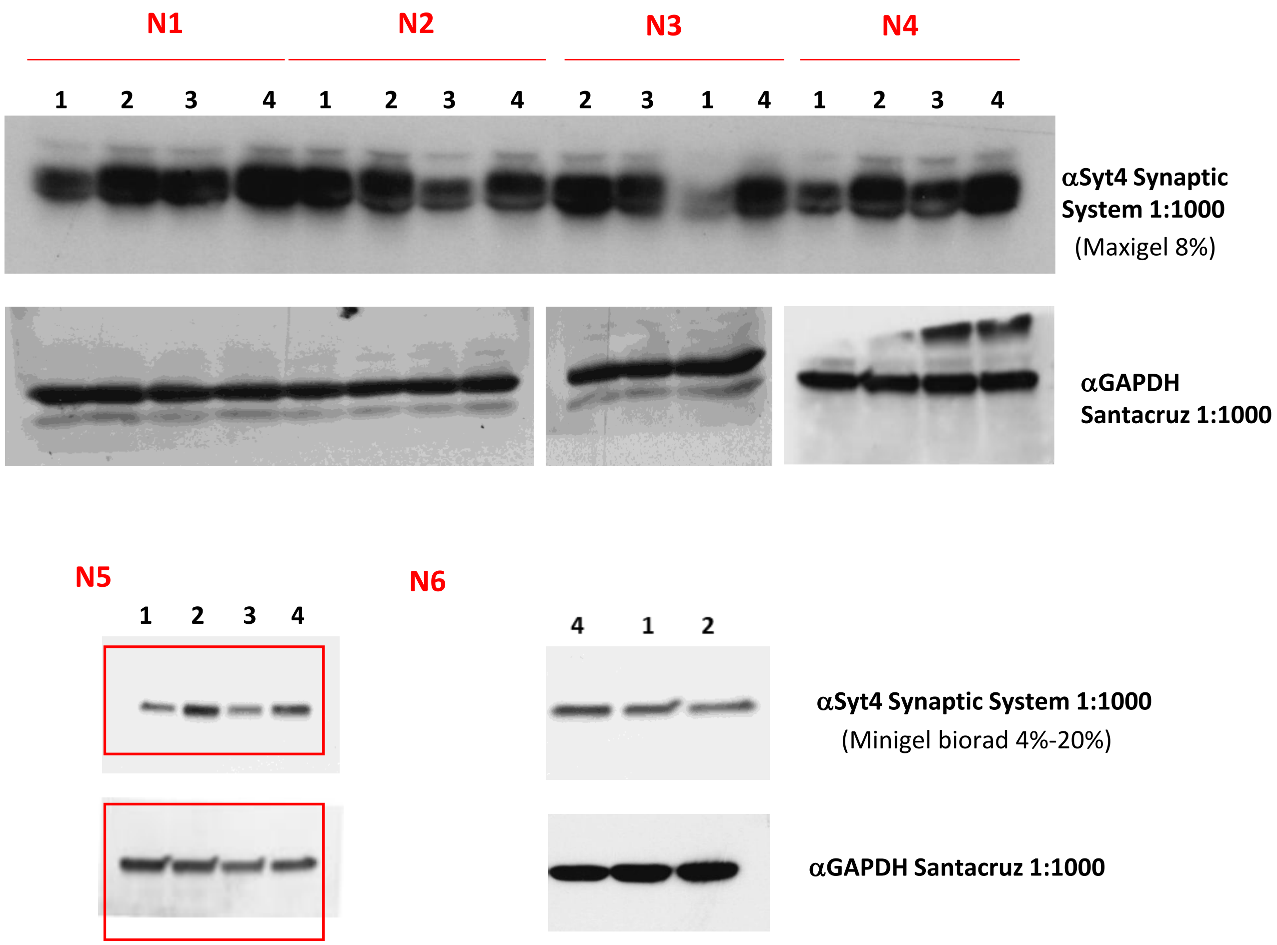

Red squares indicate the representative blots (Syt4, GAPDH) of Figure 8I.

Layout legend: 1 is for NEG veh
2 is for NEG 4AP
3 is for ODN veh
4 is for ODN 4AP
