# Supplementary material for: REST/NRSF drives homeostatic plasticity of inhibitory synapses in a target-dependent fashion
Source: eLife. 2021 Dec 2;10:e69058. doi: 10.7554/eLife.69058 (PMC8639147; doi:10.7554/eLife.69058)
Supplement: Figure 9—source data 1. [file elife-69058-fig9-data1.pdf]

Figure 9

Figure 9A

	NEG/veh					NEG/4AP					ODN/veh					ODN/4AP				
	1hrs	3hrs	6hrs	12hrs	24hrs	1hrs	3hrs	6hrs	12hrs	24hrs	1hrs	3hrs	6hrs	12hrs	24hrs	1hrs	3hrs	6hrs	12hrs	24hrs
	0.905	0.925	1.063	0.974	1.245	1.283	0.896	0.842	1.924	0.863	0.530	0.703	1.161	0.709	0.755	1.030	1.081	0.697	1.283	1.134
	1.121	1.167	0.938	1.026	0.823	1.123	1.079	1.135	0.520	1.520	1.029	1.396	0.834	0.982	1.022	0.883	1.127	1.080	1.337	1.933
	0.974	0.908	0.999	0.976	0.932	1.451		0.847	1.595	1.397	0.993	0.937	1.011	0.590	0.829	0.791	0.821	0.930	1.463	1.048
	1.038	0.940	0.842	1.144	0.929	1.764	1.163	0.979	1.183	1.215	0.855	0.952	0.925	1.119	0.633	0.827	1.376	1.024	1.026	1.258
	0.982	0.957	0.981	0.880	0.873	0.911	1.200	1.130	0.935	0.916	0.881	1.041	0.817	0.937	0.840	0.797	1.543	1.134	0.830	0.964
	0.980	1.103	1.176	0.826	1.198	0.983	0.885	0.985	1.022	1.207	1.055	1.091	0.962	0.782	0.978	1.016	1.228	0.902	0.476	1.477
	0.924	1.078	1.043	1.228	1.078	0.778	1.185	1.271	0.524	0.911	0.881	0.976	0.903	0.486	1.461	0.839	0.829	0.780		1.198
	1.193	0.974	0.957	0.946	0.927	0.849	0.728	1.282		0.950	0.780	1.029	0.505	0.474	1.264	0.825	0.824	0.861		1.247
	0.882	0.948			0.995	0.805	0.760	1.018		1.152	0.571	0.954	0.641				0.917	0.873		
**N**	**9**	**9**	**8**	**8**	**9**	**9**	**8**	**9**	**7**	**9**	**9**	**9**	**9**	**8**	**8**	**8**	**9**	**9**	**6**	**8**
**Media**	**1.0000**	**1.0000**	**1.0000**	**1.0000**	**1.0000**	**1.1051**	**0.9870**	**1.0543**	**1.1004**	**1.1255**	**0.8416**	**1.0089**	**0.8620**	**0.7597**	**0.9727**	**0.8760**	**1.0829**	**0.9202**	**1.0693**	**1.2825**
SD	0.1022	0.0919	0.0984	0.1323	0.1448	0.3356	0.1931	0.1626	0.5215	0.2330	0.1875	0.1818	0.1956	0.2387	0.2743	0.0950	0.2618	0.1404	0.3696	0.3043
**SE**	**0.0341**	**0.0306**	**0.0348**	**0.0468**	**0.0483**	**0.1119**	**0.0683**	**0.0542**	**0.1971**	**0.0777**	**0.0625**	**0.0606**	**0.0652**	**0.0844**	**0.0970**	**0.0336**	**0.0873**	**0.0468**	**0.1509**	**0.1076**

Figure 9B

	NEG/veh					NEG/4AP					ODN/veh					ODN/4AP				
	1hrs	3hrs	6hrs	12hrs	24hrs	1hrs	3hrs	6hrs	12hrs	24hrs	1hrs	3hrs	6hrs	12hrs	24hrs	1hrs	3hrs	6hrs	12hrs	24hrs
	1.042	0.921	1.041	1.116	1.059	0.656	0.903	1.092	1.545	1.072	1.622	0.731	1.153	0.613	1.026	0.987	1.087	0.823	1.442	1.250
	1.061	1.151	1.012	0.884	0.998	0.536	1.106	1.207	3.906	1.119	0.887	1.610	1.128	0.649	1.094	0.853	1.307	1.230	1.208	2.133
	0.897	0.928	0.946	0.908	0.943	0.743	0.998	1.213	2.216	1.098	0.673	0.948	1.076	1.350	1.349	1.042	0.956	0.923	1.662	0.663
	1.084	0.956	0.849	1.092	0.980	1.746	1.302	1.031	2.357	2.000	0.805	0.975	0.886	1.320	0.590	0.872	1.089	0.984	1.584	0.810
	0.941	0.884	0.961	0.782	0.794	0.918	0.657	1.170	1.669	2.000	0.929	0.899	0.809	0.297	0.832	0.798	1.098	0.925	0.885	0.697
	0.975	1.160	1.191	1.269	1.226	1.269	0.819	1.135	2.000	1.001	0.888	1.078	1.026	0.479	1.117	0.797	1.166	0.931	0.461	1.366
	0.994	0.956	1.062	0.949	1.074	0.800	1.238	1.348	2.000	2.940	0.959	1.084	0.839	0.603	1.230	1.223	1.043	0.621		1.426
	1.127	0.954	0.938		1.097	0.855	0.919	1.161	2.000	2.000	0.782	0.962	0.478		1.561	0.990	0.962	0.706		1.219
	0.880	1.090			0.829	0.724		1.545	3.000	0.931	0.805	0.987	0.508			0.883	0.882	0.932		
										2.000										
										3.000										
**N**	**9**	**9**	**8**	**7**	**9**	**9**	**8**	**9**	**9**	**11**	**9**	**9**	**9**	**7**	**8**	**9**	**9**	**9**	**6**	**8**
**Media**	**1.0000**	**1.0000**	**1.0000**	**1.0000**	**1.0000**	**0.9163**	**0.9926**	**1.2113**	**2.2992**	**1.7420**	**0.9278**	**1.0304**	**0.8783**	**0.7586**	**1.0999**	**0.9382**	**1.0656**	**0.8972**	**1.2070**	**1.1955**
SD	0.0851	0.1044	0.1022	0.1664	0.1344	0.3723	0.2155	0.1528	0.7347	0.7577	0.2746	0.2410	0.2505	0.4110	0.2997	0.1375	0.1262	0.1735	0.4613	0.4848
**SE**	**0.0284**	**0.0348**	**0.0362**	**0.0629**	**0.0448**	**0.1241**	**0.0762**	**0.0509**	**0.2449**	**0.2285**	**0.0915**	**0.0803**	**0.0835**	**0.1554**	**0.1060**	**0.0458**	**0.0421**	**0.0578**	**0.1883**	**0.1714**

Figure 9C

	NEG/veh					NEG/4AP					ODN/veh					ODN/4AP				
	1hrs	3hrs	6hrs	12hrs	24hrs	1hrs	3hrs	6hrs	12hrs	24hrs	1hrs	3hrs	6hrs	12hrs	24hrs	1hrs	3hrs	6hrs	12hrs	24hrs
	0.885	0.824	1.209	1.201	0.828	0.631	0.946	1.054	1.344	1.056	1.835	0.662	1.481	0.874	0.897	0.902	1.309	0.867	0.927	1.470
	0.962	1.199	1.306	0.799	0.757	0.827	1.315	1.352	3.508	1.252	0.907	1.313	0.970	0.824	0.829	0.835	1.145	1.491	1.378	1.437
	1.153	0.977	0.485	0.809	1.414	0.972	1.269	1.188	1.929	1.223	0.977	0.893	1.165	1.045	1.122	1.100	0.812	1.315	0.646	0.658
	1.092	0.913	0.900	1.191	0.972	2.094	1.492	0.946	2.494	1.243	0.801	1.007	0.753	0.608	0.520	0.780	1.439	1.185	1.024	0.770
	0.910	0.898	0.909	0.937	0.880	1.032	0.921	1.246	0.665	2.000	0.893	1.026	0.738	2.000	0.652	0.828	1.652	0.845	1.081	0.725
	0.998	1.189	1.191	1.187	1.148	0.957	1.176	0.763	3.000	3.000	1.226	1.046	0.994		1.173	1.032	1.302	0.791	0.522	1.052
	0.919	0.955		0.876	0.920	0.768	0.835		3.000	1.059	0.945	1.096			1.338	1.119	0.922			1.141
	1.132	0.901			0.926	0.726	0.741		3.000	1.020	0.617	1.073			1.285	0.840	1.009			1.011
	0.949	1.144			1.154	0.781				1.115	0.773	1.122				0.683	0.824			
										3.000										
**N**	**9**	**9**	**6**	**7**	**9**	**9**	**8**	**6**	**8**	**10**	**9**	**9**	**6**	**5**	**8**	**9**	**9**	**6**	**6**	**8**
**Media**	**1.0000**	**1.0000**	**1.0000**	**1.0000**	**1.0000**	**0.9766**	**1.0869**	**1.0914**	**2.3676**	**1.5969**	**0.9972**	**1.0265**	**1.0168**	**1.0703**	**0.9770**	**0.9020**	**1.1570**	**1.0825**	**0.9297**	**1.0330**
SD	0.1008	0.1404	0.3026	0.1861	0.2039	0.4384	0.2638	0.2152	0.9749	0.7909	0.3550	0.1766	0.2783	0.5425	0.2995	0.1499	0.2908	0.2897	0.3098	0.3096
**SE**	**0.0336**	**0.0468**	**0.1235**	**0.0703**	**0.0680**	**0.1461**	**0.0933**	**0.0879**	**0.3447**	**0.2501**	**0.1183**	**0.0589**	**0.1136**	**0.2426**	**0.1059**	**0.0500**	**0.0969**	**0.1183**	**0.1265**	**0.1095**

Figure 9

Figure 9E (upper panel)

	NEG/veh	NEG/4AP	ODN/veh	ODN/4AP
	87.09161	165.7334	96.10221	85.13294
	89.52753	153.4494	92.14027	102.0385
	103.469	154.2205	159.1166	126.0904
	104.9917	128.1579	94.40748	90.26642
	105.0432	120.2007	79.89907	75.20193
	109.8769	123.2916	118.624	
N	6	6	6	5
Media	100.0000	140.8423	106.7150	95.7460
SD	9.3406	19.2483	28.5776	19.5263
SE	3.8133	7.8581	11.6667	8.7324

Figure 9E (lower panel)

	NEG/veh	NEG/4AP	ODN/veh	ODN/4AP
	85.31045	158.3373	150.8868	144.4432
	107.1667	142.093	90.13124	88.21407
	96.08551	142.838	112.0061	125.9389
	103.3632	123.0061	88.31656	87.40131
	114.7433	142.1101	67.73577	138.2752
	93.33085	146.0375	112.3676	130.3586
N	6	6	6	6
Media	100.0000	142.4037	103.5740	119.1052
SD	10.5415	11.3466	28.5825	25.0689
SE	4.3036	4.6322	11.6688	10.2343

Figure 9

Figure 9A

Tukey's multiple comparisons t	Significant?	Summary	P Value
1hrs			
NEG/veh vs. NEG/4AP	No	ns	0.767
NEG/veh vs. ODN/veh	No	ns	0.464
NEG/veh vs. ODN/4AP	No	ns	0.684
NEG/4AP vs. ODN/veh	Yes	ns	0.076
NEG/4AP vs. ODN/4AP	No	ns	0.175
ODN/veh vs. ODN/4AP	No	ns	0.990
3hrs			
NEG/veh vs. NEG/4AP	No	ns	0.999
NEG/veh vs. ODN/veh	No	ns	1.000
NEG/veh vs. ODN/4AP	No	ns	0.870
NEG/4AP vs. ODN/veh	No	ns	0.997
NEG/4AP vs. ODN/4AP	No	ns	0.826
ODN/veh vs. ODN/4AP	No	ns	0.904
6hrs			
NEG/veh vs. NEG/4AP	No	ns	0.962
NEG/veh vs. ODN/veh	No	ns	0.606
NEG/veh vs. ODN/4AP	No	ns	0.892
NEG/4AP vs. ODN/veh	No	ns	0.290
NEG/4AP vs. ODN/4AP	No	ns	0.605
ODN/veh vs. ODN/4AP	No	ns	0.950
12hrs			
NEG/veh vs. NEG/4AP	No	ns	0.834
NEG/veh vs. ODN/veh	No	ns	0.161
NEG/veh vs. ODN/4AP	No	ns	0.944
NEG/4AP vs. ODN/veh	Yes	*	0.025
NEG/4AP vs. ODN/4AP	No	ns	0.995
ODN/veh vs. ODN/4AP	Yes	ns	0.065
24hrs			
NEG/veh vs. NEG/4AP	No	ns	0.655
NEG/veh vs. ODN/veh	No	ns	0.995
NEG/veh vs. ODN/4AP	Yes	ns	0.060
NEG/4AP vs. ODN/veh	No	ns	0.522
NEG/4AP vs. ODN/4AP	No	ns	0.498
ODN/veh vs. ODN/4AP	Yes	*	0.039

Figure 9B

Tukey's multiple comparisons t	Significant'	Summary	P Value
1hrs			
NEG/veh vs. NEG/4AP	No	ns	0.958
NEG/veh vs. ODN/veh	No	ns	0.972
NEG/veh vs. ODN/4AP	No	ns	0.982
NEG/4AP vs. ODN/veh	No	ns	1.000
NEG/4AP vs. ODN/4AP	No	ns	0.999
ODN/veh vs. ODN/4AP	No	ns	> 0.9999
3hrs			
NEG/veh vs. NEG/4AP	No	ns	> 0.9999
NEG/veh vs. ODN/veh	No	ns	0.998
NEG/veh vs. ODN/4AP	No	ns	0.979
NEG/4AP vs. ODN/veh	No	ns	0.996
NEG/4AP vs. ODN/4AP	No	ns	0.974
ODN/veh vs. ODN/4AP	No	ns	0.997
6hrs			
NEG/veh vs. NEG/4AP	No	ns	0.604
NEG/veh vs. ODN/veh	No	ns	0.892
NEG/veh vs. ODN/4AP	No	ns	0.931
NEG/4AP vs. ODN/veh	No	ns	0.189
NEG/4AP vs. ODN/4AP	No	ns	0.234
ODN/veh vs. ODN/4AP	No	ns	1.000
12hrs			
NEG/veh vs. NEG/4AP	Yes	****	< 0.0001
NEG/veh vs. ODN/veh	No	ns	0.574
NEG/veh vs. ODN/4AP	No	ns	0.715
NEG/4AP vs. ODN/veh	Yes	****	< 0.0001
NEG/4AP vs. ODN/4AP	Yes	****	< 0.0001
ODN/veh vs. ODN/4AP	No	ns	0.104
24hrs			
NEG/veh vs. NEG/4AP	Yes	****	< 0.0001
NEG/veh vs. ODN/veh	No	ns	0.937
NEG/veh vs. ODN/4AP	No	ns	0.662
NEG/4AP vs. ODN/veh	Yes	***	0.001
NEG/4AP vs. ODN/4AP	Yes	**	0.006
ODN/veh vs. ODN/4AP	No	ns	0.948

Figure 9C

Tukey's multiple comparisons tes	Significant'	Summary	P Value
1hrs			
NEG/veh vs. NEG/4AP	No	ns	0.999
NEG/veh vs. ODN/veh	No	ns	> 0.9999
NEG/veh vs. ODN/4AP	No	ns	0.954
NEG/4AP vs. ODN/veh	No	ns	1.000
NEG/4AP vs. ODN/4AP	No	ns	0.979
ODN/veh vs. ODN/4AP	No	ns	0.958
3hrs			
NEG/veh vs. NEG/4AP	No	ns	0.970
NEG/veh vs. ODN/veh	No	ns	0.999
NEG/veh vs. ODN/4AP	No	ns	0.838
NEG/4AP vs. ODN/veh	No	ns	0.990
NEG/4AP vs. ODN/4AP	No	ns	0.984
ODN/veh vs. ODN/4AP	No	ns	0.899
6hrs			
NEG/veh vs. NEG/4AP	No	ns	0.979
NEG/veh vs. ODN/veh	No	ns	1.000
NEG/veh vs. ODN/4AP	No	ns	0.984
NEG/4AP vs. ODN/veh	No	ns	0.988
NEG/4AP vs. ODN/4AP	No	ns	> 0.9999
ODN/veh vs. ODN/4AP	No	ns	0.992
12hrs			
NEG/veh vs. NEG/4AP	Yes	****	< 0.0001
NEG/veh vs. ODN/veh	No	ns	0.991
NEG/veh vs. ODN/4AP	No	ns	0.989
NEG/4AP vs. ODN/veh	Yes	****	< 0.0001
NEG/4AP vs. ODN/4AP	Yes	****	< 0.0001
ODN/veh vs. ODN/4AP	No	ns	0.937
24hrs			
NEG/veh vs. NEG/4AP	Yes	**	0.008
NEG/veh vs. ODN/veh	No	ns	0.999
NEG/veh vs. ODN/4AP	No	ns	0.998
NEG/4AP vs. ODN/veh	Yes	**	0.007
NEG/4AP vs. ODN/4AP	Yes	*	0.018
ODN/veh vs. ODN/4AP	No	ns	0.992

Figure 9

Figure 9E (upper panel)			
Tukey's multiple comparisons tes	Significan	Summary	P Value
NEG :veh vs. NEG :4AP	Yes	*	0.0125
NEG :veh vs. ODN :veh	No	ns	0.9397
NEG :veh vs. ODN :4AP	No	ns	0.9855
NEG :4AP vs. ODN :veh	Yes	*	0.0419
NEG :4AP vs. ODN :4AP	Yes	**	0.0084
ODN :veh vs. ODN :4AP	No	ns	0.8108

Figure 9E (lower panel)			
Tukey's multiple comparisons tes	Significan	Summary	P Value
NEG :veh vs. NEG :4AP	Yes	**	0.0094
NEG :veh vs. ODN :veh	No	ns	0.9902
NEG :veh vs. ODN :4AP	No	ns	0.3946
NEG :4AP vs. ODN :veh	Yes	*	0.0182
NEG :4AP vs. ODN :4AP	No	ns	0.2337
ODN :veh vs. ODN :4AP	No	ns	0.5671

Fig.9D_Gad67 dataset

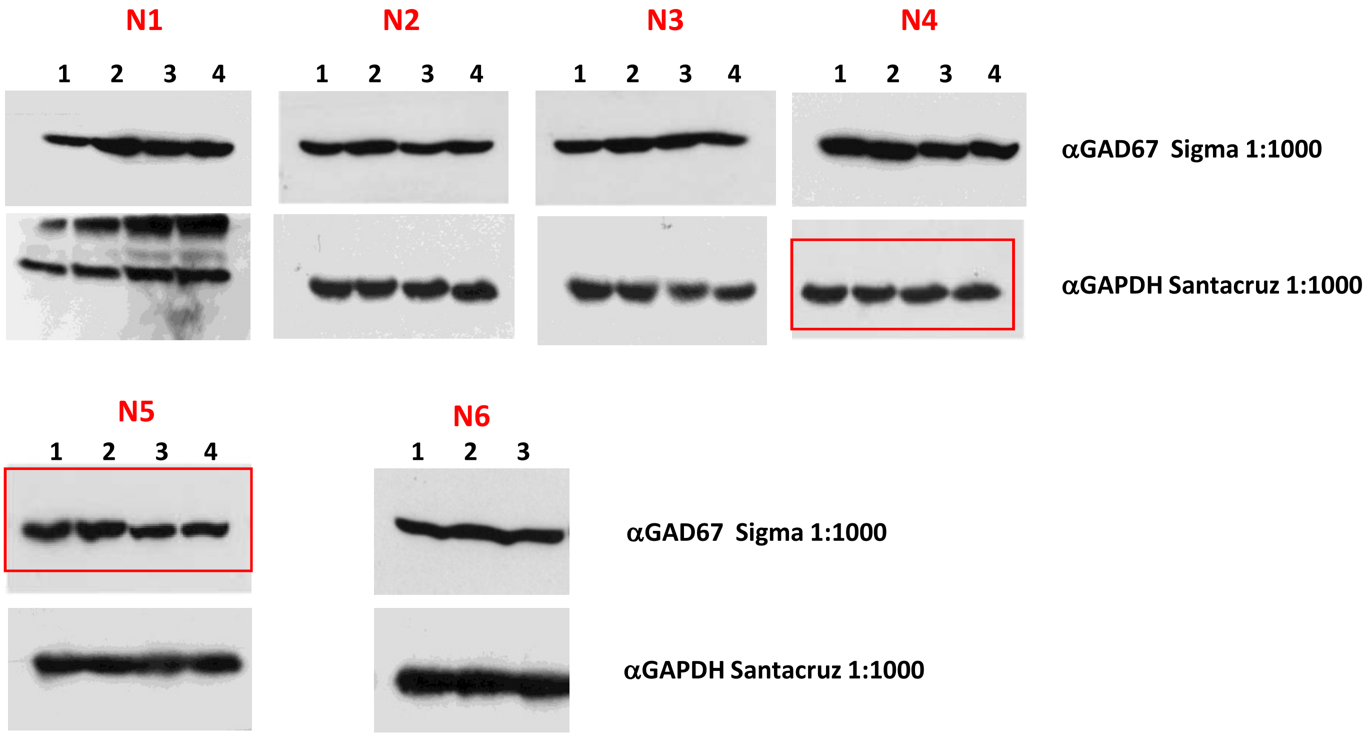

Red squares indicate the representative blot of Figure 9D.

Layout legend: 1 is for NEG veh
2 is for NEG 4AP
3 is for ODN veh
4 is for ODN 4AP

Fig.9D_vGAT dataset

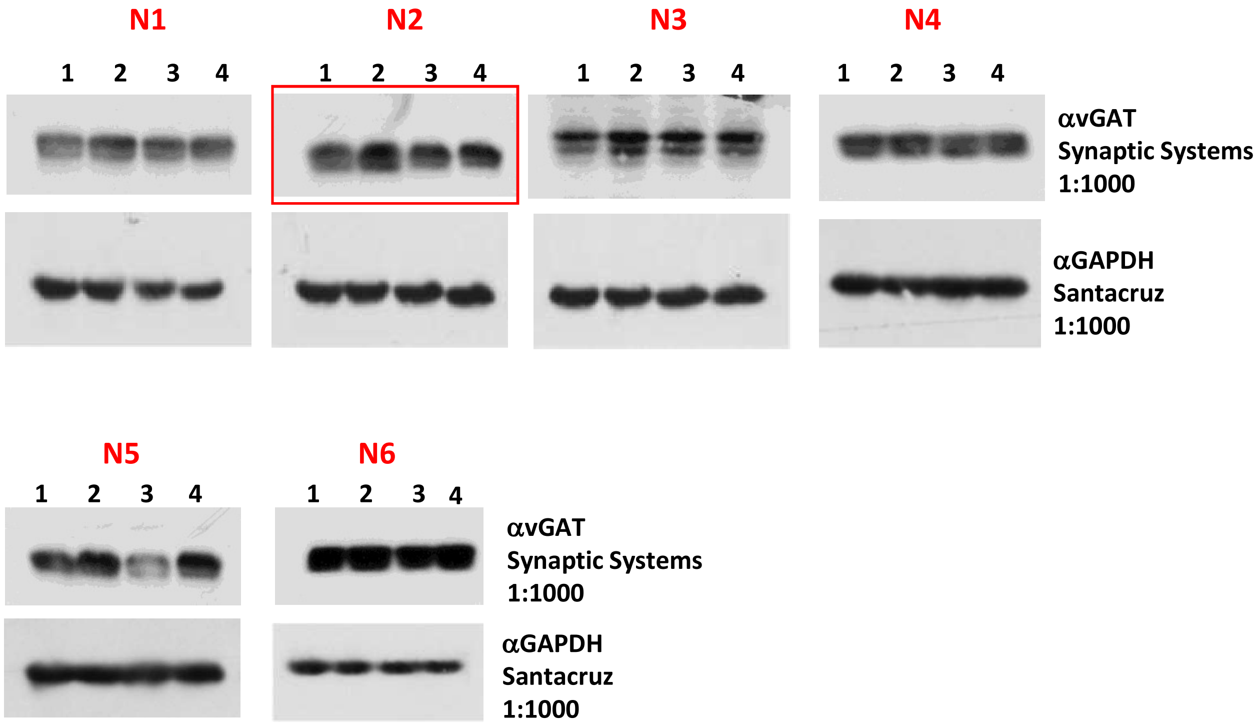

Red square indicates the representative blot of Figure 9D.

Layout legend: 1 is for NEG veh
2 is for NEG 4AP
3 is for ODN veh
4 is for ODN 4AP
