# Supplementary material for: REST/NRSF drives homeostatic plasticity of inhibitory synapses in a target-dependent fashion
Source: eLife. 2021 Dec 2;10:e69058. doi: 10.7554/eLife.69058 (PMC8639147; doi:10.7554/eLife.69058)
Supplement: Figure 9—figure supplement 1—source data 1. [file elife-69058-fig9-figsupp1-data1.pdf]

Figure 9-figure supplement 1

Figure 9A-fig suppl 1 (left panel)

	NEG/veh				NEG/4AP				ODN/veh				ODN/4AP			
	1hrs	6hrs	12hrs	24hrs	1hrs	6hrs	12hrs	24hrs	1hrs	6hrs	12hrs	24hrs	1hrs	6hrs	12hrs	24hrs
	0.91222	0.82369	0.90872	0.94572	0.71937	0.99542	1.24067	0.91877	1.60419	1.25164	0.78551	0.70845	0.99308	1.05598	1.15945	1.39286
	0.92122	0.99998	1.1627	0.96656	0.9408	1.02892	1.25607	1.60056	1.16341	1.08581	1.17122	1.09985	0.91255	1.13622	1.00659	0.9622
	1.08561	1.17633	0.92858	0.84917	2.21616	0.68806	1.55762	0.99308	1.21669	0.87553	0.72622	0.84158	1.24935	1.03045	1.16659	0.77533
	0.99317	0.74393	1.19886	1.34968	1.11233	0.77554	0.87208	0.99209	0.87623	0.75845	0.80364	1.16146	0.65175	0.83877	0.90288	0.71652
	0.91122	0.7885	0.887	0.87242	1.02369	1.20555	1.18601	1.23911	0.92329	1.16746	1.07993	1.1268	1.10524	0.99561	0.73825	1.01191
	0.78582	0.82775	0.80988	0.90281	0.92739	1.05767	0.75712	0.95754	1.14883	1.0029	0.79516	0.751	0.87049	0.73728	0.68708	1.38092
**N**	**6**	**6**	**6**	**6**	**6**	**6**	**6**	**6**	**6**	**6**	**6**	**6**	**6**	**6**	**6**	**6**
**Media**	**0.9349**	**0.8934**	**0.9826**	**0.9811**	**1.1566**	**0.9585**	**1.1449**	**1.1169**	**1.1554**	**1.0236**	**0.8936**	**0.9482**	**0.9637**	**0.9657**	**0.9435**	**1.0400**
SD	0.0996	0.1637	0.1591	0.1858	0.5353	0.1917	0.2893	0.2625	0.2596	0.1840	0.1840	0.2040	0.2055	0.1487	0.2049	0.2906
**SE**	**0.0407**	**0.0668**	**0.0649**	**0.0759**	**0.2185**	**0.0783**	**0.1181**	**0.1071**	**0.1060**	**0.0751**	**0.0751**	**0.0833**	**0.0839**	**0.0607**	**0.0836**	**0.1186**

Figure 9A-fig suppl 1 (right panel)

	NEG/veh				NEG/4AP				ODN/veh				ODN/4AP			
	1hrs	6hrs	12hrs	24hrs	1hrs	6hrs	12hrs	24hrs	1hrs	6hrs	12hrs	24hrs	1hrs	6hrs	12hrs	24hrs
	0.92609	0.77964	0.88413	0.91778	1.23221	1.06763	1.45234	0.97981	0.85608	1.02005	1.06745	0.87664	1.07471	0.7536	1.39346	1.95776
	0.96789	1.24743	0.83084	1.13453	0.86242	1.04745	1.01513	0.82646	0.98648	0.73782	0.88758	0.89949	1.26612	0.95359	1.10083	1.22161
	0.91397	0.80662	1.15371	0.9477	1.18142	0.93776	1.26448	0.86742	1.435	0.85926	0.89277	0.9656	0.78744	1.08291	1.1418	0.81999
	1.11814	1.35667	0.81668	0.65775	1.36168	0.69605	0.91816	1.03376	1.20555	0.94678	1.31518	0.91172	1.08375	1.02348	1.15737	0.85327
	1.09337	0.83671	1.20348	1.3085	1.17548	1.21095	1.09005	1.21289	0.99489	1.14949	0.78221	1.27619	1.1318	0.75873	0.90109	1.23812
	0.90885	0.85351	0.97984	0.97826	1.23178	1.21605	0.9736	0.75572	1.1737	0.99503	0.919	0.75686	1.36052	0.98566	0.94478	0.95636
**N**	**6**	**6**	**6**	**6**	**6**	**6**	**6**	**6**	**6**	**6**	**6**	**6**	**6**	**6**	**6**	**6**
**Media**	**0.9881**	**0.9801**	**0.9781**	**0.9908**	**1.1742**	**1.0293**	**1.1190**	**0.9460**	**1.1086**	**0.9514**	**0.9774**	**0.9478**	**1.1174**	**0.9263**	**1.1066**	**1.1745**
SD	0.0938	0.2530	0.1663	0.2190	0.1668	0.1945	0.2027	0.1654	0.2058	0.1414	0.1892	0.1751	0.1966	0.1387	0.1758	0.4234
**SE**	**0.0383**	**0.1033**	**0.0679**	**0.0894**	**0.0681**	**0.0794**	**0.0827**	**0.0675**	**0.0840**	**0.0577**	**0.0772**	**0.0715**	**0.0803**	**0.0566**	**0.0718**	**0.1728**

Figure 9-figure supplement 1

Figure 9A-fig suppl 1 (left panel)			
Tukey's multiple comparisor	Significant	Summary	P Value
1hrs			
NEG/veh vs. NEG/4AP	No	ns	0.393
NEG/veh vs. ODN/veh	No	ns	0.398
NEG/veh vs. ODN/4AP	No	ns	0.997
NEG/4AP vs. ODN/veh	No	ns	> 0.9999
NEG/4AP vs. ODN/4AP	No	ns	0.517
ODN/veh vs. ODN/4AP	No	ns	0.522
6hrs			
NEG/veh vs. NEG/4AP	No	ns	0.966
NEG/veh vs. ODN/veh	No	ns	0.788
NEG/veh vs. ODN/4AP	No	ns	0.955
NEG/4AP vs. ODN/veh	No	ns	0.966
NEG/4AP vs. ODN/4AP	No	ns	> 0.9999
ODN/veh vs. ODN/4AP	No	ns	0.976
12hrs			
NEG/veh vs. NEG/4AP	No	ns	0.654
NEG/veh vs. ODN/veh	No	ns	0.920
NEG/veh vs. ODN/4AP	No	ns	0.992
NEG/4AP vs. ODN/veh	No	ns	0.283
NEG/4AP vs. ODN/4AP	No	ns	0.479
ODN/veh vs. ODN/4AP	No	ns	0.984
24hrs			
NEG/veh vs. NEG/4AP	No	ns	0.767
NEG/veh vs. ODN/veh	No	ns	0.995
NEG/veh vs. ODN/4AP	No	ns	0.975
NEG/4AP vs. ODN/veh	No	ns	0.625
NEG/4AP vs. ODN/4AP	No	ns	0.946
ODN/veh vs. ODN/4AP	No	ns	0.913

Figure 9A-fig suppl 1 (right panel)			
Tukey's multiple comparisons t	Significant	Summary	P Value
1hrs			
NEG/veh vs. NEG/4AP	No	ns	0.405
NEG/veh vs. ODN/veh	No	ns	0.742
NEG/veh vs. ODN/4AP	No	ns	0.698
NEG/4AP vs. ODN/veh	No	ns	0.946
NEG/4AP vs. ODN/4AP	No	ns	0.964
ODN/veh vs. ODN/4AP	No	ns	1.000
6hrs			
NEG/veh vs. NEG/4AP	No	ns	0.976
NEG/veh vs. ODN/veh	No	ns	0.995
NEG/veh vs. ODN/4AP	No	ns	0.969
NEG/4AP vs. ODN/veh	No	ns	0.914
NEG/4AP vs. ODN/4AP	No	ns	0.823
ODN/veh vs. ODN/4AP	No	ns	0.997
12hrs			
NEG/veh vs. NEG/4AP	No	ns	0.639
NEG/veh vs. ODN/veh	No	ns	> 0.9999
NEG/veh vs. ODN/4AP	No	ns	0.703
NEG/4AP vs. ODN/veh	No	ns	0.635
NEG/4AP vs. ODN/4AP	No	ns	1.000
ODN/veh vs. ODN/4AP	No	ns	0.699
24hrs			
NEG/veh vs. NEG/4AP	No	ns	0.982
NEG/veh vs. ODN/veh	No	ns	0.984
NEG/veh vs. ODN/4AP	No	ns	0.416
NEG/4AP vs. ODN/veh	No	ns	> 0.9999
NEG/4AP vs. ODN/4AP	No	ns	0.228
ODN/veh vs. ODN/4AP	No	ns	0.234

Figure 9-figure supplement 1

	Figure 9C-fig suppl 1 (left panel)				Figure 9C-fig suppl 1 (right panel)			
	NEG/veh	NEG/4AP	ODN/veh	ODN/4AP	NEG/veh	NEG/4AP	ODN/veh	ODN/4AP
	86.001	76.379	106.711	112.879	82.76468	104.6871	95.95869	108.8301
	118.556	127.860	99.019	111.791	130.162	130.1843	111.8836	102.7617
	107.262	111.554	96.296	101.401	98.40442	102.4528	117.642	93.24356
	88.181	107.929	106.988	102.406	88.66893	121.4297	114.3083	96.79023
	125.326	87.512	123.142	69.395	138.609	96.08977	65.41962	37.47482
	74.674	76.480	65.438	66.391	61.39098	82.66101	115.3505	84.4653
					100	119.7174	149.4082	143.4943
					116.9293	67.88343	82.24727	53.48214
					83.07072	67.00447	68.6636	52.5048
N	6	6	6	6	9	9	9	9
Media	100.0000	97.9522	99.5989	94.0439	100.0000	99.1233	102.3202	85.8941
SD	20.0775	21.0451	19.1711	20.8137	24.7292	22.9572	26.9138	33.1906
SE	8.1966	8.5916	7.8266	8.4971	8.2431	7.6524	8.9713	11.0635

Figure 9-figure supplement 1

Figure 9C-fig suppl 1 (left panel)			
**Tukey's multiple comparisons test**	Significan	Summary	P Value
NEG :veh vs. NEG :4AP	No	ns	0.998
NEG :veh vs. ODN :veh	No	ns	> 0.9999
NEG :veh vs. ODN :4AP	No	ns	0.9561
NEG :4AP vs. ODN :veh	No	ns	0.999
NEG :4AP vs. ODN :4AP	No	ns	0.9868
ODN :veh vs. ODN :4AP	No	ns	0.9639
Figure 9C-fig suppl 1 (right panel)			
**Tukey's multiple comparisons test**	Significan	Summary	P Value
NEG :veh vs. NEG :4AP	No	ns	0.9999
NEG :veh vs. ODN :veh	No	ns	0.9979
NEG :veh vs. ODN :4AP	No	ns	0.6926
NEG :4AP vs. ODN :veh	No	ns	0.9945
NEG :4AP vs. ODN :4AP	No	ns	0.7328
ODN :veh vs. ODN :4AP	No	ns	0.582

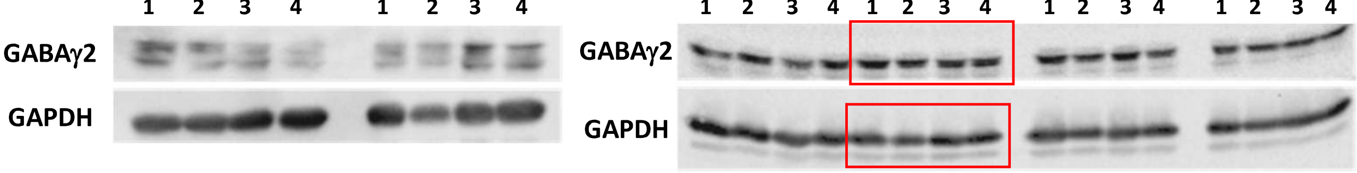

N7+N8+N9

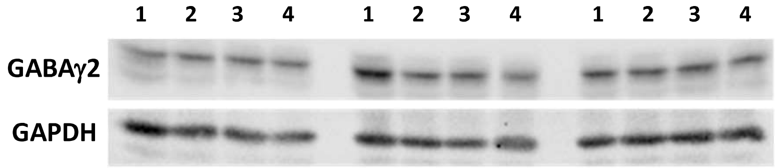

antiGABA receptor γ2 subunit SySy 1:1000

antiGAPDH Santacruz 1:1000

Red square indicates the representative blot of the figure;

Layout legend: 1 is for NEG veh
2 is for NEG 4AP
3 is for ODN veh
4 is for ODN 4AP

Figure 9 – figure supplement 1 - panel B-GABA receptor α2 subunit dataset

N1+N2+N3+N4

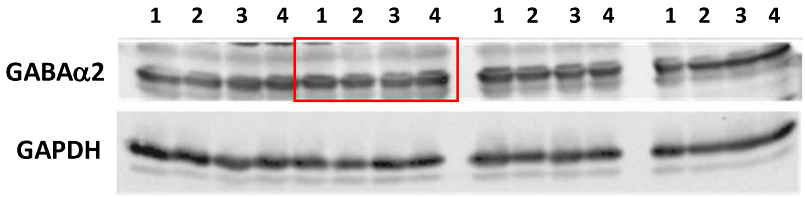

N5+N6

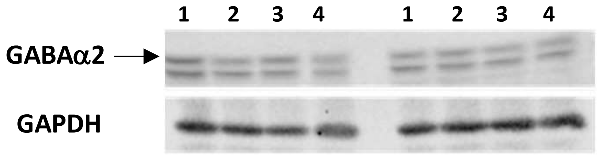

antiGABA receptor α2 subunit SySy 1:1000

Red square indicates the representative blot of the figure;

Layout legend: 1 is for NEG veh
2 is for NEG 4AP
3 is for ODN veh
4 is for ODN 4AP

For GABA receptor α2 subunit GAPDH are the same as γ2 subunit because it has been analyzed on the same lysates.
